# Size regulation of multiple organelles competing for a limiting subunit pool

**DOI:** 10.1371/journal.pcbi.1010253

**Published:** 2022-06-17

**Authors:** Deb Sankar Banerjee, Shiladitya Banerjee

**Affiliations:** Department of Physics, Carnegie Mellon University, Pittsburgh, Pennsylvania, United States of America; University of Connecticut School of Medicine, UNITED STATES

## Abstract

How cells regulate the size of intracellular structures and organelles is a longstanding question. Recent experiments suggest that size control of intracellular structures is achieved through the depletion of a limiting subunit pool in the cytoplasm. While the limiting pool model ensures organelle-to-cell size scaling, it does not provide a mechanism for robust size control of multiple co-existing structures. Here we develop a generalized theory for size-dependent growth of intracellular structures to demonstrate that robust size control of multiple intracellular structures, competing for a limiting subunit pool, is achieved via a negative feedback between the growth rate and the size of the individual structure. This design principle captures size maintenance of a wide variety of subcellular structures, from cytoskeletal filaments to three-dimensional organelles. We identify the feedback motifs for structure size regulation based on known molecular processes, and compare our theory to existing models of size regulation in biological assemblies. Furthermore, we show that positive feedback between structure size and growth rate can lead to bistable size distribution and spontaneous size selection.

## Author summary

Organelle size control is essential for the proper physiological functioning of eukaryotic cells, but the underlying mechanisms of size regulation remain poorly understood. By developing a general theory for organelle size control, we show that robust size control of intracellular structures and organelles is achieved via a negative feedback between individual organelle size and their net growth rates. This design principle not only describes size maintenance of single organelles, but also ensures size stability of multiple co-existing organelles that are built from a limiting pool of subunits. Our results delineate the role of limiting pool as a size scaling mechanism rather than a size control mechanism, supporting the idea that negative feedback control of organelle size via depletion of a limiting subunit pool is not sufficient to maintain the size of multiple competing organelles. In the case of positive feedback between organelle size and growth rate, our model reproduces phenomena such as bistability in organelle size distribution and spontaneous emergence of cell polarity.

## Introduction

Eukaryotic cells are composed of a wide diversity of macromolecular assemblies, from linear protofilaments to networks of cytoskeletal polymers and complex three-dimensional organelles such as the centrosomes and the nucleus. The cytoplasmic pool of proteins constitutes the building blocks for intracellular organelles, whose sizes are often commensurate with cell size. Despite continuous turnover in their component parts, intracellular structures are maintained at a precise size through dynamic balance between subunit assembly and disassembly [1]. An outstanding challenge is to identify the design principles through which cells achieve robust size regulation of multiple co-existing structures that are assembled from a limiting pool of molecular building blocks in the cytoplasm.

Studies in recent years have focused on understanding the mechanisms for size control of individual cellular structures such as the eukaryotic flagella [2, 3], actin cables [4], mitotic spindles [5–7], centrosomes [8, 9], as well as the nuceloli [10] and the nucleus [11]. A simple model that explains size control of these dynamic structures is the *limiting pool model* [1], where structures grow by depleting the pool of available subunits in the cytoplasm. As a result, growth rate of structures decreases with increasing assembly size, and a steady-state size is reached when the rate of assembly balances the rate of disassembly of incorporated material. Since structure size is determined by the amount of available subunits in the cytoplasm, which in turn scales with cell size, the limiting pool model naturally captures the scaling of structure size with cell size. However, the limiting pool fails to capture size regulation of multiple competing structures [12, 13], due to the absence of an underlying mechanism for sensing individual structure size. Failure of the limiting pool mechanism in determining the size of multiple structures suggests that additional feedback design principles are necessary for robust size control of intracellular structures. In this work we develop a theory for size-dependent growth of intracellular structures and organelles that assemble from a limiting pool of building blocks in the cellular cytoplasm. Using this theory, we uncover the feedback motifs between structure size and growth rate that are necessary for robust size maintenance of multiple structures competing for a shared subunit pool. We specifically consider the size regulation of structures that grow via subunit addition and removal processes, and do not consider multi-compartment organelles such as the golgi body or the endoplasmic reticulum that grow via budding and fusion where the chemical composition of the structures may play crucial roles.

In the noisy cellular environment, intracellular structures grow from a cytoplasmic subunit pool via stochastic assembly and disassembly processes [14]. For simplicity, we first consider a deterministic description for the growth of *M* structures that incorporate material from *N*_av_ available subunits with assembly rate *K*^on^ and disassembly rate *K*^off^. Dynamics of size of the *i*^*th*^ structure (*i* = 1..*M*), *n*_*i*_ (expressed as the number of subunits), is given by:

$$\begin{array}{c} {\dot{n}}_{i}=K^{\text{on}}\left(\rho \right)-K^{\text{off}} \end{array}$$ (1)

where *ρ* is the concentration of subunits in the cytoplasm, given by *ρ* = *N*_av_/*V*, with *V* the cell volume. The rates *K*^on, off^ can in general be functions of structure size and chemical composition. In the canonical limiting pool model [1], *K*^on^ = *k*^+^*ρ*, *K*^off^ = *k*^−^, where $k_{i}^{+}$ and $k_{i}^{-}$ are the bare rates of assembly and disassembly of the *i*^*th*^ structure. If the cytoplasmic concentration of subunits is maintained at a constant homeostatic value ($\dot{\rho }=0$) [15, 16], size control is not achieved except when *ρ* is fine tuned to a critical value *ρ* = *ρ*_*c*_ = *k*^−^/*k*^+^. Growth is unbounded for *ρ* > *ρ*_*c*_ and the assembly degrades for *ρ* < *ρ*_*c*_. By contrast, in the presence of a limiting pool, the total amount of subunits $N=N_{\text{av}}+\sum_{i=1}^{M}n_{i}$ is constant. In this case, the assembled structure reaches a steady-state size *n* = *N* − (*k*^−^/*k*^+^)*V*, only for *M* = 1.

However, when multiple structures are assembled from a shared subunit pool (*M* > 1), Eq (1) yields a system of under-determined rate equations with no unique solution for the steady-state size of the individual assemblies when the subunit pool is limited (see S1 and S2 Texts for details). This indeterminacy manifests as large size fluctuations (S1 Fig) in a stochastic description [13], arising from building block transfer (via assembly and disassembly) between individual structures. The underlying reason is that the limiting pool model does not provide a mechanism to sense the individual size of the structures. Rather, the limiting pool mechanism operates by sensing the size of the available subunit pool to provide a system size-dependent feedback.

In the limiting pool model for *M* > 1, departure from size control of individual structures is manifested either as large anticorrelated size fluctuations (for identical growth rates), or the faster growing structure ending up incorporating all the subunits [13]. To ensure robust size control, a negative feedback is required between the growth rate and the size of individual structures, which is lacking in the canonical limiting pool model. Such feedback motifs can be realized when the net growth rate of individual structures decreases with increasing size. In fact, size-dependent assembly and disassembly rates have been reported in many cases of single filament growth, including in Chlamydomonous flagella [3], microtubules [17, 18], as well as filamentous actin (F-actin) [19]. Size-dependent growth models have also been developed for multi-compartment membrane-bound organelles that grow via budding and fusion of vesicles, where growth dynamics depends on the local chemical composition of the organelle [20]. Here we develop a theory for size-dependent growth of multiple intracellular structures that grow via subunit addition and removal processes, and compete for a limiting pool of building blocks in the cellular cytoplasm. We specifically explore the effect of size-dependent feedback motifs on organelle growth dynamics to uncover the nature of feedback that may guarantee robust control of organelle size.

## Results

### Theory of size-dependent growth of intracellular structures in a limiting subunit pool

We first develop a minimal mathematical model for the assembly of an intracellular structure from a limiting subunit pool, where the subunits undergo binding and unbinding kinetics with rates given by *K*^on^ and *K*^off^ respectively. In our model, the assembly and disassembly rates depend on the size of the individual structures and are given by

$$\begin{array}{ccc} K^{\text{on}}\left(n\right) & = & k^{+}\left(N-n\right){\left(1+n\right)}^{-\alpha }/V\;,\\ K^{\text{off}}\left(n\right) & = & k^{-}n^{\beta }\;, \end{array}$$ (2)

where *N* is the total amount of subunits, *k*^+^ and *k*^−^ are the bare assembly and disassembly rates, *N* − *n* = *N*_av_ is size of the pool of free subunits. The coefficients *α* and *β* can take on both positive and negative values, and represent the strengths of the size-dependent feedback that can arise due to active molecular processes or the geometry of the structures grown. A more general choice for the size-dependent growth rate could be a polynomial function of *n* of arbitrary degree. Here we consider a power-law form for analytical tractability. The power-law form for the growth rate also makes it easier to infer the nature of the size-dependent feedback, which plays a vital role in organelle size regulation as discussed below. Negative feedback control of growth is realized for *α* > 0 (assembly rate decreases with size) and/or *β* > 0 (disassembly rate increases with size). Conversely, size-dependent positive feedback can be described with *α* < 0 and/or *β* < 0. These simple power law forms for the assembly and disassembly rates allow for analytical tractability of our minimal model, but results in a mathematical singularity for *β* < 0, where the disassembly rate encounters a divergence as the structure size approaches zero. While this singularity has to be appropriately regularized in our analysis, we note that the regime *β* < 0 is not biophysically relevant. To the best of our knowledge, no known biological growth processes exhibit positive feedback from size-dependent disassembly.

We assume that the subunit pool is well mixed in the cytoplasm such that subunit diffusion is much faster compared to the growth process (see S3 Text for the role of diffusion). A conceptual understanding of the growth process can be gained by dynamical systems analysis of the following rate equation for the time evolution of a single structure of size *n*:

$$\begin{array}{c} \frac{dn}{dt}=K^{\text{on}}\left(n\right)-K^{\text{off}}\left(n\right)\;. \end{array}$$ (3)

From the above rate equation, it follows that there is a unique and stable steady-state size of the structure for *α* + *β* ≥ 0 (S2 Text). By contrast when *α* + *β* < 0 the system exhibits bistability with two stable fixed points (see S2 Text).

To study the robustness of size control, it is necessary to go beyond a simple deterministic description of average size, and examine how variations in size are controlled. To this end, we implement a stochastic model in which the chemical master equation for the probability *P*(*n*, *t*) of assembling a structure of size *n* at time *t* is given by,

$$\begin{array}{cc} \frac{\text{d}P(n,t)}{\text{dt}} & =K^{\text{on}}\left(n-1\right)P\left(n-1,t\right)+K^{\text{off}}\left(n+1\right)P\left(n+1,t\right)\\ & -(K^{\text{on}}\left(n\right)+K^{\text{off}}\left(n\right))P\left(n,t\right). \end{array}$$

The above equation can be solved at steady state using the detailed balance condition: *k*^−^*n*^*β*^*P*(*n*) = *k*^+^(*N* − *n* + 1)(*n*)^−*α*^*P*(*n* − 1)/*V*. The steady-state size distribution, in a system of unit volume, is given by

$$\begin{array}{c} P\left(n\right)=\frac{1}{C_{N}}(\frac{\kappa^{n}N!}{\left(N-n\right)!{\left(n!\right)}^{\alpha +\beta }}) \end{array}$$ (4)

with $\kappa =\frac{k^{+}}{k^{-}}$ and the normalization constant $C_{N}=\sum_{n=0}^{N}\kappa^{n}N!/\left[\left(N-n\right)!{\left(n!\right)}^{\alpha +\beta }\right]$. For *α* + *β* ≥ 0 we get a unimodal size distribution steady-state, with a mean size that is stable to perturbations (S3 Fig). For *α* + *β* < 0, the size distribution is bimodal, indicative of a bistable system (S4 Fig and S2 Text). We return to this case later.

### Size regulation of two competing structures

**Fig 1:**
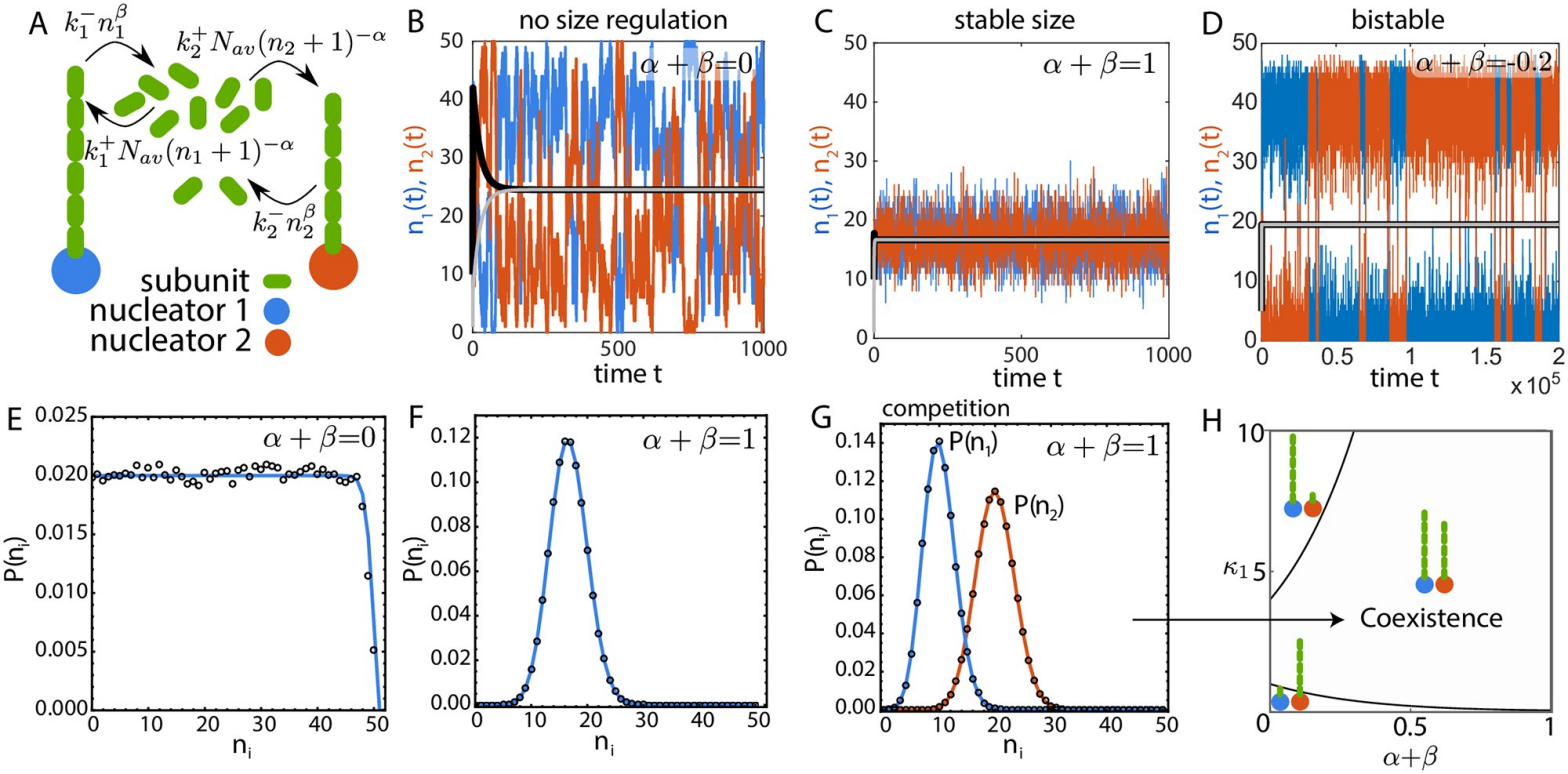
Size regulation of two structures grown from a shared subunit pool. (A) Schematic of two filaments growing from a shared pool of monomers where the assembly and disassembly rates depend on their individual size. (B-D) Size dynamics of two identical filaments (*κ*_1_ = *κ*_2_ = *κ* = 1 for B and C) obtained from stochastic simulations in three distinct growth regimes: (B) *α* + *β* = 0 (for deterministic solution *α* = −1 and *β* = 1), (C) *α* + *β* = 1 (*α* = 0, *β* = 1) and (D) *α* + *β* = −0.2, *κ* = 0.0022 (*α* = −0.2, *β* = 0). The black (*n*_1_) and gray (*n*_2_) solid lines are the deterministic solutions to the rate equations of size. The deterministic solution correctly captures the mean size dynamics for *α* + *β* = 1, but fails to predict the essential features of size dynamics in the two other cases. (E-F) Size distribution of two identical structures for (E) *α* + *β* = 0 and (F) *α* + *β* = 1. Open circles represent solution from stochastic simulation and the solid line represents the analytical solution to the chemical master equation. (G) Size distribution for two competing structures with *α* + *β* = 1 and *κ*_1_ = 2*κ*_2_ = 1, obtained from analytical solution to the master equation (solid line) and stochastic simulations (open circles). (H) Phase diagram showing the co-existence of two competing structures over a broad range of parameter space in the model, with *κ*_2_ = 2. Coexistence phase is defined as both structures having mean size larger than one subunit. For all results in (A-H) *V* = 1 and *N* = 50.

To elucidate the emergence of robust size control of multiple structures via size-dependent negative feedback on growth, we consider two assemblies growing from a shared pool of *N* subunits (Fig 1A). At time *t*, the size of the *i*^*th*^ assembly (*i* = 1, 2) is given by *n*_*i*_(*t*), the number of incorporated subunits. The assembly and disassembly rates of the *i*^*th*^ structure is $K^{\text{on}}=k_{i}^{+}N_{\text{av}}{\left(1+n_{i}\right)}^{-\alpha }/V$ and $K^{\text{off}}=k_{i}^{-}n_{i}^{\beta }$. Here, $k_{i}^{\pm }$ denote the bare assembly and disassembly rates of the *i*^*th*^ structure, and *N*_av_ = *N* − (*n*_1_ + *n*_2_) is the total amount of available subunits. The state of the system can be characterized by the joint probability distribution, *P*(*n*_1_, *n*_2_, *t*), which is the probability that the size of the structure *i* (*i* = 1, 2) is *n*_*i*_ at time *t*. The time evolution of the probability distribution is governed by a chemical master equation (S2 Text) that can be solved to obtain the steady-state joint probability distribution in a system of unit volume (*V* = 1),

$$\begin{array}{c} P\left(n_{1},n_{2}\right)=P\left(0,0\right)\frac{\kappa_{1}^{n_{1}}\kappa_{2}^{n_{2}}\;N!\;}{{\left(n_{1}!\right)}^{\alpha +\beta }{\left(n_{2}!\right)}^{\alpha +\beta }N_{\text{av}}!}\;, \end{array}$$ (5)

where $\kappa_{1}=k_{1}^{+}/k_{1}^{-}$, $\kappa_{2}=k_{2}^{+}/k_{2}^{-}$, and *P*(0, 0) is a normalization constant. The size distributions of the individual structures, *P*(*n*_1_) and *P*(*n*_2_), can be obtained from the joint distribution by summing over all possible sizes of the other structure, as derived in S2 Text. In the following, we use stochastic simulations (Gillespie algorithm, Methods) to compute the time evolution of the size of individual structures, and compare them to the dynamics predicted by the deterministic rate equations (Eq 1). We note that the rate equations (Eq 1) do not simply follow from the first moment of the chemical master equation, and should be interpreted as an approximate deterministic model. Due to the nonlinearity of the system, the equation for the mean size is coupled to higher order moments and cannot be simply deduced for all values of *α* and *β* (see S2 Text).

The case *α* = 0 and *β* = 0 corresponds to the canonical limiting pool model (Eq 1) where the assembly rate is proportional to the available pool size and the disassembly rate is constant [1]. In this case, the deterministic equations do not have a unique solution and stochastic simulations predict large anticorrelated size fluctuations as the subunit pool is depleted (S2 Text and S1 Fig). The class of models with *α* + *β* = 0 also fails to regulate the size of individual structures similar to the limiting pool model. Although the models in this class exhibit size-dependent assembly and disassembly rates, there is no overall negative feedback control of structure size. For example, in the case *α* = −1 and *β* = 1, the positive feedback control of assembly is nullified by a negative feedback in disassembly. Lack of size regulation for *α* + *β* = 0 manifests as large anticorrelated size fluctuations of two competing (but identical) structures (Fig 1B), resulting in an almost uniform size distribution (Fig 1E). In this particular parameter regime (*α* + *β* = 0, *β* = 1), the deterministic equations predict the existence of a stable node, while in the presence of stochasticity there is no size regulation of individual structures (Fig 1B and S2 Fig).

By contrast, for *α* + *β* > 0, there is an overall negative feedback control of growth that ensures robust size control of multiple structures. To illustrate this, we simulate the growth dynamics of two identical filaments competing for the same subunit pool, for the specific case *α* + *β* = 1 (Fig 1C and 1F). The individual filaments assume a well-defined mean length (Fig 1C), with the standard deviation in size smaller compared to the mean (Fig 1F). In this case (*α* + *β* > 0), the deterministic equations predict the existence of a stable node (S3 Fig) and correctly captures the mean length dynamics (Fig 1C).

In the scenario when *α* + *β* < 0, there is an overall positive feedback resulting in autocatalytic growth and bistable size distribution. In this case, the two identical structures initially grow at equal rates, but due to transient size differences arising from stochastic fluctuations, the bigger structure ends up assimilating all the subunits. Consequently, stochastic fluctuations can make the bigger structure lose enough subunits at the expense of growth of the smaller structure. Thus, stochastic simulations result in dynamic switching between two stable states (Fig 1D), which is not captured by the deterministic rate equations (S4 and S5 Figs).

Apart from the existence of a well-defined mean steady-state size for *α* + *β* > 0, co-existence of multiple competing non-identical structures is an important aspect of biological size regulation. It is relevant when there are nucleators with different assembly and disassembly rate constants, assembling multiple structures from the same subunit pool [21]. In the limiting-pool regime, even a small difference in assembly or disassembly rate constants will lead to a “winner-takes-all” scenario, where the structure with a higher net growth ends up taking all the subunits [13]. A negative feedback control of growth rate ensures stable co-existence of multiple competing structures with mean size commensurate with the difference in their growth rates (Fig 1G). This co-existence regime occurs over a wide range of the parameter space (Fig 1H) for *α* + *β* > 0, reflecting it to be a robust feature of negative feedback control of size.

It is important to note that the different growth regimes described above are only meaningful when the net growth rate *κ* (or the subunit concentration) is high enough. Below a threshold growth rate, *κ* < *κ*_0_, the structure size is small (of the order of unity) and the dynamics are characterised by large size fluctuations (coefficient of variation *CV* > 1) even in presence of strong negative feedback (S6 Fig). The size distribution in this regime is very close to an exponential distribution (S6 Fig). We define *κ*_0_ as the growth rate for which *CV* = 1. Both the mean size and the standard deviation increases with increasing *κ* and *N*, with the *CV* decreasing with increasing *κ* (S6 Fig). The loss of size regulation at smaller growth rates (*κ* < *κ*_0_) occurs for all values of *α* + *β* (S6 Fig). We note that this regime of growth can also be observed below a critical subunit concentration for which the deterministic growth rate $\dot{n}∼0$, but stochastic growth gives rise to small transient structures with a large CV in the size distribution.

The results presented above for two structures (Fig 1) can be easily generalized to multiple structures (*M* > 2) growing from the same subunit pool (S7 Fig). As discussed in detail in S4 Text, introducing more structures does not introduce any new interactions and the qualitative features of the growth in the different feedback regimes remain unchanged for any value of *M* (S8 Fig). Since analytical computation of size distribution of individual structures become cumbersome for larger values of *M*, we use Gillespie simulations to evolve the size dynamics of multiple structures.

### Comparisons to specific biological systems—Filament length regulation

**Fig 2:**
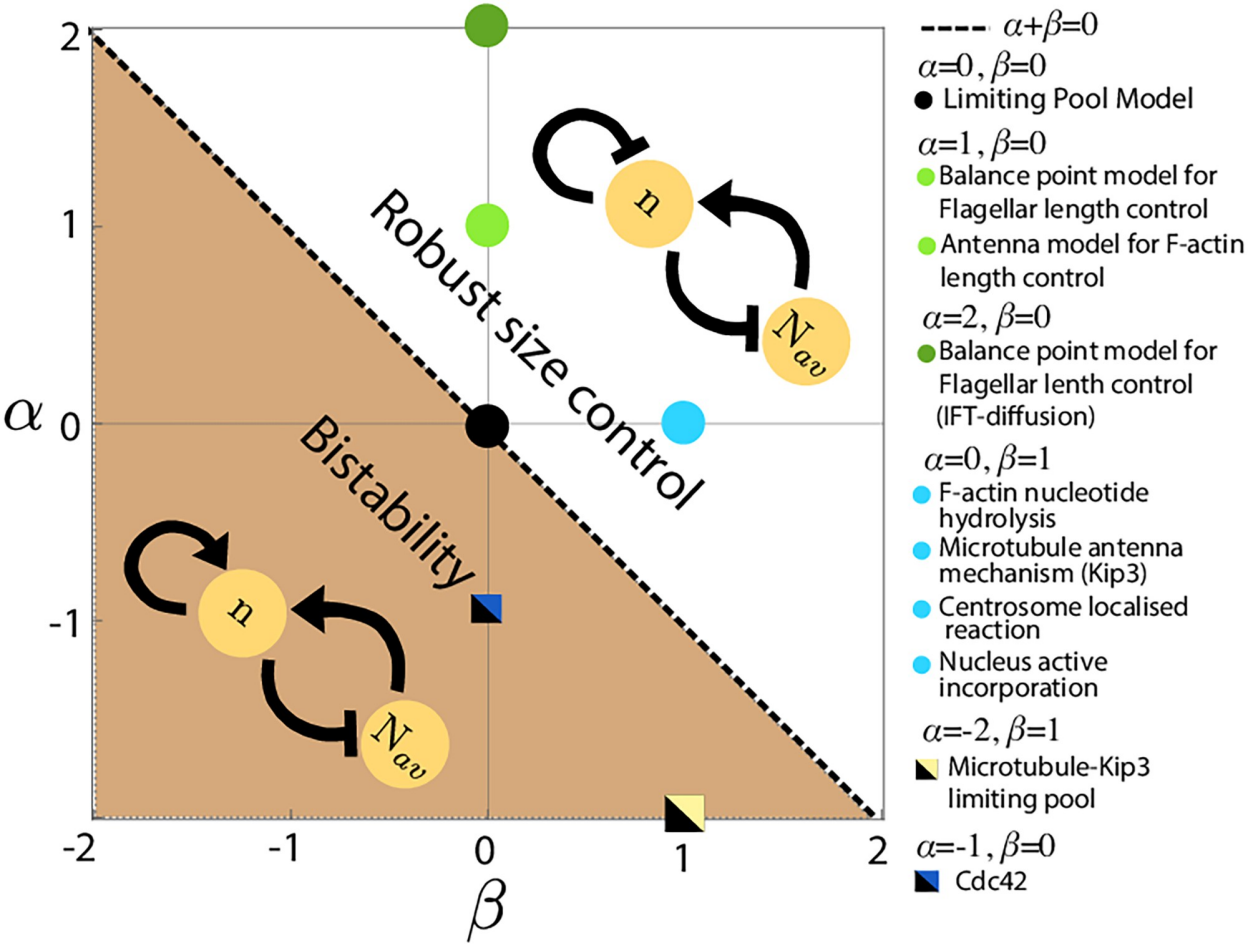
Phase diagram of the size-dependent growth model. Phase diagram of the size-dependent growth model in *α*-*β* plane, showing the different regimes of size control. *α* + *β* > 0 defines the regime of negative autoregulation of growth which guarantees robust size control. Positive autoregulation of growth (*α* + *β* < 0) gives rise to bistable size distribution. The phase boundary *α* + *β* = 0 corresponds to a class of models where there is no size regulation of individual structures, similar to the limiting pool model (*α* = 0, *β* = 0). We map our model to size control mechanisms for a variety of intracellular structures, from linear filaments to organelles.

Size-dependent growth of structures is a commonly observed motif for size regulation in biology and it appears in many specific biomolecular systems that grow via subunit assembly and disassembly. Here we visit some of these examples to show how size-dependent assembly and disassembly rates in these intracellular structures can be captured by our minimal model for specific choices of the coefficients *α* and *β*. The case (*α*, *β*) = (0, 1) can be mapped to the length regulation of Microtubules and F-actin (see S5 Text for details), where the filament disassembly rate increases with increasing filament length (Fig 2, cyan solid circle).

In the antenna mechanism for microtubule length control, the kinesin Kip3 associates with microtubule monomers, walks towards the plus end of the filament and detaches from the end by removing microtubule monomers [22]. Over time, Kip3 molecules accumulate near the plus-end, leading to an effective linear length-dependent disassembly rate [23]. In the case of F-actin, chemical changes in subunit states, via nucleotide hydrolysis of bound monomers [24], can lead to a linear length-dependent disassembly rate [19] (S9 Fig). In addition, length-dependent F-actin disassembly could also arise through the action of the severing protein ADF/cofilin [25]. In all these cases, length-dependent disassembly via active molecular processes can stabilize the length of multiple filaments competing for the same monomer pool.

Similar to length-dependent disassembly processes, length-dependent assembly can also give rise to negative feedback control of structure size in many cases, such as in the growth of actin cables in yeast and eukaryotic flagella. Size regulation of actin cable via antenna mechanism [4] can be mapped to the case (*α*, *β*) = (1, 0) where the assembly rate decreases with structure size (Fig 2, light green dots). In the case of actin cable formation, Smy1 proteins get transported to actin barbed end and inhibit Formin activity to transiently halt the growth [26]. Longer filaments can bind and transport more Smy1 to the barbed end, creating a size dependent decrease in assembly rate [4]. Below we present models for a few specific organelles whose size regulation be understood using our proposed motifs for size-dependent feedback on growth rate.

### Length regulation of eukaryotic flagella

Flagellar growth in the biflagellate *Chlamydomonas reinhardtii* is a classic example of size regulation of multiple organelles assembled from a common cytoplasmic pool of building blocks [27–29]. Molecular mechanisms for flagellar length control remain an active area of research, with mathematical models suggesting that flagellum length dynamics is controlled by a length-dependent assembly process [2, 3, 30], or by a length-dependent disassembly mechanism [31].

**Fig 3:**
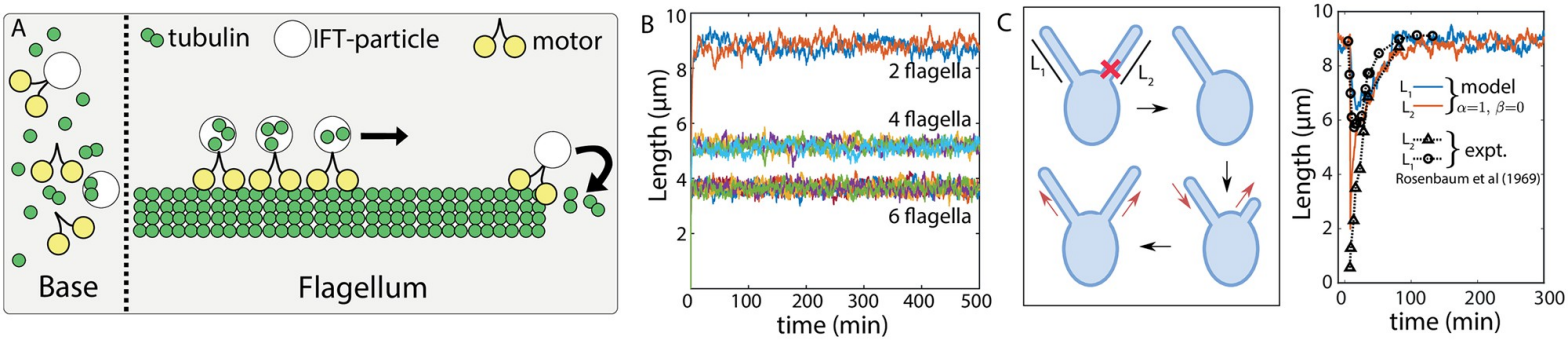
Flagellar size regulation. (A) Flagellum assembly in *C. reinhardtii* is regulated by IFT particles that incorporate tubulin dimers at the flagellum tip. This assembly process combined with the conservation of IFT amount in flagellum gives rise to an assembly rate that decreases with flagellum length. (B) Decrease in flagellar size with increasing number of flagella while the cell size is kept constant. (C, Left) We model the flagellar re-growth experiment [28], where one of the two flagella is amputated and regrowth is observed. The intact flagellum starts shrinking immediately after the amputation, indicating a shared pool of building blocks. (C, Right) Fit to the experimental data for the length dynamics of the two flagella with the growth curves from simulation show good quantitative agreement. The plotted time shows the duration after amputation. Parameters: tubulin concentration = 5 *μ*M, *α* = 1, *β* = 0, tubulin size = 10 nm, *k*^+^ = 120 *μ*m^3^ min^−1^, *k*^−^ = 100 min^−1^, *t*_0_ = 1000 min, production rate of new subunits *r*_*p*_ = 0.0016 min^−1^.

*C. reinhardtii* flagella grow from a shared pool of tubulins, which are carried and assembled via intraflagellar transport (IFT) particles at the tip of the flagellum [3] (Fig 3A). As the total amount of IFT particles on the flagellum remain constant over time [3, 30], IFT density at the flagellar tip is a decreasing function of length. This leads to a length-dependent assembly rate for the flagellum, inversely proportional to the flagellum length [2, 30, 32], corresponding to the case (*α*, *β*) = (1, 0) in our model (Fig 2).

We use the balance-point model proposed by Marshall et al. [2, 3, 32] to show that it is sufficient to regulate flagellar length in the multi-flagellate system in *C. reinhardtii*. A deterministic description of the system takes the form (*i* = 1, 2),

$$\begin{array}{c} {\dot{n}}_{i}=k_{i}^{+}(\frac{N-\sum_{j=1}^{2}n_{j}}{V}){\left(1+n_{i}\right)}^{-1}-k_{i}^{-} \end{array}$$ (6)

where *n*_*i*_ is the length of the *i*^*th*^ flagellum in tubulin numbers, *V* is the cell volume, *N* is total tubulin amount in the cell. The initial growth rate is independent of flagellar length, consistent with experimental data [28]. We use Gillespie algorithm (Methods) to simulate the stochastic system of multiple flagella grown from a shared pool of tubulins. Our simulations (Fig 3B) capture the experimentally reported phenomena that mean flagellar size decreases with increasing the number of flagella in a cell [12, 27, 33].

We further use our stochastic model to simulate the flagellar regrowth experiment (Fig 3C-left), where one of the two flagella is cut at *t* = *t*_0_ and the length dynamics for both the flagella are measured. Upon severing of one of the flagella, the intact flagellum starts shrinking initially whereas the amputated flagellum starts growing in response to the cut [28]. Both the flagella start growing after some time, eventually reaching a steady state-length that is similar to the original length of the flagellum [28]. The regrowth of the damaged flagellum is limited by the production of new subunits as inhibition of protein production generates two flagella with the same but smaller size [28]. To model this experiment, we first let the size of the two flagella (with lengths *L*_1_ and *L*_2_) reach a steady-state following the stochastic growth dynamics given in Eq 6. We model the amputation by letting *L*_2_ → 0 which results in reduction in the total number of building blocks *N* = *N*_*av*_ + *L*_1_ + *L*_2_, such that *N* → *N* − *L*_2_. Subunits are produced at a rate *r*_*p*_ to replenish the available subunit pool such that *N*_*av*_(*t*) = *N*_*av*_(*t*_0_) + *δN*(*t*). Dynamics of subunit production is given by:

$$\begin{array}{c} \dot{\delta N}=r_{p}\left(\Delta_{N}-\delta N\right)\;, \end{array}$$ (7)

where Δ_*N*_ (= *L*_2_) is the lost amount of subunits during the flagellum amputation and the initial condition is *δN* (*t*_0_) = 0. The timescale for fast shrinkage dynamics is governed by the rate constants *k*^±^. The slower process of length recovery is governed by the production rate of new subunits. We used a least-squared minimization process to determine the production rate *r*_*p*_ by fitting our model with the experimental data [27]. The final results are shown using a rescaled time *t* − *t*_0_. Our fitted model quantitatively captures the experimentally measured flagellar length dynamics (Fig 3C-right).

### Centrosome size regulation

#### Kinetic self-assembly model

**Fig 4:**
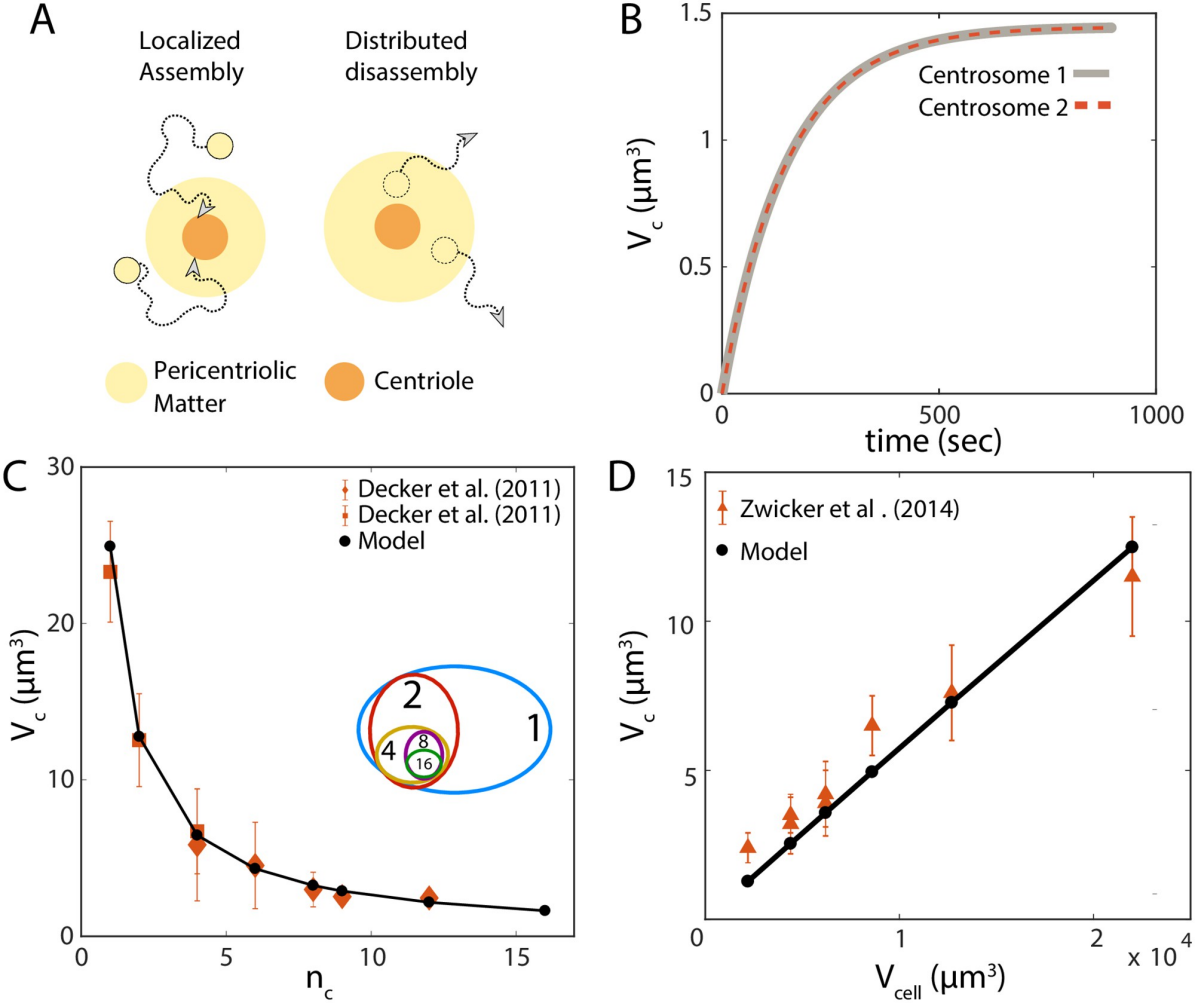
Centrosome growth by localised assembly and distributed disassembly. (A) Localized assembly around the centriole and disassembly throughout the pericentriolic matter generate a size-dependent disassembly rate which ensures robust size control. (B) Size dynamics of a pair of identical centrosomes that are initialized with equal size. Here *V*_cell_ = 5000 *μ*m^3^ (C) Centrosome volume decreases with increasing centrosome number, *n*_*c*_, during *C. elegans* embryonic development, where the embryo rapidly divides into many cells with decreasing cell size. Model: solid black line, Experimental data: orange. (D) Centrosome volume, *V*_*c*_, scales linearly with cell volume, *V*_cell_. Parameters: subunit concentration = 1.67 *μ*M, *α* = 0, *β* = 1, subunit size = 5.8 × 10^−7^ *μ*m^3^, *k*^+^ = 10^3^ *μ*m^3^min^−1^, *k*^−^ = 10^−3^min^−1^.

Centrosomes are membraneless spherical organelles consisting of a pair of centrioles at the center (Fig 4A), surrounded by a porous scaffold-like structure [34] called the pericentriolic matter (PCM). In cells preparing to enter mitosis, the two centrosomes are spatially separated and grow by recruiting PCM material around the centrioles [8, 35–38]. While the mechanics of PCM assembly is a subject of ongoing debate [39], the molecular components for PCM growth must ensure robust size control of centrosomes during mitosis. Otherwise, small stochastic variations in the sizes of maturing centrosomes could amplify through the process of maturation, leading to large difference in the two growing centrosome size. Furthermore, experimental data show that centrosome size scales with cell size, through multiple rounds of cell divisions in the early *C. elegans* embryo, suggesting that centrosome size is determined by a limiting pool of building blocks [1, 8]. Since the limiting pool model cannot maintain the size of two structures competing for the same subunit pool (Fig 1), additional feedback controls must be necessary for centrosome size regulation.

While the assembly of PCM is coordinated by many different proteins, Centrosomin (Cnn) and Spindle defective-2 (Spd-2, or Dspd-2 in *Drosophila*) are the most essential components of PCM, in the absence of which PCM assembly does not occur [38]. Furthermore, increased amount of Cnn and Spd-2 leads to the formation of larger mitotic centrosomes [35, 40]. It is reported that both these proteins assemble very close to the centriole and then migrate outwards during PCM scaffold formation and growth [38, 41]. Thus the assembly rate of PCM will depend on the centriole size that remains constant during PCM assembly, resulting in a constant assembly rate *k*^+^ that is centrosome size-independent but centriole size-dependent. Our consideration is consistent with experimental data reporting smaller (damaged) centrioles forming smaller centrosomes during PCM assembly [42].

The PCM scaffold is an amorphous and porous structure [35], where the disassembly of the scaffold proteins and other associated PCM proteins occurs throughout the PCM volume. The disassembly rate can thus be assumed to be proportional to the centrosome volume, given by *k*^−^*n*, where *n* is the number of PCM building blocks incorporated in the centrosome and *k*^−^ is the bare disassembly rate. Based on these considerations, we propose a simple kinetic model for the growth of centrosomes that assemble PCM building blocks from a limiting cytoplasmic pool. PCM assembly is localized around the centriole, and disassembly can occur throughout the volume of the porous PCM giving rise to a size-dependent disassembly rate (Fig 4A). A deterministic description for this growth process is given by,

$$\begin{array}{c} \dot{n}=k^{+}\frac{\left(N-n\right)}{V}-k^{-}n\;, \end{array}$$ (8)

where *V* is the volume of the cell, and *N* is the total amount of the PCM building blocks that can be estimated from proteomics data. Knowing *V* and *N* from independent measurements, the rate constants *k*^+^ and *k*^−^ can be determined by fitting experimental data for the steady-state size of the centrosome *k*^+^*N*/(*k*^+^ + *k*^−^*V*), and the timescale for reaching the steady-state (*k*^+^/*V* + *k*^−^)^−1^. The growth model in Eq (8) directly maps to our general model for size-dependent growth with (*α*, *β*) = (0, 1) (Fig 2). Since *α* + *β* > 0, the model ensures robust size control of two centrosomes assembled from a shared resource pool.

We use the stochastic description of (8) to demonstrate size control of a pair of centrosomes growing to be of the same size in a limiting pool of the subunits (Fig 4B). Aside from ensuring size control, our model for centrosome assembly can quantitatively capture the scaling of centrosome size with centrosome number (Fig 4C) and cell volume (Fig 4D), as measured during early *C. elegans* embryonic development [8]. Our simplified growth description does not capture the sigmoidal nature of growth thus it may be more applicable to *Drosophila* centrosome growth rather than *C. elegans* [9].

#### Liquid-liquid phase separation model

Recent studies have suggested the role of liquid-liquid phase segregation (LLPS) in centrosome maturation and growth control [9], but the role of LLPS in centrosome size control remains a highly debated issue [39]. Here we discuss centrosome growth using the LLPS model developed by Zwicker *et al* [9], to show how it compares to our kinetic self-assembly model. We specifically discuss PCM droplet growth in the limit where phase segregation is strong and growth is limited by chemical reaction (i.e., fast diffusion of PCM components).

The growth of the centrosome, considered as a liquid droplet of volume *V*, is given by

$$\begin{array}{c} \frac{dV}{dt}=\left(k\phi_{1}^{A}-k_{BA}\right)V+Q\frac{\phi_{1}^{A}}{\psi_{-}}+k_{AB}\frac{\phi_{0}^{A}V_{c}}{N\psi_{-}} \end{array}$$ (9)

where *ϕ*^*A*^ and *ϕ*^*B*^ are the volume fractions for the soluble (*A*) and the phase segregated (*B*) forms of the PCM components, respectively, and *ψ*_−_ is the volume fraction of *B* inside the droplet. Here *k*_*AB*_, *k*_*BA*_ and *k* are the reactions rates for *A* → *B*, *B* → *A* and *AB* → *B*, respectively. The bulk volume fraction of the *A* and *B* forms, away from the droplet, is given by $\phi_{0}^{A}$ and $\phi_{0}^{B}$ respectively and $\phi_{1}^{A}$ is the volume fraction of *A* inside the droplet. The chemical activity of the centriole, volume of the cell, and the number of centrioles growing are given by *Q*, *V*_*c*_ and *N*, respectively.

We neglect spontaneous production of phase segregated form *B* away from the centriole (*k*_*AB*_ = 0), and the volume fraction of *B* inside the droplet is considered to be unchanged, i.e., *ψ*_−_= constant. The volume fractions of *A* in the bulk and inside the droplet are given by

$$\begin{array}{c} \phi_{0}^{A}=\bar{\phi }-\psi_{-}(\frac{NV}{V_{c}}) \end{array}$$ (10)

$$\begin{array}{c} \phi_{1}^{A}=\left(1-\psi_{-}\right)\phi_{0}^{A} \end{array}$$ (11)

where $\bar{\phi }$ is the average volume fraction of the total PCM material. We define $\bar{\phi }=\frac{V_{A}+V_{B}}{V_{c}}=\frac{N_{\text{tot}}}{V_{c}}$, where *N*_tot_ is the total amount of PCM material that remains constant during the droplet growth. We also write $\psi_{-}=\frac{n_{B}\delta_{v}}{V}$, where *n*_*B*_ and *δ*_*v*_ are the number of *B* subunits inside droplet and characteristic volume of each subunit respectively. Now we can rewrite $\phi_{0}^{A}=\frac{N_{av}\delta_{v}}{V_{c}}$ and $\phi_{1}^{A}=\left(1-\psi_{-}\right)(\frac{N_{av}\delta_{v}}{V_{c}})$ where *N*_*av*_ = *N*_tot_ − *n*_*B*_ is the available amount of subunits that can contribute to the droplet growth. Finally using the definition of *ψ*_−_ we get the *n*_*B*_ dynamics given by

$$\begin{array}{c} (\frac{\delta_{v}}{\psi_{-}})\frac{dn_{B}}{dt}=\frac{dV}{dt} \end{array}$$ (12)

and this simplifies to

$$\begin{array}{c} \frac{dn_{B}}{dt}=\left(1-\psi_{-}\right)(kn_{B}+\bar{Q})(\frac{N_{av}\delta_{v}}{V_{c}})-k_{BA}n_{B} \end{array}$$ (13)

where $\bar{Q}=\frac{Q}{\delta_{v}}$. This above description then can be rewritten as

$$\begin{array}{c} {\dot{n}}_{B}=\left(C_{0}+C_{1}n_{B}\right)(\frac{N_{av}}{V_{c}})-C_{2}n_{B} \end{array}$$ (14)

where *C*_0_ = (1 − *ψ*_−_)*Q*, *C*_1_ = (1 − *ψ*_−_)*kδ*_*v*_ and *C*_2_ = *k*_*BA*_. In the case of *C*_1_ ∼ *C*_0_ (closer to passive phase separation limit), this growth description maps to our size dependent growth model with *α* = −1, *β* = 1. Thus, this growth mechanism will lie on the limiting pool line, *α* + *β* = 0, which does not guarantee size control for multiple structures. In the limit *C*_0_ ≫ *C*_1_, where the phase segregation is strongly controlled by centriole chemical activity (*Q*), the LLPS model results are very similar to our localized assembly model, and is able to provide robust size control for multiple centrosomes. Our theory assumes a strong regulation of centrosome growth by the size and chemical activity of centriole. Thus our theory naturally predict that irregularities in centrosome size regulation may arise from errors in centriole assembly. This can be important in pathological scenarios like cancer where centrosome irregularities has been observed [43].

### Nucleus size control: A case for coupled growth of filaments and an organelle

Nucleus is a highly complex organelle, but in a very simplified manner it can be considered to be composed of two key components—the inner nucleoplasm (NP), surrounded by the outer nuclear envelope (NE). During nucleus growth, both the NP and NE components grow from their respective pool of building blocks. However, which one of these two components controls nucleus size (i.e., nuclear radius) and growth remains an open question [44]. Though there are many studies reporting the scaling of nucleus size with cell size [45–47], it is not well understood how nucleus size is regulated. To this end, we present a simple two-component model for nucleus—an outer spherical shell representing NE, and an interior spherical body representing the NP. We demonstrate how the geometric design of NE and NP assembly leads to nuclear size regulation, and compare our model predictions with available experimental data.

#### Growth of nucleus by nuclear envelope assembly

**Fig 5:**
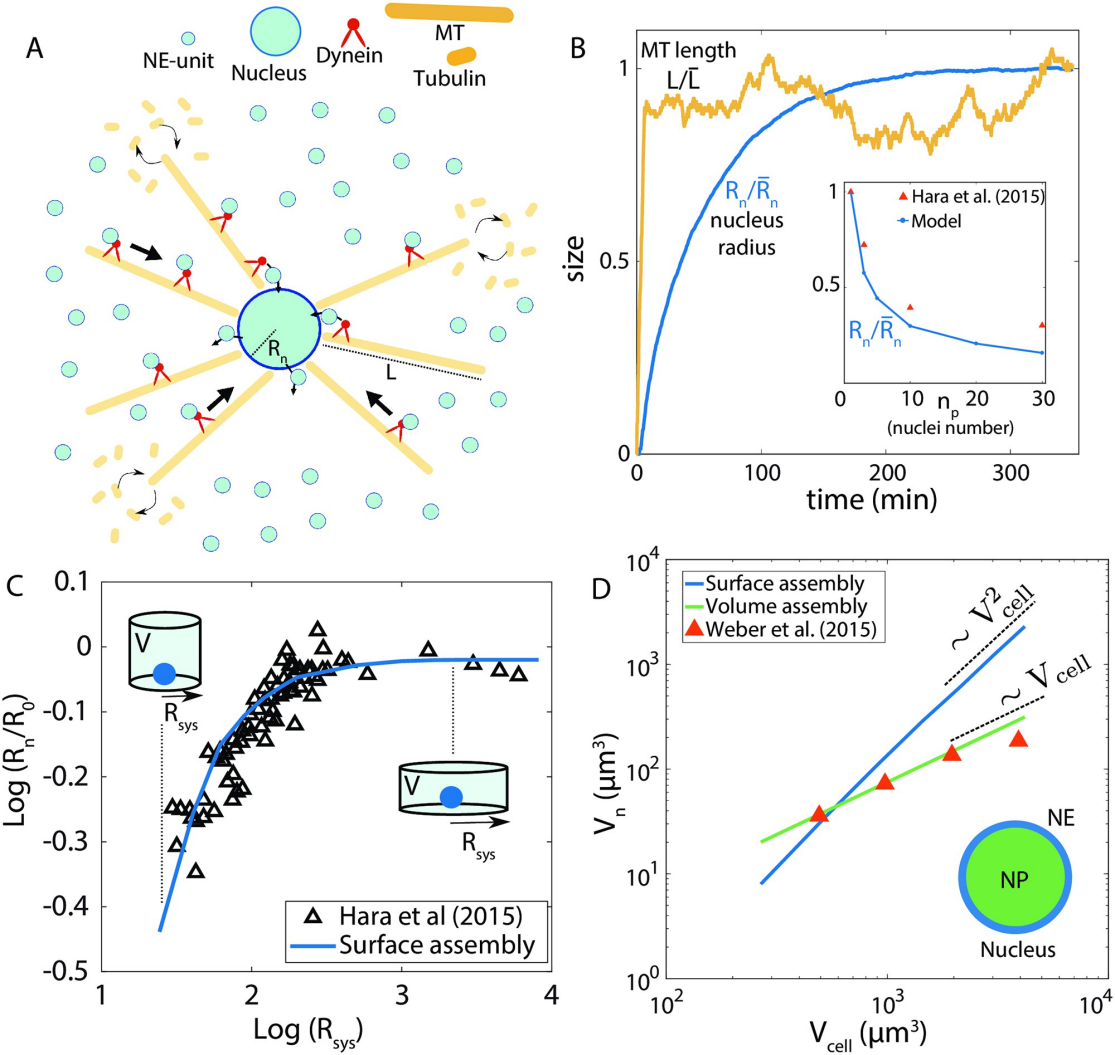
Growth of nucleus surface and volume assembly. (A) Modelling the growth of nucleus, coupled to the dynamics of the astral microtubule structure. Building blocks for nuclear envelope (NE) are actively transported by dynein motors along the astral microtubules surrounding the nucleus. Filaments in the aster grow by incorporating tubulins from the cytoplasmic pool. (B) Dynamics of nucleus size (normalised radius *R*_*n*_) and single microtubule length (normalised). (Inset) Nucleus size decreases with increasing nuclei number in a given volume, in agreement with *in vitro* data [48]. (C) Effect of the size of confinement, *R*_sys_, on nucleus size *R*_*n*_, where *R*_*n*_ increases with increasing *R*_sys_, eventually saturating for large *R*_sys_. Solid line is model fit, and black triangles represent experimental data [48]. The confinement radius was increased while keeping the confinement volume constant, as in experiments [48]. (D) Scaling of nucleus volume, *V*_*n*_, with cell volume *V*_cell_. Theory predicts that size scaling is quadratic if *V*_*n*_ is controlled by the growth of NE, but the scaling is linear if *V*_*n*_ is regulated by NP assembly. The linear scaling fits quantitatively with the nucleus-to-cell size scaling measured in an eukaryotic cell [10]. Parameters: NE subunit concentration = 8.0 *μ*m^−3^, *α* = 0, *β* = 1, NE subunit size (*δA*) = 0.215 *μ*m^2^, *k*^+^(NE) = 5 × 10^−3^ *μ*m^3^min^−1^, *k*^−^(NE) = 10^−1^min^−1^, NP subunit concentration = 0.75 *μ*m^−3^, NP subunit size (*δV*) = 0.1 *μ*m^3^, *k*^+^(NP) = 2.0 *μ*m^3^min^−1^, *k*^−^(NP) = 10^−3^ min^−1^, MT subunit concentration ≃ 0.67 *μ*M, MT subunit size (*δL*) = 5 nm, $k_{m}^{+}=2.0$ *μ*m^3^min^−1^, $k_{m}^{-}=10^{-2}$ min^−1^.

We first consider a model for nucleus growth by nuclear envelope (NE) assembly, taking inspiration from a recent *in vitro* study in *Xenopus levis* egg extract [48], where nucleus growth is coupled to the growth of microtubule asters surrounding the nucleus [48–50]. Here, NE assembly occurs through active incorporation of nuclear membrane vesicles/fragments (building blocks of NE) by dynein motors moving along astral microtubule tracks (Fig 5A). The microtubule aster is a collection of many dynamic microtubules surrounding the nucleus, where each individual filament grows from a cytoplasmic pool of tubulins (building blocks of microtubules) (Fig 5A). The rate of NE assembly is proportional to the size of the microtubule aster, as the number of available NE building blocks scales with the volume spanned by the the aster. As the NE grows in size maintaining a constant thickness, we assume that the NP volume expands accordingly to accommodate the increase in nuclear surface area. The deterministic rate equations for the growth of one nucleus is given by

$$\begin{array}{c} \dot{n}=K^{+}\left(\bar{L},R_{n}\right)(\frac{N-n}{V})-k^{-}n\;, \end{array}$$ (15)

$$\begin{array}{c} \dot{L_{i}}=k_{m}^{+}(\frac{N_{m}-\sum_{i}L_{i}}{V})-k_{m}^{-}L_{i}\;, \end{array}$$ (16)

where *R*_*n*_ is the nucleus radius, *n* and *L*_*i*_ are the sizes of NE (in building block units) and the *i*^*th*^ microtubule filament, respectively. The total amount of tubulin and NE building blocks are given by *N*_*m*_ and *N*, respectively, and *V* is the cell (or system) volume. Here *k*^±^ and $k_{m}^{\pm }$ are the bare rates of assembly and disassembly for NE and microtubules, respectively.

Since assembly occurs at the surface, size of the nucleus is determined by the relation $4\pi R_{n}^{2}=n\delta_{A}$, as *n* building blocks, each of area *δ*_*A*_, make up the NE. The size-dependent assembly rate is given by $K^{+}\left(\bar{L},R_{n}\right)=k^{+}\left(4\pi /3\right)({\left(\bar{L}+R_{n}\right)}^{3}-R_{n}^{3})$, proportional to the volume accessible to the aster structure, and $\bar{L}$ is the average length of the microtubules. Initially, the assembly rate induces a positive feedback on NE growth as $\bar{L}\ll R_{n}$. As assembly progresses, the filaments become longer to yield $\bar{L}\gg R_{n}$, such that *K*^+^ becomes independent of *R*_*n*_. Disassembly occurs uniformly throughout the NE surface, yielding a size-dependent disassembly rate, *k*^−^*n*, which provides a negative feedback on NE growth. The later stages of NE growth can thus be mapped to our general growth model with (*α*, *β*) = (0, 1) (Fig 2 and S6 Text), ensuring robust size control. Length dynamics of microtubules is implemented using the antenna model [22, 23], where the filament disassembly rate increases linearly with filament length (see S5 Text).

We simulated the stochastic dynamics of nucleus growth using the model in (15) and (16), with the model parameters calibrated from *in vitro* data [48]. Both the filaments and the nuclear envelope reach a well-defined steady-state mean size (Fig 5B). Initially, NE size increases rapidly due to size-dependent assembly rate (*K*^+^), whereas growth slows down at later times when *K*^+^ balances the rate of NE disassembly (Fig 5B). Our model quantitatively captures the experimentally observed scaling between nucleus size and nuclei number, when multiple nuclei are assembled from a limiting pool of subunits (Fig 5B, inset). The nuclear size, *R*_*n*_, decreases with increasing nuclei number, as expected from a limiting pool of building blocks. This coupled growth model of NE and microtubules can also explain the nucleus size dependence on the size of confinement as reported in *in vitro* experiments [48]. Here, the microtubule growth is hindered by the confinement wall, and hence a smaller system size will generate a smaller aster structure. As a result, the assembly rate will be smaller giving rise to a smaller nuclear size (Fig 5C). However, the maximum nucleus size is set by the steady-state length ($\bar{L}$) of the microtubule filaments (when all other conditions remain unchanged). Therefore, increasing the confinement size larger than $\bar{L}$ does not generate a larger nucleus (Fig 5C). This can explain why an isolated nucleus grows to be larger in size than a group of closely packed nuclei in multinucleated cells. This is because the presence of neighbouring nuclei hinder microtubule growth [50], resulting in reduced nuclear size. A recent study on sea urchin nucleus growth suggests that perinuclear endoplasmic reticulum may act as a limiting resource pool for the NE and nucleus size scaling is controlled by scaling of perinuclear endoplasmic reticulum volume during embryonic development [51]. Such results indicate the importance of considering the nuclear envelope growth to understand nuclear size regulation.

#### Growth of nucleus by nucleoplasm assembly

During nuclear growth in *Xenopus levis* egg extract, many NP proteins such as lamin-A, importin-*α* [11] are transported inside the nucleus and contribute to NP growth [52, 53]. The nucleoplasm being very complex in structure and composition, the NP proteins do not necessarily assemble into a bigger structure to span the necleoplasm rather, in this context, by assembly we mean that the NP proteins contribute to nucleoplasm volume. To model growth of nucleus by NP assembly, we use the rate equations in (15) and (16), with the important difference that the size of nucleus is determined by the relation: $V_{n}=\frac{4\pi }{3}R_{n}^{3}=n\delta_{V}$, where *V*_*n*_ is the nucleus volume and *δ*_*V*_ is volume of individual NP subunits. Thus, when NP regulates nucleus size, *V*_*n*_ is proportional to the number of NP subunits incorporated, *n*. This model for nucleus growth by NP assembly does not alter the effect of confinement, but predicts the scaling relation, $R_{n}∼n^{\frac{1}{3}}$. This scaling is different when nucleus size is regulated by NE assembly, where, $R_{n}∼n^{\frac{1}{2}}$ (Fig 5D). This difference can be understood by relating NE-growth and NP-growth with growth of a spherical shell of constant thickness and the growth of a solid sphere, respectively. By comparing our simulation results with experimental data [10, 46], we find that the experimentally observed linear scaling of nuclear size with cell size cannot be achieved when nucleus size is purely regulated by NE assembly (Fig 5D), but NP-growth model leads to *V*_*n*_ ∝ *V*_cell_. While our simplified description can capture experimentally reported nuclear size scaling and the effect of confinement on nuclear growth, the question of whether NP or NE regulate nucleus size and growth cannot be answered without considering the diverse modes of nuclear envelope remodeling [54, 55]. Interestingly, a recent study on spindle size regulation shows that both area dependent and volume dependent regulation of size can co-exist to give rise to a non-linear scaling behaviour [7]. Our modeling framework provides a possible way to test the different underlying mechanisms of nuclear growth and size control. To keep our model description simple we have not considered the nuclear pore complexes (NPCs) which plays a vital role in regulating nuclear import [56], but NPC density and activity can be conceptualized as the factor controlling the *K*^+^ parameter in our model.

### Bistable size regulation from autocatalytic growth

**Fig 6:**
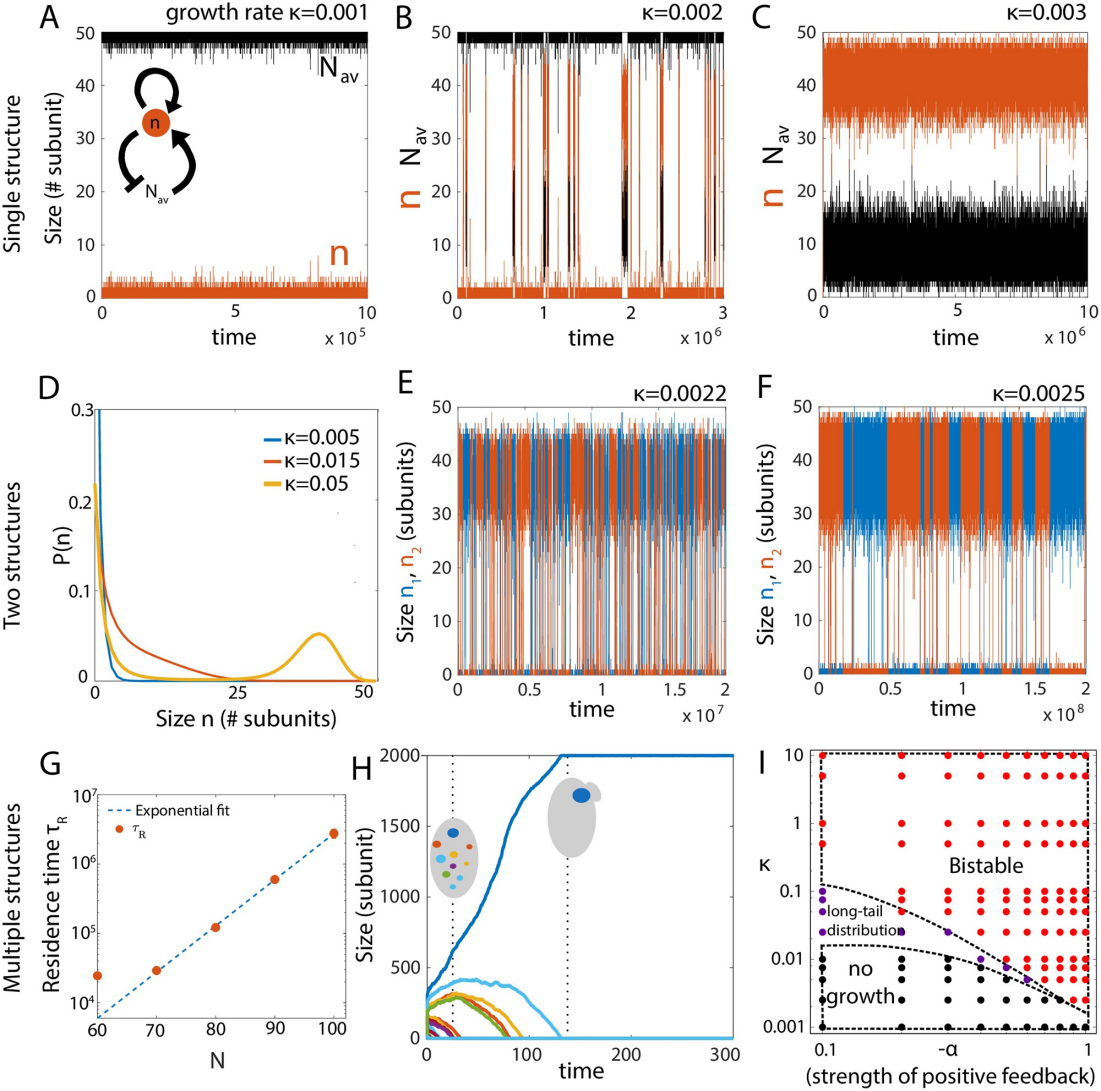
Bistable size distribution emerges from autocatalytic growth. Bistability in a single structure growth occurs in a range of *κ* value. (A) The structure does not grow substantially for very small growth rates (*κ* < κ*) but (B) it shows bistability and bimodal size distribution in an intermediate range of growth rates. (C) For growth rates higher than a critical value *κ* > *κ*^*c*^, the structure takes up almost the whole pool and grows to be very large as expected in autocatalytic growth. Parameters for A-C: *N* = 50, *α* = −1, *β* = 0. (D) Probability distribution for the size of two identical structures (*κ*_1_ = *κ*_2_ = *κ* = *k*^+^/*k*^−^) assembled via autocatalytic growth. The structures do not grow at very low growth rates, leading to an exponential distribution peaked at 0. At high *κ*, the size distribution is bistable. Parameters: *N* = 50, *α* = −0.2, *β* = 0. (E-F) Dynamics of the size of the two structures in the bistable regime at two different growth rates *κ* = 0.0022 (E) and *κ* = 0.0025 (F). Parameters: *N* = 50, *α* = −1.0, *β* = 0. (G) Residence time (*τ*_*R*_) of a single structure increases exponentially with increasing concentration of subunits. Dashed line—exponential fit, solid circles—simulation data. (H) Stochastic selection of a single structure when multiple structures compete for a limiting subunit pool, for *α* + *β* < 0 and high subunit concentration. Here, the residence time of a single structure becomes seemingly infinite, so the stochastically chosen large structure can remain stable for long timescales. Parameters: *N* = 2000, *κ* = 0.0125, *α* = −1, *β* = 0, and number of structures = 20. (I) State diagram of the system, showing different growth regimes as a function of *κ* and the strength of positive feedback, −*α* (with *β* = 0). By increasing *κ* at any non-zero value of −*α*, the system transitions from a “no-growth” state (black dots), to a “shoulder” state (purple dots) at intermediate *κ*, and finally a bistable state (red dots) for high *κ*. At very high *κ*, *τ*_*R*_ can be very large to effectively give rise to a single-structure. Increasing the strength of positive feedback promotes a bistable state at smaller *κ*. Parameters: *β* = 0, *V* = 1 and *N* = 50.

So far we focused on robust size control of multiple structures via size-dependent negative feedback on growth (i.e., *α* + *β* > 0). In the opposite case of size-dependent positive feedback, *α* + *β* < 0, the dynamics are qualitatively different. For a single structure, positive feedback on growth implies an assembly (disassembly) rate that increases (decreases) with increasing structure size, resulting in autocatalytic growth. When the effective growth rate is small *κ* < *κ*_0_, where *κ*_0_ is a threshold, the structure fails to grow significantly and show large size fluctuations compared to the mean size (Fig 6A). The size dynamics is bistable and gives rise to a bimodal size distribution in an intermediate range of growth rate (Fig 5B and S4 Fig). At a growth rate higher than a critical value *κ*^*c*^ (*κ* > *κ*^*c*^) the size dynamics becomes monostable and the single structure grows to be large, by depleting almost the entire subunit pool (Fig 6C and S4 Fig). Thus, bistable size regulation of a single structure is observed in a range of growth rate (κ* < *κ* < *κ*^*c*^). See S2 Text for further analysis of autocatalytic growth of a single structure from a limiting subunit pool.

The result becomes less intuitive when there are two identical structures (with equal assembly/disassembly rates) competing for a shared subunit pool. When the growth rate is small, the structures do not grow significantly and attain an almost exponential size distribution (Fig 6D, blue) with large fluctuations compared to the mean size (S6 Fig). With sufficiently large growth rate, the structures initially grow at equal rates, but due to transient size differences arising from stochastic fluctuations, the bigger structure starts assembling faster than the smaller structure and ends up incorporating most of the building blocks. However, at this stage, stochastic fluctuations can make the larger structure lose enough building blocks to make a sudden transition to a smaller structure, while the other structure grows to be larger (Fig 6E and 6F). Thus, above a critical growth rate, we get bistable size dynamics and a bimodal size distribution of two structures competing for a shared pool (Fig 6D, yellow). There is an intermediate regime where the structures show large size fluctuations without bistable dynamics, and the size distribution exhibits a “shoulder” and a longer tail (Fig 6D, red).

To characterize the kinetics of state transitions between the two steady states of the structure, we compute the residence time *τ*_*R*_, which is the average duration the structure is found in one of the steady states. We find that *τ*_*R*_ increases with the growth rate (Fig 6E and 6F), as well as increases exponentially with the total pool size *N* (Fig 6G). This phenomenon of dynamic switching between a small and a large size is a consequence of stochastic growth and cannot be understood from a purely deterministic description. The statistics of state transitions, characterized by *τ*_*R*_, depends on the individual values of *α* and *β* (S4 Fig). In the case of multiple structures (*M* > 2) the size dynamics remain qualitatively similar with dynamic transitions among states characterized by a larger size of one of the structures and a small size for all other structures (S11 Fig).

Bistable size distribution has been reported in microtubule/kinesin-8 *in-vitro* systems [57], where kinesin-8 motors bind to the microtubules, walk towards the plus end and disassociate from the filament by removing a tubulin dimer [17, 22, 23]. This active disassembly depends on the motor concentration profile, which in turn depends on the filament length [18]. This can lead to a reduced concentration of motors at the tip of a longer filament, generating a positive feedback which can be approximately mapped to our minimal model with *α* = −2 and *β* = 1, (Fig 2 yellow-black square, S7 Text) and bimodal length distribution (S5 Fig).

In the limit of high subunit concentration, a stochastically selected larger structure will consume all the building blocks to increase in size, but stochastic fluctuations may take a very long time to make the structures switch states. Depending on the strength of the positive feedback, growth rates and building block concentration, residence time of the bigger structure can be so large that the transition to a smaller structure may not occur within a realistic timescale. Thus an autocatalytic growth process (*α* + *β* < 0) can be used to regulate the formation of a single structure starting from noisy initial state containing many structures. This phenomenon can be related to the process of polarity establishment in budding yeast, where the budding mechanism requires the formation of one single concentrated patch of the polarity protein Cdc42 that marks the budding location to initiate the subsequent process of budding [58]. Many studies have linked positive feedback with the process of Cdc42 patch formation via various physical mechanisms [59, 60] but the mechanism of polarity formation is not fully understood. Our model for autocatalytic growth of structures from size-dependent positive feedback is in good agreement with the previously stated mechanism of polarity establishment. In our case if we start with many structures only a few can survive to gain considerable size, but finally the stochastically chosen largest structure will win to form a single large structure while the other structures eventually die out (Fig 6H).

Interestingly, Cdc42 proteins also exhibit recurring polarity patch formation (flickering) during polarity establishment in budding yeast [60, 61] and oscillations during growth in fission yeasts [62]. Our model predicts that at an intermediate concentration of building blocks, multiple growing structures enter a dynamic state (S12 Fig), where the residence time is small enough to promote transitions between large- and small-sized structure within experimentally relevant timescales. This is in good qualitative agreement with recent studies of Cdc42 polarity establishment [60, 61], where a decrease in overall protein amount or feedback strength promotes flickering recurrent polarity patches, generating many transient structures instead of one single large structure. The negative feedback needed for these dynamic transitions stems from the limited amount of the subunit pool. With increasing strength of positive feedback, bistability can occur at smaller growth rates (Fig 6I). Furthermore, an increase in system size (i.e., increase in total pool size as the total subunit concentration is kept constant) can drive a transition from multiple coexisting structures to the growth of a single structure (S10 Fig). This size-dependent transition is a feature of stochastic dynamics, and can coordinate size-dependent symmetry breaking and pattern formation in developmental systems that follow similar feedback motifs [63].

### Constant subunit concentration

**Fig 7:**
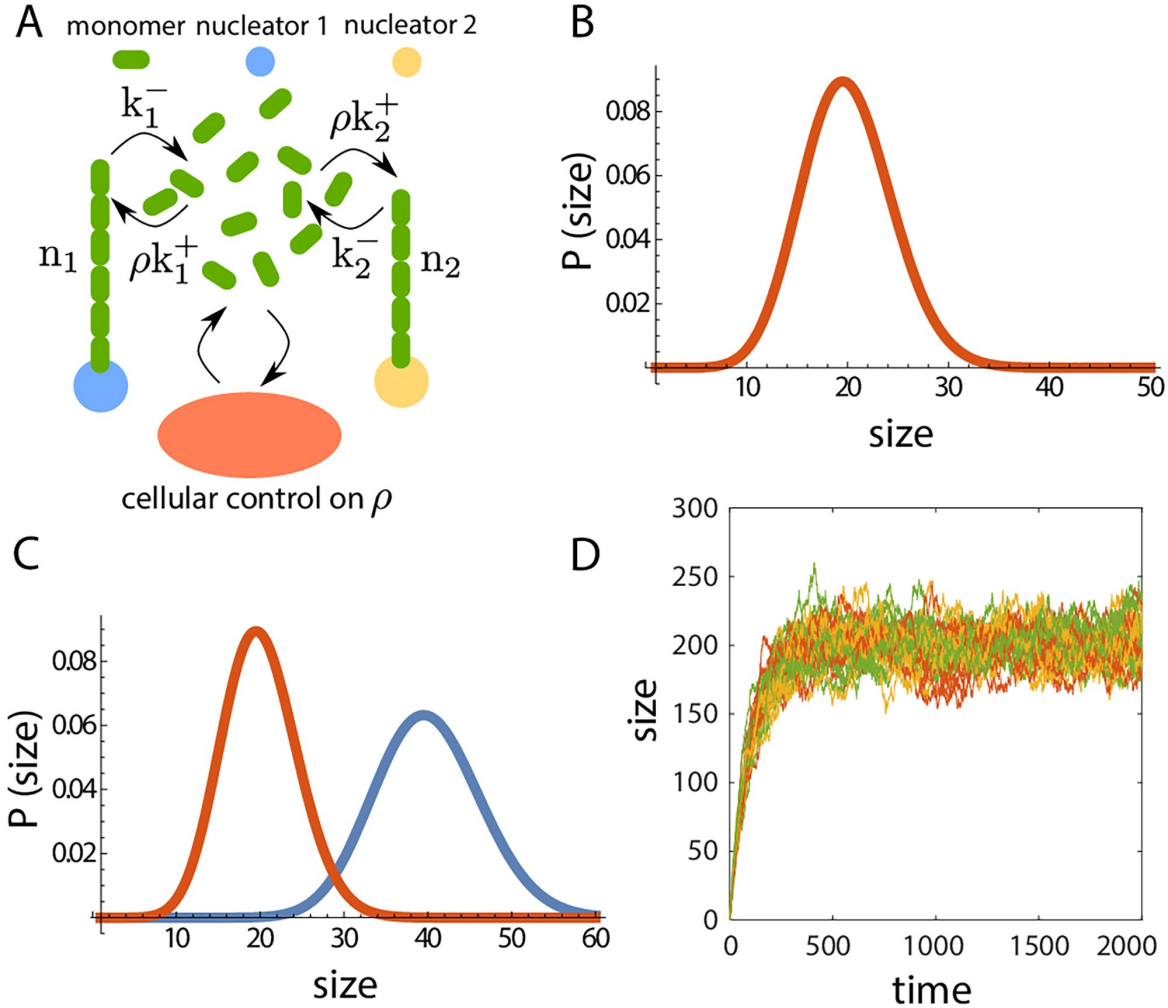
Growth of structures with constant subunit concentration. (A) Schematic representation of the growth of two structures, where the cell maintains a fixed cytoplasmic density of subunits. (B-C) Size-dependent negative feedback enables robust size control of two structures with (C) or without (B) inherent difference in bare growth rates. The steady-state size distributions for the two structures in (B) and (C), computed analytically by solving the master equation. (D) The constant subunit concentration model does not lead to any scaling between the structure size and the system size, when multiple structures growing from a shared subunit pool. The plot shows time series for the size of 10 (green), 20 (yellow) and 40 (red) structures grown from a pool of concentration *ρ* = 2, with *κ* = 100.

Next we ask how cells regulate the size of intracellular structures when the subunit pool is not limited, but is maintained a constant homeostatic concentration. When the cell maintains a constant subunit concentration, *ρ*, the deterministic growth dynamics for *M* number of structures growing from a shared pool can be written as

$$\begin{array}{c} \dot{n_{i}}=k_{i}^{+}\rho -k_{i}^{-} \end{array}$$ (17)

where $k_{i}^{\pm }$ is the assembly and disassembly rates for *i*^*th*^ structure and *n*_*i*_ is the size of the *i*^*th*^ structure in number of subunits. The structures will keep growing without bound when $\dot{n_{i}}>0$. This unbounded growth occurs above a critical subunit density *ρ*_*c*_ given by $\dot{n_{i}}=0$ -i.e., $\rho_{c}=k_{i}^{-}/k_{i}^{+}$. Below this critical density the structures do not grow at all. Recovery from this failure in size control occurs when the growth process has size-dependent negative feedback

$$\begin{array}{c} \dot{n_{i}}=k_{i}^{+}\rho {\left(1+n_{i}\right)}^{-\alpha }-k_{i}^{-}n_{i}^{\beta } \end{array}$$ (18)

where *α* + *β* > 0. This growth mechanism is distinct from the limiting pool model in a manner that results in structures growing independently of each other and features such as bistability of size cannot be seen. To explore the features of such growth, we study the stochastic dynamics of two structures growing from a shared subunit pool that is maintained at a fixed concentration (Fig 7A). The resulting master equation can be analytically solved to find the marginals given by

$$\begin{array}{ccc} P(n_{1,2}) & = & \sum_{n_{1,2}=0}^{\infty }(\frac{\kappa_{1}^{n_{1}}\kappa_{2}^{n_{2}}\rho^{n_{1}+n_{2}}}{{\left(n_{1}!\right)}^{\alpha +\beta }{\left(n_{2}!\right)}^{\alpha +\beta }})P\left(0,0\right), \end{array}$$ (19)

where *P*(*n*_*i*_) is the probability of finding the *i*^*th*^ structre in size *n*_*i*_ and *P*(0, 0) is the probability of finding both the structures at zero size (see S8 Text for details). We compute this sum numerically to calculate the discrete size distributions. We can easily see that taking *α* = 0, *β* = 0 we get a probability distribution that is not normalizable, signifying unbounded growth and the non-existence of a steady-state size distribution, unless *ρ* is smaller than the critical subunit density $\rho_{c}=k_{i}^{-}/k_{i}^{+}$, giving rise to non-growing structures. With *α* + *β* > 0 we obtain size control of multiple structures with or without inherent differences in bare growth rates (Fig 7B and 7C). While individual size control is achieved, this growth mechanism does not provide access to information on cell/system size. Thus it is not possible to get structure size scaling with cell size or structure number, when subunit concentration is maintained at a constant value (Fig 7D).

### Structure size scaling

Size of intracellular structures or organelles often scale with cell size [8, 46, 47] for proper physiological functionality, but it may also be desirable for organelle sizes not to scale with cell size. Our proposed model for structure growth control by size-dependent negative feedback, enables us to tune the structure-to-cell size scaling. To illustrate this, we take the examples of *α* = 0, *β* = 1 (disassembly rate increasing with structure size) and *α* = 1, *β* = 0 (assembly rate decreasing with structure size). We further assume that all structures grow with the same bare assembly and disassembly rates, i.e., $k_{i}^{\pm }=k^{\pm }$, which leads to identical steady-state sizes (n*) for all the *M* number of growing structures, such that ∑_*i*_ *n*_*i*_ = Mn*. For *α* = 0, *β* = 1 the deterministic rate equations are given by,

$$\begin{array}{c} {\dot{n}}_{i}=k^{+}(\frac{N-\sum_{i=1}^{M}n_{i}}{V})-k^{-}n_{i}\;, \end{array}$$ (20)

which leads to the steady-state solution,

$$\begin{array}{c} n^{*}=\frac{\kappa N}{\kappa M+V}\;, \end{array}$$ (21)

where *κ* = *k*^+^/*k*^−^, *V* is the cell volume and *M* is the number of structures. The overall subunit density, *ρ*_0_ = *N*/*V*, is a constant independent of cell size *V* and total pool size *N*. We can thus rewrite the steady-state size as,

$$\begin{array}{c} n^{*}=\frac{\kappa \rho_{0}V}{\kappa M+V}. \end{array}$$ (22)

**Fig 8:**
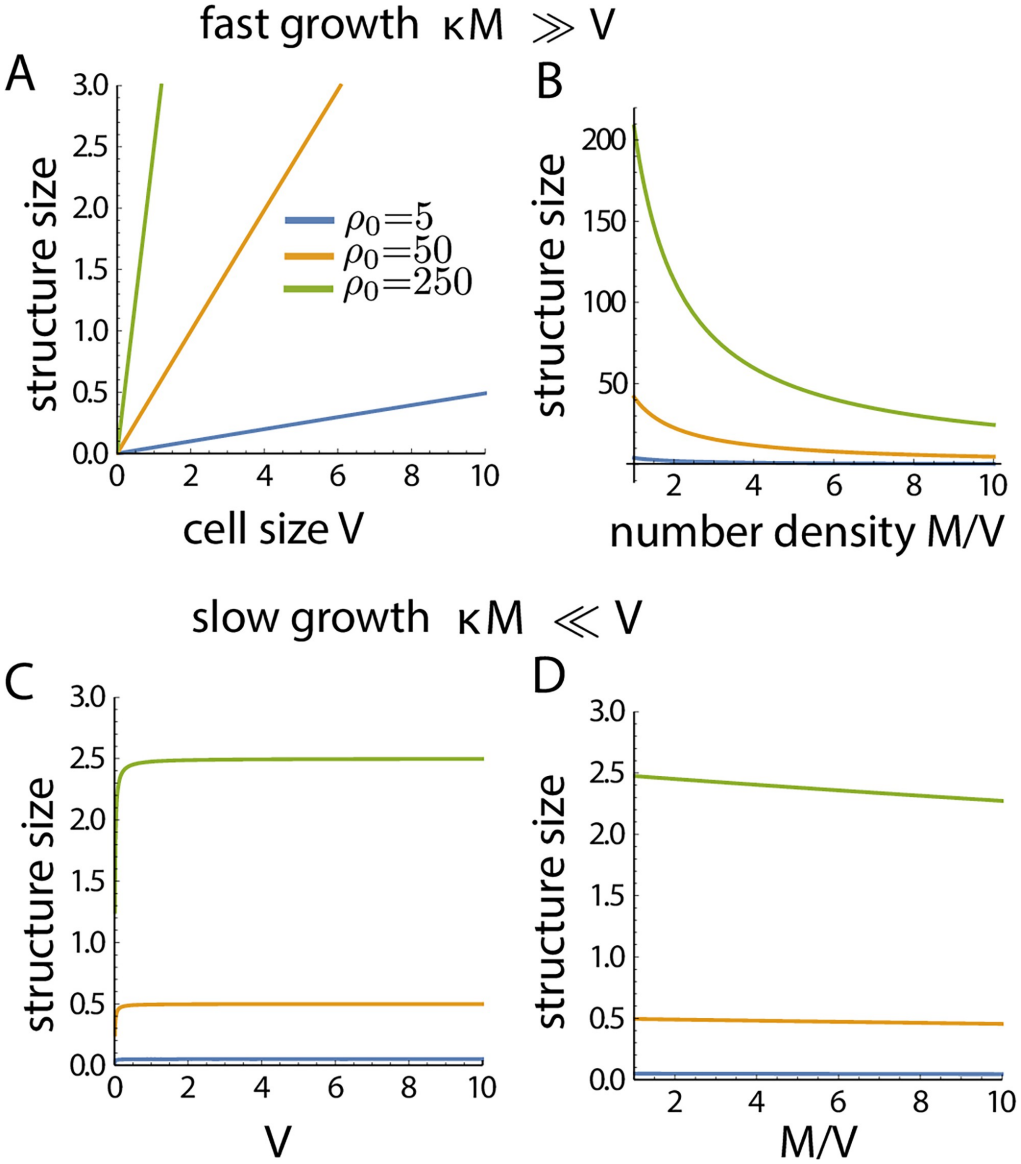
Structure size scaling with system size. The size-dependent growth model (with *α* = 0, *β* = 1) exhibits a regime where structure size does not scale with cell/system size (*V*) or the number of assembled structures, *M*. Growth rate of the structures can be tuned to achieve scaling of structure size with cell size. (A-B) shows the regime of structure size scaling with cell size, whereas (C-D) shows the regime no size scaling.

In the limit *κM* ≫ *V*, the structure size scales linearly with cell size as n* ∼ *ρ*_0_*V*/*M* (Fig 8A), and scales inversely with number of organelles assembled (Fig 8B). By contrast, when *κM* ≪ *V* then n* ∼ *κρ*_0_, i.e., the structure size is independent of cell size and M (Fig 8C and 8D). For *α* = 1, *β* = 0 the deterministic rate equations are,

$$\begin{array}{c} {\dot{n}}_{i}=k^{+}(\frac{N-\sum_{i=1}^{M}n_{i}}{V}){\left(1+n_{i}\right)}^{-1}-k^{-}\;, \end{array}$$ (23)

which leads to the steady-state solution

$$\begin{array}{c} n^{*}=\frac{(\kappa \rho_{0}-1)V}{\kappa M+V}. \end{array}$$ (24)

As before, in the limit *κM* ≫ *V* we get linear scaling of structure size with cell size, $n^{*}∼\frac{(\kappa \rho_{0}-1)V}{\kappa M}$, and inverse scaling with the number of organelles. However, in the limit *κM* ≪ *V* we get n* ∼ *κρ*_0_ − 1, such that the structure size is independent of cell size and the number of organelles assembled. These results where the structure size scaling becomes weak or sub-linear with cell size has been observed for nuclear size scaling at higher cell volume [10, 51, 64].

### Effect of cellular growth on structure size control

So far we have considered the system size or the cell size to remain unchanged during structure growth. However in reality cells can grow in size, divide and undergo various morphological changes while different intracellular structures are growing. Here we discuss the effect of cellular growth on the size control of intracellular structures. We assume that a cell starts growing with an initial subunit abundance *N*_0_ and volume *V*_0_. Depending on the cell type, single-cell growth can be described using a linear or an exponential growth law [65]. Here for simplicity, we assume a linearly growing cell modeled by the following equations:

$$\begin{array}{ccc} \dot{V} & = & g\;\delta V\;,\\ \dot{N} & = & g\;, \end{array}$$ (25)

where *g* is the growth rate and *δV* is a constant representing the unit cell volume. Thus the timescale associated with cellular growth is *τ*_*g*_ = 1/*g*. We have considered the case of two structures growing from a shared pool. We implemented the growth of cell size and subunit abundance in a stochastic Gillespie simulation, and varied the timescale of structure growth to study the effect of cell growth on organelle size control. Specifically, we compare two distinct structure growth mechanisms—(i) the limiting pool *α* + *β* = 0, and the (ii) size-dependent growth model *α* + *β* = 1. The timescales of these two growth mechanisms are $\tau_{LP}∼\frac{1}{k^{+}}$ and $\tau_{\alpha \beta }∼\frac{1}{k^{+}+k^{-}}$, respectively.

**Fig 9:**
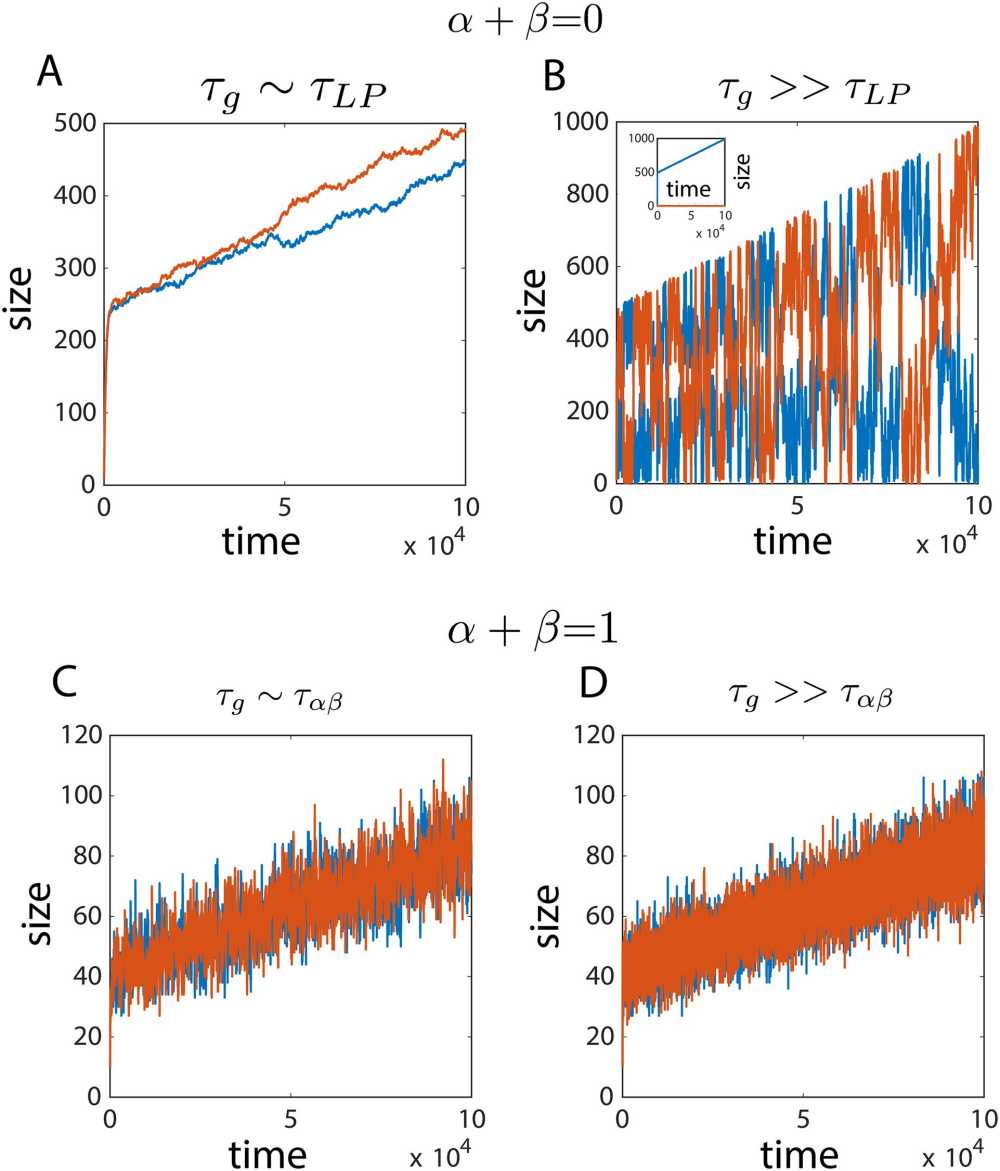
Effect of cellular growth on structure size control. (A,B) In the limiting pool model (*α* + *β* = 0), fast structure growth shows characteristic large fluctuations and loss of size control. In the slow growth regime, transient size control is observed without significant size fluctuations (B, Inset) With difference in growth rates of the structures (e.g., here *κ*_1_ = 2*κ*_2_), a winner-takes-all dynamics ensues and the structure with larger growth rate (blue) increases in size, while the other structure (red) remains small in size. (C,D) The size-dependent growth model (*α* + *β* = 1) provides local temporal control of structure size, with the structure size growing at the same rate as cellular growth.

In the limiting pool model, when structure growth is much faster than cellular growth (*τ*_*g*_ ≫ *τ*_*LP*_) large anti-correlated size fluctuations persist and there is no control of individual structure size (Fig 9B). But when the structure growth rate is comparable (or less than) to cellular growth (*τ*_*g*_ ∼ *τ*_*LP*_), transient control of structure size is observed, with suppressed size fluctuations (Fig 9A). In both cases, the individual mean size (over a time period ∼*τ*_*LP*_) increases as the total pool size increases in time. When there is competition between two non-identical structures, the faster growing structure takes up all the subunits and grows with cell size (Fig 9B,inset). The size-dependent growth model however, ensures local temporal control of structure size, with the mean size increasing with the growing cell size (Fig 9C and 9D). Size control is ensured at both the limits *τ*_*g*_ ≫ *τ*_*αβ*_ and *τ*_*g*_ ∼ *τ*_*αβ*_ in the size-dependent growth model.

## Discussion

In this study, we developed a theory for size-dependent growth of intracellular structures and organelles to uncover the design principles for robust size regulation of intracellular structures in the noisy environment of the cell, where stochastic fluctuations may be significant. Our study reveals that a size-dependent negative feedback control of the net growth rate of individual organelles underlies robust size control, when multiple of them compete for the same subunit pool. While the need for negative feedback in size homeostasis is well appreciated in literature, we demonstrate that our proposed feedback motif for size control is utilised by diverse subcellular structures, from one-dimensional filaments to three-dimensional organelles. In doing so, we connect our kinetic theory with known molecular processes in the cell. We show that our growth control model can also be utilized to assemble non-identical stable structures that may be important for cellular processes involving anisotropy and asymmetry. It is important to contrast our model with the limiting pool model for organelle growth control [1]. The latter provides a mechanism for organelle size scaling with cell size by sensing the subunit pool size, but fails to maintain the individual size of multiple competing organelles. The limiting pool model, however, succeeds in regulating the size of single structures because sensing the pool size is complementary to sensing the individual structure size in the case of a single structure.

Our growth model can also ensure size regulation of multiple structures in the case of subunit homeostasis when there is negative feedback between structure size and growth rate. Growth with subunit homeostasis does not lead to structure size scaling with cell size and the number of structures, emphasizing the need for a limiting subunit pool to preserve structure-to-cell size scaling. Subunit homeostasis can be important when structures are required to be maintained at a specific size regardless of cell size. In our proposed size-dependent growth model, it is possible to modulate the structure size to scale with cell size or be independent of cell size, by combining the features of subunit homeostasis and the limiting pool model. Individual structure size would scale with the cell size when the cell volume *V* is smaller than $\tilde{V}=M\kappa$, and saturates at $V\gg \tilde{V}$, where *M* is the number of structures assembled. This non-linearity in scaling behaviour due to size-dependent growth rates may be the underlying reason for the experimentally observed sub-linear scaling of organelle size with cell size at larger cell sizes [5, 10, 66].

Subunit abundance can increase during cell growth, as the abundance of many regulatory proteins increases with increasing cell size. It is therefore relevant to investigate the effect of cell growth (increasing cell size and subunit pool size) on the size control of intracellular structures. If cell growth rate is faster compared to the assembly rate of the structures, the limiting pool mechanism can maintain transiently stable sizes for multiple structures grown from a shared subunit pool. But when cell growth is much slower than structure growth, then the limiting pool model fails to regulate the size of individual structures, exhibiting large size fluctuations. In this case, a size-dependent negative feedback control of growth rate is required to achieve robust size control.

One of the key assumptions of our model is that the subunit pool is well mixed in the cytoplasm. In S3 Text, we relax this assumption and study the effect of subunit diffusion on the growth and size control of intracellular organelles. We find that the results of the spatially extended model with finite diffusion of subunits remain qualitatively similar to the predictions of our kinetic model for size-dependent growth in different parameter regimes. While the assembly of structures with slower diffusion of subunits takes longer to reach the steady state, the nature of the steady state or the size dynamics do not depend on the magnitude of the diffusion constant (S10 Fig). This can be simply understood from the fact that with slower diffusion and increasing distance between the structures, the growth kinetics of individual structures will quickly attain an equilibrium with the local subunit pool, but will take longer to interact with other structures to reach a global equilibrium.

In the presence of positive feedback between structure size and growth rate, we find bistable size distribution where structures dynamically fluctuate between a larger and a smaller assembly. Interestingly, the transition rate from the larger to the smaller structure becomes vanishingly small when the subunit pool is large (S12 Fig), giving rise to a single stochastically chosen large structure that is maintained for very long timescales. This elucidates a mechanism of spontaneous symmetry-breaking and polarity establishment, which is relevant for understanding the mechanism of bud formation in *S. cerevisiae* from the autocatalytic growth of Cdc42 clusters. We further show that with increasing size of the system (or cell) we can make a transition from dynamic, transient structures in small system size to a long-lived single structure in larger system size (S12 Fig). This result indicates the possibility of a size-dependent regulation of cell polarity enabling a cell state transition from apolar to polar, depending on cell size.

## Methods

### Stochastic simulations

We use the Gillespie algorithm [67] to simulate the stochastic growth of one or multiple structures from a common pool of subunits. At any time *t* the Gillespie algorithm uses two random variables drawn from an uniform distribution ($r_{1},r_{2}\in U\left(0,1\right)$), and the instantaneous propensities for all of the possible reactions to update the system in time according to the defined growth law. The propensities of the relevant reactions, i.e., the assembly and disassembly rates of the *i*^*th*^ structure are given by $K_{i}^{\text{on}}$ and $K_{i}^{\text{off}}$ respectively. For our growth model these propensities are functions of subunit pool size (*N*) and structure size (*n*_*i*_),

$$\begin{array}{ccc} K_{i}^{\text{on}} & = & k^{+}(\frac{N-\sum_{i=1}^{M}n_{i}}{V}){\left(1+n_{i}\right)}^{-\alpha }\;,\\ K_{i}^{\text{off}} & = & k^{-}n_{i}^{\beta }\;, \end{array}$$ (26)

where we are considering growth of *M* structures from a shared pool. The Gillespie algorithm computes the time for the next reaction at *t* + *τ* given the current state of the system (i.e., the propensities for all reactions) at time *t* where *τ* is given by-

$$\begin{array}{c} \tau =\frac{1}{\sum_{i=1}^{C}R_{i}}\text{log}(\frac{1}{r_{1}})\;, \end{array}$$ (27)

where $R_{i}$ is the propensity of *i*^*th*^ reaction and *C* is the total number of all possible reactions which is equal to 2*M* in our case. The second random variable *r*_2_ is used to select the particular reaction (*j*^*th*^ reaction) that will occur at *t* + *τ* time such that

$$\begin{array}{c} \frac{\sum_{i=1}^{j-1}R_{i}}{\sum_{i=1}^{C}R_{i}}\leq r_{2}<\frac{\sum_{i=1}^{j}R_{i}}{\sum_{i=1}^{C}R_{i}}\;. \end{array}$$ (28)

The condition for the first reaction (*j* = 1) is $0\leq r_{2}<\frac{R_{1}}{\sum_{i=1}^{C}R_{i}}$. The two steps defined by Eqs 27 and 28 are used recursively to compute the growth dynamics in time. We allow the structures to grow from the state *n*_*i*_ = 0, but disallow disassembly by setting *K*^off^ (*n*_*i*_ = 0) = 0, as the structure size cannot be negative.

## Supporting information

S1 Text The canonical limiting pool model. (PDF)

S2 Text Size-dependent growth model. (PDF)

S3 Text Size-dependent growth with finite diffusion of subunits. (PDF)

S4 Text Size-dependent growth of multiple structures. (PDF)

S5 Text Filament length control. (PDF)

S6 Text Feedback motif in nuclear growth model. (PDF)

S7 Text Bistable size distribution from autocatalytic growth. (PDF)

S8 Text Growth of multiple structures with constant subunit concentration. (PDF)

S1 Fig Failure of the limiting pool in controlling the size of multiple structures. (A) For a single structure, the limiting pool model can provide robust size control. The structure reaches a steady state size after an initial period of fast growth. (B) The steady state size distribution shows a unimodal peaked distribution, characterising a well-defined mean size for the structure. (C) The limiting pool mechanism captures structure size scaling with cell size. (D-E) Limiting pool fails to control the individual size for two structures grown from a shared pool of subunits, giving rise to large anti-correlated fluctuations (D). The total size of the structures is a well controlled quantity, with temporal stability (D) and unimodal peaked distribution (E). The individual size distributions are almost uniform in a range of 0 to *N* − *κ*^−1^*V* (E). (F) For many structures, the individual size distributions converge to an exponential distribution—i.e., the standard deviation of size fluctuations are as large as the mean size, which is indicative of poor size control.
(PDF)

S2 FigGrowth of structures with no size-dependent feedback: *α* + *β* = 0.(A) We study the growth dynamics of structures A, B and C with coefficients *α* and *β* that lie on the line *α* + *β* = 0. B corresponds to the canonical limiting pool model. (B) For the case A, there are two fixed points and linear stability analysis indicates one to be a saddle node and the other to be an unstable fixed point near origin. (C) The fixed points in case A move away from each other as *κ* increases. (D) For case C, we find a single fixed point and linear stability indicates that the fixed point is stable. (E) The steady-state size given by the stable fixed point increases with *κ*, saturating at high *κ* values. The blue (${\dot{n}}_{1}=0$) and yellow (${\dot{n}}_{2}=0$) lines (in panels B and D) are the nullclines and the red arrows represent the flow in the *n*_1_ − *n*_2_ phase plane. (f) Structure size distributions in the three cases in (A), given by the solution chemical master equation solution (solid line) and stochastic simulations (points). (G-I) Structure size dynamics obtained from stochastic growth simulations show large anti-correlated fluctuations (in all three cases), leading to a failure in size control. Parameters: *N* = 50 and *κ* = 0.1.(PDF)

S3 FigGrowth of structures in robust size control regime: *α* + *β* > 0.(A) We study the dynamics of structure growth at four points A, B, C and D on the *α* + *β* = 1 line which lies in the regime where there is negative feedback control of structure growth. (B) For the case A we find four fixed points—one unstable node, one stable node and two saddle nodes inferred from linear stability. (C-E) In all three cases B, C and D we find a single stable node from linear stability. The blue (${\dot{n}}_{1}=0$) and the yellow (${\dot{n}}_{2}=0$) lines (in panels B-E) are the nullclines and the red arrows represent the flow in the *n*_1_−*n*_2_ phase space. (F) The chemical master equation solution (black line) and stochastic simulations (points) predicts the same size distributions in all four cases, with a well defined mean value and comparatively small standard deviation, reflecting size control. (G-J) The temporal dynamics from stochastic simulations show well defined mean size at all times. Despite the difference in individual *α*, *β* values the statistical properties of the size dynamics are the same in all the four cases.(PDF)

S4 FigGrowth of a single structure in the autocatalytic growth regime: *α* + *β* < 0.(A) We study the size dynamics of a growing structure at six different parameter regimes, namely the points A, B, C, D, E and F lying on the line *α* + *β* = −1. In this regime, there is a size-dependent positive feedback on the growth of the structure. (B) Stability diagram showing growth rate $\dot{n}=F\left(n\right)$ vs structure size *n* in the deterministic model. Top row: For *β* < 0 there are only two fixed points, one stable and the other unstable, showing no apparent presence of bistability. The second stable fixed point (open circle) is obtained from treating the divergence at boundary (*n* = 0) and considering *K*^off^(0) = 0. Bottom row: when *β* > 0, there are three fixed points—two stable and one unstable, and thus the system is bistable. (C) Structure size distribution obtained from solution to the chemical master equation solution (solid line), and from stochastic simulations (points), showing bimodality of size distributions in all six parameter regimes. Inset: Temporal evolution of structure size in cases A and F, illustrating that residence times in the two stable states is dependent on the *α* and *β* values. (D) Evolution of the fixed points (solid lines) as a function of growth rate *κ* showing that for a single structure the bistability is only present in the range κ* < *κ* < *κ*^*c*^. The black and red lines indicate the position of the stable and unstable fixed points, respectively. Heatmap shows *dn*/*dt*. (E) Steady-state probability distribution *P*(*n*), as a function of *κ* and *n*. For all calculations *N* = 50 and *κ* = 0.0022 (except in e panel) was taken.(PDF)

S5 Fig Growth of two structures in bistable regime: *α* + *β* < 0. (A) We study the size dynamics of two growing structures with coefficients *α* and *β* satisfying the condition *α* + *β* = −1 (points P, Q, R and S). (B-E) The phase portrait in *n*_1_ and *n*_2_ plane show that there are two fixed points when *β* ≤ 0 (panels B-D), one saddle node and another unstable node. We obtain the additional boundary fixed points (open circles) by separately treating the divergence at the boundary. We get one stable and one unstable fixed points at the proximity of each boundary *n*_1_ = 0 and *n*_2_ = 0. In contrast when *β* > 0 (panel E), we find two stable nodes, leading to bistable size dynamics. (E, inset) There are four more fixed points at small size—one stable, one unstable and two saddle nodes. The stable point in small size has a very small basin of stability and it disappears at higher *κ* values. (F) The chemical master equation solution (solid line) and results from stochastic simulations (points) show that size distributions in all four cases are bimodal arising from bistability in size dynamics. Inset: Temporal evolution of the size of the structures in cases P and S, illustrating that residence times in the two stable states is dependent on the individual *α* and *β* values. For all calculations, *N* = 50 and *κ*_1_ = *κ*_2_ = *κ* = 0.005 (except in the last panel) were taken.
(PDF)

S6 Fig Effect of growth rate and subunit abundance on structure size regulation. Here we show the failure of size regulation for small growth rate or subunit density. (A) Mean size and standard deviation in size increases monotonically and eventually saturates with increasing growth rate *κ*. (B) CV in size decreases as *κ* increases. The high CV value at small *κ* shows that the small structures at very low growth rates lacks robust control of size. (C) Mean size and standard deviation in size increases monotonically with increasing the total pool size *N*. (D) CV in size decreases as *N* increases. (E) Size fluctuations are larger than the mean size for very small values of growth rate *κ*. (F) CV is also larger than unity for small growth rates. We define the critical growth rate *κ*_0_ to be the growth rate where *CV* = 1. (G-H) The structures do not grow to be much larger in the low growth rate regime, and the size distribution can be fitted well to an exponential function, *P*(*n*) = λ*e*^−λ*n*^, where λ is a constant. (I) CV decreases with increasing growth rate *κ* and total pool size *N*, underlying a transition of size dynamics from *no-growth* to robust size regulation for *κ* > *κ*_0_. The *κ*_0_ value decreases as the total pool size increases indicating that this transition in size dynamics can occur due to reduction in subunit density. For all the results discussed up to this point we take *α* + *β* = 1. (J-K) Large size fluctuations compared to the mean size and a characteristic exponential size distribution for *κ* < *κ*_0_ is also present in the limit *α* + *β* = 0. (L-M) Large size fluctuations compared to the mean size and a characteristic exponential size distribution for *κ* < *κ*_0_ is also seen in the case of autocatalytic growth (*α* + *β* < 0). The effects of feedback in the growth becomes apparent when *κ* > *κ*_0_. These results were obtained from the solution to the master equation for two growing structures with *N* = 50.
(PDF)

S7 Fig State transition diagram for stochastic size dynamicsof *M* competing structures. Illustration of all possible transitions into and from the state {*n*_1_, *n*_2_, …, *n*_*M*_} via assembly and disassembly processes.
(PDF)

S8 Fig Size distribution for the growth of multiple identical structures competing for a limiting subunit pool. (A-C) Individual size distribution of *M* identical structures in the limit *α* + *β* = 0. (D-F) With negative feedback control of growth (*α* + *β* > 0), robust size regulation is achieved for any *M* number of structures, with the mean size decreasing with increasing *M* with fixed pool size *N*. (G-I) The size dynamics is bistable in the presence of positive feedback, *α* + *β* < 0. The parameter values are *N* = 50, *κ* = 1 for all cases except (F) and (I), where *N* = 20.
(PDF)

S9 FigLength-dependent disassembly rate ensures length control of multiple actin filaments.Length dependent disassembly rate arises when monomers switch between different states with distinct disassembly rates. (A) Schematic of filament growth with monomer state switching. (B) At steady-state the probability of ATP-bound monomer *P*_*b*_(*x*) decreases towards the pointed end with a length scale λ^−1^. Parameters: *k*^+^ = 2, $k_{b}^{-}=0.5$, $k_{u}^{-}=2.5$, *w*_1_ = 0.01 and *w*_2_ = 0.02. (C) The effective disassembly rate of the filament, computed from a stochastic simulation (using Gillespie algorithm) of filament growth with nucleotide hydrolysis and in a limiting subunit pool. $k_{d}^{\text{eff}}\left(L\right)$ increases linearly with filament length. Parameters: $k_{b}^{-}=10$ s^−1^, $k_{u}^{-}=200$ s^−1^, *ρ*_0_ ≃ 3 *μ*M, *w*_1_ = 0.005 s^−1^ and *w*_2_ = 0.01 s^−1^. The parameter *k*^+^ was varied to span the range of filament lengths.(PDF)

S10 Fig Effect of diffusion on size-dependent growth. (A,B) Size regulation fails in the parameter regime *α* + *β* = 0 in the presence of diffusion. In the case of slower diffusion, the size distribution takes longer time (*T*, in simulation units) to converge to the almost uniform size distribution predicted by solution to the chemical master equation. The colours indicate the simulation time *T*, and the curves of same color represent the size distribution of the two structures that converge to the same distribution at longer times. (C,D) In presence of negative feedback, *α* + *β* > 0, the size distribution quickly relaxes to the steady state distribution and this relaxation timescale does not depend on the diffusion constant. (E) In presence of positive feedback, *α* + *β* < 0, we see bistability and the resulting bimodal size distribution does not depend on the value of the diffusion constant. (F,G) The diffusion affects the statistical properties of the size dynamics. A slower diffusion (F) will lead to a longer residence time in one of the steady states. The parameter values are *N* = 50 and *κ* = 1 everywhere except in (E-G) where *κ* = 0.005.
(PDF)

S11 Fig Autocatalytic growth of multiple structures. (A-C) We study growth of multiple structures from a common pool of subunits, with size-dependent positive feedback *α* + *β* = −0.2. This makes the growth process autocatalytic. We see a transition in the size dynamics as we increase the overall density of subunits (by changing total amount *N* while keeping volume *V* fixed). (A) The structures hardly grow at low subunit density. (B) At intermediate subunit density, bistability in size distribution emerges. The bigger structure captures most of the subunits, but suddenly starts declining in size due to stochastic fluctuations, when the other structures grow to be bigger. This creates a “flickering” growth pattern of multiple structures. (C) At a higher subunit density, we observe an initial growth of multiple structures. At a later time only a single structure forms while the others “die-out” in the competition. This mechanism can be used to make sure only a single structure gets built inside the cell which is important in various cases of polarity establishment and spontaneous symmetry breaking.
(PDF)

S12 Fig System size dependent polarity transition in autocatalytic growth of multiple structures. (A-D) We study growth of multiple structures (*M* = 4) from a common pool of subunits in a system size *V*, with size-dependent positive feedback *α* = −1, *β* = 0. This makes the growth process autocatalytic. We see a transition in the size dynamics as we increase the system size *V* while keeping the subunit density the same. This shows a size dependent polarity establishment process where below a critical size of the system all structures grow and shrink dynamically. But above a critical size, the residence time becomes exceedingly large making transitions virtually impossible in an experimentally relevant timescale. Thus only a stochastically selected structure remain in large size, establishing a polarity in the cell. The pool size *N* and cell volume *V* was changed 20%, 30% and 50% in panels B,C and D, respectively. Parameters (for panel A): *N* = 20, *V* = 1, *κ* = 0.02.
(PDF)
