# Supplementary material for: Size regulation of multiple organelles competing for a limiting subunit pool
Source: PLoS Comput Biol. 2022 Jun 17;18(6):e1010253. doi: 10.1371/journal.pcbi.1010253 (PMC9246132; doi:10.1371/journal.pcbi.1010253)
Supplement: S1 Text — (PDF) [file pcbi.1010253.s001.pdf]

S1 Text. The canonical limiting pool model

During the growth of one or multiple structures via the limiting pool mechanism, we assume that a cell maintains a constant total amount of subunits N (no net production or degradation of subunits). As the structures grow in size, the subunit pool depletes (so density of subunits decreases) and consequently the assembly rate decreases. So the growth dynamics for the ith structure when M number of structures are grown from a shared subunit pool, can be written as

$$\dot{n_i} = k_i^+ \left(\frac{N - \sum_{i=1}^{M} n_i}{V} \right) - k_i^- \tag{1}$$

where $k_i^\pm$ is the assembly and disassembly rates for i^{th} structure and n_i is the size of the ith structure in number of monomers/subunits. The available amount of subunit at any given time is given by $N_{av} = N - \sum_{i=1}^{M} n_i$. Now for a single structure ($M = 1$) growth the equation becomes

$$\dot{n} = k^+ \left(\frac{N - n}{V} \right) - k^- \tag{2}$$

with the parameters having usual meaning as before. The steady-state size of the structure is given by ($\dot{n} = 0$)

$$n^* = \left(\rho_0 - \frac{1}{\kappa} \right) V \tag{3}$$

where overall subunit density $\rho_0 = \frac{N}{V}$ and $\kappa = \frac{k^+}{k^-}$. This result from the deterministic description is identical to the mean size calculated from the respective stochastic description. If the overall concentration of subunits is maintained at a constant value (i.e., ρ_0 is independent of N and V) we obtain a linear scaling of structure size with cell size (S1C Fig).

Now if we consider multiple structures growing from a shared pool with equal growth rates ($k_i^\pm = k^\pm$ for all i), then we get a system of under-determined equations at steady-state (i.e., $\dot{n_i} = 0$ for all i) leads us to

$$k^+ \left(\frac{N - \sum_i n_i}{V} \right) - k^- = 0 \tag{4}$$

where the equations for n_i's are identical and any combination of sizes that satisfies $\sum_i n_i = \left(\rho_0 - \kappa^{-1} \right) V$ is a solution for the above equation. This hints at the failure of size control for the growth of multiple structures from a limiting subunit pool.

Notably the total size of all structures is robustly controlled (S1D-E Fig). A stochastic description for the growth of multiple structures captures the failure of size control as studied recently [1]. In the case of two structures, the size distributions are uniform. The size distribution for many structures becomes exponential where the standard deviation of size fluctuations is of the same order as the mean size (S1F Fig).

Reference

[1] Mohapatra L, Lagny TJ, Harbage D, Jelenkovic PR, Kondev J. The limiting-pool mechanism fails to control the size of multiple organelles. Cell systems. 2017;4(5):559–567.
