# Supplementary material for: Size regulation of multiple organelles competing for a limiting subunit pool
Source: PLoS Comput Biol. 2022 Jun 17;18(6):e1010253. doi: 10.1371/journal.pcbi.1010253 (PMC9246132; doi:10.1371/journal.pcbi.1010253)
Supplement: S2 Text — (PDF) [file pcbi.1010253.s002.pdf]

S2 Text. Size-dependent growth model

Here we present the mathematical details and analysis of the general model for size-dependent growth of intracellular structures, in different parameter regimes. The intracellular structures are assembled from a limiting subunit pool of size N. Due to the noisy environment of the cell, subunits undergo stochastic binding and unbinding kinetics with rates given by K^{on} and K^{off} respectively. The assembly and disassembly rates further depend on the size of the individual structures. Let us first consider a single structure of n subunits growing from a subunit pool of total size N within a cell of unit volume. The assembly and disassembly rates are given by

$$\begin{aligned} K^{\text{on}}(n) &= k^{+}(N-n)(1+n)^{-\alpha} \\ K^{\text{off}}(n) &= k^{-}n^{\beta} \end{aligned} \tag{1}$$

where k^{+} and k^{-} are the bare assembly and disassembly rates, and the coefficients α and β define the size dependence of growth rates. The chemical master equation for the probability $P(n,t)$ of realizing a structure of size n at time t is given by,

$$\begin{aligned} \frac{\mathrm{d}P(n,t)}{\mathrm{dt}} &= K^{\text{on}}(n-1)P(n-1,t) + K^{\text{off}}(n+1)P(n+1,t) \\ &\quad - \left(K^{\text{on}}(n) + K^{\text{off}}(n)\right)P(n,t)\,. \end{aligned} \tag{2}$$

The above equation can be solved at steady state using the detailed balance condition: $k^{-}n^{\beta}P(n) = k^{+}(N-n+1)(n)^{-\alpha}P(n-1)$. The normalized size distribution is given by

$$P(n) = \frac{1}{C_N}\left(\frac{\kappa^{n}N!}{(N-n)!(n!)^{\alpha+\beta}}\right) \tag{3}$$

where $\kappa = \frac{k^{+}}{k^{-}}$ and the normalization constant is $C_N = \sum_{n=0}^{N}\frac{\kappa^{n}N!}{(N-n)!(n!)^{\alpha+\beta}}$. For the limiting pool model described in earlier works [1, 2], $K^{\text{on}} = k^{+}(N-n)$, $K^{\text{off}} = k^{-}$, such that the size distribution is given by

$$P(n) = \frac{\kappa^{-(N-n)}e^{-\frac{1}{\kappa}}N!}{(N-n)!\Gamma(N+1,\frac{1}{\kappa})} \tag{4}$$

where Γ is the incomplete gamma function. For a single structure growing from a limited amount of subunit pool, no additional size dependence in growth rates is required for robust size regulation. The dependence of the assembly rate on the available pool size contains the necessary negative feedback control of structure size. The same principle does not apply when there are more than one structures growing from a shared subunit pool.

Consider two structures of sizes n_1 and n_2, growing from a shared pool of N subunits, with assembly and disassembly rates given by,

$$K_i^{\mathrm{on}}(\{n_i\}) = k_i^+(N - \sum_{i=1}^{2} n_i)(1+n_i)^{-\alpha}$$
$$K_i^{\mathrm{off}}(\{n_i\}) = k_i^- n_i^{\beta} \ , \tag{5}$$

where $i = 1, 2$ and k_i^+ and k_i^- are the bare assembly and disassembly rates of ith structure. Now the chemical master equation of the joint probability distribution $P(n_1, n_2, t)$ is given by

$$\begin{aligned}\frac{\mathrm{d}P(n_1,n_2,t)}{\mathrm{dt}} &= K_1^{\mathrm{on}}(n_1-1,n_2)P(n_1-1,n_2,t) + K_2^{\mathrm{on}}(n_1,n_2-1)P(n_1,n_2-1,t)\\ &+K_1^{\mathrm{off}}(n_1+1,n_2)P(n_1+1,n_2,t) + K_2^{\mathrm{off}}(n_1,n_2+1)P(n_1,n_2+1,t)\\ &-\Big(K_1^{\mathrm{on}}(n_1,n_2)+K_2^{\mathrm{on}}(n_1,n_2)+K_1^{\mathrm{off}}(n_1,n_2)+K_2^{\mathrm{off}}(n_1,n_2)\Big)P(n_1,n_2,t).\end{aligned}$$

The steady-state joint probability distribution can be obtained by using the following detailed balance conditions

$$k_1^- n_1^{\beta} P(n_1,n_2) = k_1^+(N-(n_1+n_2-1))n_1^{-\alpha}P(n_1-1,n_2)$$
$$k_2^- n_2^{\beta} P(n_1,n_2) = k_2^+(N-(n_1+n_2-1))n_2^{-\alpha}P(n_1,n_2-1) \ , \tag{6}$$

which yields,

$$\begin{aligned}P(n_1,n_2) &= \left(\frac{\kappa_1(N-(n_1+n_2-1))}{n_1^{\alpha+\beta}}\right)P(n_1-1,n_2)\\ &= \left(\frac{\kappa_1(N-(n_1+n_2-1))}{n_1^{\alpha+\beta}}\right)\left(\frac{\kappa_2(N-(n_1+n_2-2))}{n_2^{\alpha+\beta}}\right)P(n_1-1,n_2-1)\end{aligned}$$

where $\kappa_1 = \frac{k_1^+}{k_1^-}$ and $\kappa_2 = \frac{k_2^+}{k_2^-}$. The above relation can be used recurrently to determine the steady-state joint probability distribution as

$$P(n_1,n_2) = \left(\frac{\kappa_1^{n_1}\kappa_2^{n_2}}{(n_1!)^{\alpha+\beta}(n_2!)^{\alpha+\beta}}\right)\left(\frac{N!}{(N-(n_1+n_2))!}\right)P(0,0) \tag{7}$$

where $P(0,0)$ is the probability of finding both the structures at zero size and it can be calculated using the normalization condition $\sum_{n_1,n_2=0,n_1+n_2\leq N}^{N} P(n_1,n_2) = 1$. We can then compute the marginals as,

$$P(n_1) = \sum_{n_2=0,n_1+n_2\leq N}^{N}\left(\frac{\kappa_1^{n_1}\kappa_2^{n_2}}{(n_1!)^{\alpha+\beta}(n_2!)^{\alpha+\beta}}\right)\left(\frac{N!}{(N-(n_1+n_2))!}\right)P(0,0). \tag{8}$$

We note that the size distribution and its statistical properties depend on the parameter $\alpha+\beta$ rather than the individual values of α and β as defined in Eq. (8). This magnitude of $\alpha+\beta$

determines the overall strength of size-dependent feedback, and the sign of $\alpha+\beta$ determines the signature of feedback in the growth process. For instance, there is an overall size-dependent negative feedback for $\alpha+\beta>0$ and a positive feedback for $\alpha+\beta<0$. There is no net size-dependent feedback on growth for $\alpha+\beta=0$. In the following, we present the analysis of our model in these three regimes of size-dependent growth of multiple structures.

Dynamics of the mean size. Using the master equation in Eq. (2), we can derive the dynamical equation for the mean size, given by the first moment $\langle n\rangle = \sum_{n=0}^{N} nP(n,t)$. However, given the nonlinearity of the system, it is not straightforward to derive a closed form for the dynamics of mean size for all values of α and β. We first consider the simple case of $\alpha=0$ and $\beta=0$, which describes the canonical limiting pool model. Multiplying Eq. (2) by n and then summing over n we get,

$$\sum_{n=0}^{N}\frac{\mathrm{d}}{\mathrm{dt}}(nP(n,t)) = \sum_{n=0}^{N} k^{+}n(N-n+1)P(n-1,t) + \sum_{n=0}^{N} k^{-}nP(n+1,t) - \sum_{n=0}^{N}\left(k^{+}n(N-n)+k^{-}n\right)P(n,t)\,. \tag{9}$$

By changing variables $n-1\to n$ and $n+1\to n$ in the first and the second terms respectively, we arrive at the following equation for the mean size $\langle n\rangle$

$$\frac{\mathrm{d}}{\mathrm{dt}}(\langle n\rangle) = k^{+}(N-\langle n\rangle) - k^{-}\;, \tag{10}$$

where we used the condition $k^{-}=0$ at $n=0$, in order to set the term $k^{-}P(0,t)\to 0$ in the second term on RHS Eq. (9). Similarly for $\alpha=0$, $\beta=1$ the dynamics of the mean size can be obtained as

$$\frac{\mathrm{d}}{\mathrm{dt}}(\langle n\rangle) = k^{+}(N-\langle n\rangle) - k^{-}\langle n\rangle\,. \tag{11}$$

The above two cases are examples of $\alpha+\beta=0$ and $\alpha+\beta>0$, where for those particular choices of α and β the mean size dynamics can be described by the simple form $\dot{n} = K^{\mathrm{on}}(n) - K^{\mathrm{off}}(n)$. Next we consider two examples in the autocatalytic growth regime $\alpha+\beta<0$. In the particular case $\alpha=-1$, $\beta=0$ we get

$$\sum_{n=0}^{N}\frac{\mathrm{d}}{\mathrm{dt}}(nP(n,t)) = \sum_{n=0}^{N} k^{+}n^{2}(N-n+1)P(n-1,t) + \sum_{n=0}^{N} k^{-}nP(n+1,t) - \sum_{n=0}^{N}\left(k^{+}n(n+1)(N-n)+k^{-}n\right)P(n,t)\,. \tag{12}$$

Making the substitutions $n-1 \to n$ and $n+1 \to n$ in the first and the second term of the RHS of the above equation, and setting $k^- P(0,t) \to 0$ we arrive at

$$\frac{\mathrm{d}}{\mathrm{dt}}(\langle n \rangle) = k^+(N - \langle n \rangle + N\langle n \rangle - \langle n^2 \rangle) - k^- < n > , \tag{13}$$

where the second order moment cannot be reduced in terms of the first moment without additional information about the size distribution. Thus the RHS of the dynamics for mean size is not simply given by the RHS of the rate equation: $k^+(N - \langle n \rangle)(\langle n \rangle + 1) - k^- \langle n \rangle$. Similarly for $\alpha = 0$, $\beta = -1$ the dynamics of the mean size is given by

$$\frac{\mathrm{d}}{\mathrm{dt}}(\langle n \rangle) = k^+(N - \langle n \rangle) - k^- \langle \frac{1}{n} \rangle , \tag{14}$$

containing an inverse moment which is not necessarily same as $\frac{1}{\langle n \rangle}$. Thus it is not always possible to derive simple forms for the dynamics of the mean size from the master equation due to the presence of nonlinearities in the assembly and the disassembly rates. However, to gain a conceptual understanding of the dynamics, we consider the following approximate deterministic description of the mean size given by

$$\frac{dn_i}{dt} = k_i^+(N - \sum_{i=1}^{M} n_i)(1 + n_i)^{-\alpha} - k_i^- n^\beta \tag{15}$$

where n_i is the size of ith structure and $i = 1..M$. As discussed here and later in this supplementary material, the deterministic version of our model does not always accurately describe the size dynamics obtained from a stochastic model.

CASE 1: $\alpha + \beta = 0$, NO SIZE-DEPENDENT FEEDBACK IN GROWTH

The case $\alpha = 0$, $\beta = 0$ corresponds to the canonical limiting pool model [1] with constant disassembly rate and a subunit pool size-dependent assembly rate

$$\begin{aligned} K_i^{\mathrm{on}}(\{n_i\}) &= k_i^+(N - \sum_{i=1}^{2} n_i) \\ K_i^{\mathrm{off}}(\{n_i\}) &= k_i^- \ . \end{aligned} \tag{16}$$

The steady-state joint distribution for two structures is given by,

$$P(n_1, n_2) = \kappa_1^{n_1} \kappa_2^{n_2} \left(\frac{N!}{(N - (n_1 + n_2))!} \right) P(0,0) \, . \tag{17}$$

The limiting pool mechanism alone fails to regulate the size of multiple structures growing from a shared subunit pool [2]. The case $\alpha + \beta = 0$ presents a class of models which behave similar to the

canonical limiting pool model despite the growth rates being dependent on the individual structure size. Failure of size-regulation for $\alpha+\beta=0$ arises from the fact the feedback from size-dependent assembly is nullified by the opposite sign feedback from size-dependent disassembly. Such cases arise naturally in considering the growth of higher-dimensional structures. For example, two spheres growing from a shared pool of subunits via assembly/disassembly of subunits from the surface will have $K^{\mathrm{on}}(n) \sim (1+n)^{\frac{2}{3}}$ and $K^{\mathrm{off}}(n) \sim n^{\frac{2}{3}}$. In this case size-regulation cannot be achieved since $\alpha=-\frac{2}{3}$, $\beta=\frac{2}{3}$, resulting in $\alpha+\beta=0$.

Here we present three different instances of the model $\alpha+\beta=0$, where there is no size-dependent feedback on growth. In doing so, we analyze the deterministic rate equations and perform stochastic growth simulations for two identical structures ($\kappa_1=\kappa_2=\kappa=k^+/k^-$) to highlight the importance of stochastic growth description over the deterministic model in understanding the true dynamic behavior of the system. We choose three points marked as A, B and C on the line $\alpha+\beta=0$ in the $\alpha-\beta$ parameter space (S2A Fig) where B defines the canonical limiting pool model with $\alpha=0$, $\beta=0$, and the other two cases have non-zero α and β coefficients. The deterministic rate equations for the case B cannot be solved analytically as the problem reduces to an under-determined system of equations. The nullclines in the phasespace of n_1 and n_2 overlap completely as they are defined by the same equation: $k^+(N-n_1-n_2)-k^-=0$. The other two cases (A and C) are solvable and we find fixed points and their stability by performing a standard linear stability analysis around the fixed points. For case A ($\alpha=1$, $\beta=-1$) we find two fixed points – one unstable and another saddle point (S2B-C Fig). For case C ($\alpha=-1$, $\beta=1$) we find one stable fixed point (S2D-E Fig). Although the deterministic analysis indicates a stable fixed point for $\alpha=-1$, $\beta=1$ (S2D Fig), stochastic simulations show large anti-correlated size fluctuations leading to a failure in size control (S2F-H Fig). Thus the existence of size regulation cannot be discerned solely from a stability analysis of the deterministic rate equations. From the solution of the chemical master equations and stochastic growth simulations we find that all the three cases exhibit very similar temporal dynamics with large size fluctuations and identical size distributions (S2F-I Fig).

CASE 2: $\alpha+\beta>0$, NEGATIVE FEEDBACK CONTROL OF STRUCTURE SIZE

Here we show that robust size control can be achieved in the regime $\alpha+\beta>0$, where there is an overall size-dependent negative feedback on growth rate. We discuss the size dynamics using both deterministic and stochastic descriptions for four different α, β values (A, B, C and D) on

the $\alpha+\beta=1$ line (S3A Fig), where the deterministic rate equations are analytically solvable. The phase portrait with nullclines and fixed points of the deterministic rate equations show that in all the cases (A, B, C and D) there is only one stable fixed point (n_1^*, n_2^*), representing the steady-state mean size of the structures (S3B-E Fig), in agreement with stochastic simulations.

The deterministic description can only provide information about the mean size but the existence of a stable fixed point in deterministic equations does not always guarantee robust size control as we have seen in the case $\alpha+\beta=0$. So a stochastic analysis is important to understand size regulation of intracellular structures. The solution of the chemical master equation and numerical simulations show that the size dynamics in this case has a well defined mean size with fluctuations smaller than the mean. The resulting size distribution does not depend on the individual values of α or β but only depends on $\alpha+\beta$ (S3F-J Fig).

CASE 3: $\alpha+\beta<0$, POSITIVE FEEDBACK AND AUTOCATALYTIC GROWTH

During autocatalytic growth, a lager structure grows faster than a smaller one, due to a positive feedback in growth. The size dynamics, in the presence of a limiting subunit pool, shows bistability and a bimodal distribution of size when there is an overall size dependent positive feedback in growth, $\alpha+\beta<0$. This bistability or bimodality of size distribution is seen for any number of structures growing from a shared pool but may not be present for arbitrary choice of parameters. We discuss these features in details below.

Bistability in the growth of a single structure. A single growing structure shows bistability when $\alpha+\beta<0$. We consider six points (A to F) on the line $\alpha+\beta=-1$ in the $\alpha-\beta$ plane (S4A Fig), and analyze the features of bistability using both the deterministic and the stochastic descriptions of the model. We find that a linear stability analysis of the deterministic rate equations shows existence of bistability (existence of two stable fixed points) for $\alpha+\beta<0$ and $\beta>0$ (S4B Fig). The singularity at the boundary ($n=0$) has to be taken into account to examine bistability in the regime $\alpha+\beta<0$ and $\beta<0$, as the deterministic equations exhibit only one stable fixed point for $\beta<0$ (S4B Fig, top row). We consider $K^{\text{off}}(0)=0$ at the boundary (as the structure size is physically constrained $n\geq 0$) and obtain the second fixed point at $n=0$. Around this fixed point, the dynamics strongly push the solution towards $n=0$ making it a stable fixed point. For any point on the $\alpha+\beta=-1$ line the size distribution is bimodal and does not depend on the individual values of α and β (S4C Fig). Other statistical properties such as the residence time

(average time the system spends in one state, where the states are defined by the stationary values of n) can differ depending on the α and β values (S4C Fig, inset). It is important to note that the bistability in size occurs only within a range of the reaction rate ratio $\kappa^* < \kappa < \kappa^c$. For $\kappa > \kappa^c$ the size dynamics is monostable and a single structure always grows to be very large taking up almost the entire subunit pool (S4D-E Fig).

Bistability in the growth of two structures. For two structures growing from a shared pool of subunits, we find bistability in size dynamics as well as a bimodal size distribution for $\kappa > \kappa^*$ and $\alpha + \beta < 0$. To illustrate this, we analyze both the deterministic and the stochastic descriptions of the model in four different parameter regimes: points P, Q, R and S on the line $\alpha + \beta = -1$ line in the $\alpha - \beta$ plane (S5A Fig). The fixed points of the deterministic rate equations can be analytically obtained and a linear stability analysis about the fixed points shows that for all values of $\beta \leq 0$ (P, Q and R) two stable fixed points (stable nodes) are not present (S5B-D Fig). Bistability in this parameter regime can be understood by separately treating the singularity at the boundary, $n_{1,2} = 0$, for $\beta < 0$. Near the boundaries, the phase trajectory flows constrain the solution in the neighbourhood of the boundary (S5B-C Fig). The solution is also physically constrained in the region n_1, $n_2 \geq 0$, and the boundary state cannot disassemble, i.e., $K^{\text{off}}_{1,2}(0) = 0$ [?] leading to two new *boundary states* given by $\dot{n}_1 = 0, n_2 = 0$ and $n_1 = 0, \dot{n}_2 = 0$. Solution of the boundary states yields four more fixed points: two stable and two unstable, explaining bistability in the region $\alpha + \beta < 0$ and $\beta < 0$ (S5B-C Fig).

However, for $\beta > 0$ the bistability in size dynamics can be simply understood from linear stability analysis of the deterministic model, as linear stability shows existence of two stable nodes (S5E Fig), consistent with the bimodality in structure size distribution obtained from stochastic model (S5F Fig). The case for $\alpha = -1, \beta = 0$ is different as it does not have any singularity. Though we have obtained the additional fixed point from the solution of $\dot{n}_1 = 0, n_2 = 0$ and $n_1 = 0, \dot{n}_2 = 0$ (using the constraint $K^{\text{off}}_{1,2}(0) = 0$), it is important to note that the deterministic equation used is different from the equation for mean size obtained from the master equation (Eq. 13). This discrepancy may hint towards the existence of purely stochastic bistability as seen in the presence of autocatalytic reactions in many cases [3–5]. Indeed we see such effects with a slightly modified description of the size-dependent assembly rate (not shown) for the case of size-dependent positive feedback. Solution to the chemical master equations and numerical simulations of the stochastic model show that the size distribution is the same for all cases (S5F Fig), and does not depend on the individual α and β values but on $\alpha + \beta$. Other statistical quantities such as the residence

time may depend on the individual coefficients (S5F Fig, inset). The average structure size in the large-size state depends on κ and consumes almost the whole pool of subunits as κ increases. It should be noted that in autocatalytic growth of multiple structures, the master equation solution shows bistability (i.e., bimodal size distribution) for all $\kappa > \kappa^*$, as there is no monostability at higher growth rates as seen for the case of a single structure.

Reference

[1] Goehring NW, Hyman AA. Organelle growth control through limiting pools of cytoplasmic components. Curr Biol. 2012;22(9):R330–R339.

[2] Mohapatra L, Lagny TJ, Harbage D, Jelenkovic PR, Kondev J. The limiting-pool mechanism fails to control the size of multiple organelles. Cell systems. 2017;4(5):559–567.

[3] Togashi Y, Kaneko K. Transitions induced by the discreteness of molecules in a small autocatalytic system. Phys rev lett. 2001;86(11):2459.

[4] Artyomov M N, Das J, Kardar M and Chakraborty, A K. Purely stochastic binary decisions in cell signaling models without underlying deterministic bistabilities. PNAS. 2007;104(48):18958–18963.

[5] Padmanabhan P, Goodhill G J. Axon growth regulation by a bistable molecular switch. Proc. R. Soc. B: Biol. Sci. 2018;285(1877):20172618.
