# Supplementary material for: Size regulation of multiple organelles competing for a limiting subunit pool
Source: PLoS Comput Biol. 2022 Jun 17;18(6):e1010253. doi: 10.1371/journal.pcbi.1010253 (PMC9246132; doi:10.1371/journal.pcbi.1010253)
Supplement: S3 Text — (PDF) [file pcbi.1010253.s003.pdf]

S3 Text. Size-dependent growth with finite diffusion of subunits

To study the effect of subunit diffusion on size-dependent growth and size control of intracellular structures, we consider two identical structures, n_A and n_B, with bare growth rate ratio $\kappa_A = \kappa_B = \kappa$. The total system is composed of two well-mixed subsystems (A and B) that can exchange subunits via diffusion. Each subsystem has one structure growing from an available pool of subunits m_A or m_B respectively. The subunits from the subsystem subunit pools can diffuse at a rate $(D/\delta^2)m_{A,B}$ where δ is the separation length between the two growing structures and D is the diffusion constant of the subunits. In the following, we show that slow subunit diffusion increases the timescale of reaching a steady-state size but there is no qualitative change in the nature of size regulation or size dynamics. The structures being identical in bare growth rates, the resulting size distributions are identical as well. So we discuss the results for a single structure in each of the subsystems.

In the case $\alpha + \beta = 0$, a slower diffusion constant delays the timescale for size relaxation but the steady-state shows the characteristic uniform size distribution with a failure of size regulation (S10A-B Fig). Regardless of the value of the diffusion constant the steady state size is same as predicted by the solution to the chemical master equation (CME) (S10A-B Fig). In the regime of robust size regulation ($\alpha + \beta > 0$) each structure quickly attains a steady-state size. The timescale for size relaxation does not depend on the diffusion of subunits as we start with the initial condition $m_A = m_B$. The resulting size distribution matches with the CME solution for a single structure (S10C-D Fig) growing in a pool of half the size ($N/2$) of the total pool size of the system $N = n_A + n_B + m_A + m_B$. In the regime $\alpha + \beta < 0$, the nature of bistability in size distribution does not change in presence of subunit diffusion. The resulting size distributions do not depend on diffusion (S10E Fig) but other statistical properties such as residence time may depend (smaller residence time for faster subunit diffusion) on diffusion of subunits (S10F-G Fig).

The effect of diffusion on a larger scale can be understood using a coarse-grained description in the form of reaction-diffusion equations for the density fields of subunit pool and the structures. In this case, continuum variables such as the structure density $S(x,t)$ and the subunit pool density $P(x,t)$ can be calculated by coarse graining over the structure mass and subunit mass over a small volume [1]. The resultant equations can be written as

$$\partial_t S = D_S \partial_x^2 S + k^+ P S^{-\alpha} - k^- S^{\beta} \ , \quad (1)$$

$$\partial_t P = D_P \partial_x^2 P - k^+ P S^{-\alpha} + k^- S^{\beta} \ . \quad (2)$$

where D_S and D_P are the diffusion constants of the structure and the pool, with $D_S \ll D_P$. This continuum treatment shows that we find homogeneous growth of structures when $(\alpha + \beta \geq 0)$ and spatially localized structure growth (symmetry breaking and pattern formation) when $(\alpha + \beta < 0)$ [1]. A more detailed analysis of spatial regulation of growth control is beyond the scope of this present study.

Reference

[1] Cornwall Scoones J, Banerjee DS, Banerjee S. Size-regulated symmetry breaking in reaction-diffusion models of developmental transitions. Cells. 2020;9(7):1646.
