# Supplementary material for: Size regulation of multiple organelles competing for a limiting subunit pool
Source: PLoS Comput Biol. 2022 Jun 17;18(6):e1010253. doi: 10.1371/journal.pcbi.1010253 (PMC9246132; doi:10.1371/journal.pcbi.1010253)
Supplement: S4 Text — (PDF) [file pcbi.1010253.s004.pdf]

S4 Text. Size dependent growth of multiple structures

The results shown for two structures in our study can be generalized to multiple structures. Let us consider $M > 2$ structures growing from a shared pool of N subunits in total. The growth rates for i^{th} structure are given by-

$$\begin{aligned} K_i^{\text{on}}(\{n_i\}) &= k_i^+(N - \sum_{i=1}^{M} n_i)(1+n_i)^{-\alpha} \\ K_i^{\text{off}}(\{n_i\}) &= k_i^- n_i^\beta \ , \end{aligned} \tag{1}$$

where $i = 1, \ldots, M$. The probability of finding the system in a particular state $\{n_i\}$ at time t is given by $P(n_1, n_2, \ldots, n_M, t)$. The master equation can be written considering the rate of change of probability $P(n_1, n_2, \ldots, n_M, t)$ to be equal to the influx of probabilities in this state $P(n_1, n_2, \ldots, n_M, t)$ from $2M$ different adjacent states, subtracted by the outflux of probabilities to those $2M$ states (S7 Fig).

The steady state joint probability can be obtained using the M detailed balance relations given by

$$k_i^- n_i^\beta P(\ldots, n_i, \ldots) = k_i^+ n_i^{-\alpha} \left(N - (\sum_{i=1}^{M} n_i - 1) \right) P(\ldots, n_i - 1, \ldots) \, . \tag{2}$$

Using the above equation (Eq. 2) recursively, the steady state joint distribution can be calculated to be

$$P(n_1, n_2, \ldots, n_M) = \left(\kappa_1^{n_1} \ldots \kappa_M^{n_M} \right) \left(\frac{N!}{(N - \sum_{i=1}^{M} n_i)!} \right) \left(\frac{1}{(n_1!)^{\alpha+\beta} \ldots (n_M!)^{\alpha+\beta}} \right) P(0, 0, \ldots, 0) \tag{3}$$

where $P(0, 0, \ldots, 0)$ is the normalization constant and $\kappa_i = \frac{k_i^+}{k_i^-}$. The marginal distribution for any i^{th} structure $P(n_i)$ can be calculated by summing over the other structures, for example

$$P(n_1) = \sum_{n_2=0, S_n \leq N}^{N} \sum_{n_3=0, S_n \leq N}^{N} \cdots \sum_{n_M=0, S_n \leq N}^{N} \left(\frac{\left(\kappa_1^{n_1} \ldots \kappa_M^{n_M} \right) N!}{(N - \sum_{i=1}^{M} n_i)!} \right) \left(\frac{1}{(n_1!)^{\alpha+\beta} \ldots (n_M!)^{\alpha+\beta}} \right) \tag{4}$$

where $S_n = \sum_{i=1}^{M} n_i$. This sum becomes computationally very expensive as the number of summations to be computed goes as N^M. It is important to note that the growth model of more than 2 structures does not change the mathematical form of the size distribution, but only adds additional terms in the product. Thus we do not expect the results to change qualitatively for $M > 2$. We computed the size distribution for up to four ($M = 4$) identical structures ($\kappa_i = \kappa$ for any i^{th} structure) and checked the characteristics of the size distributions in the three feedback regimes

using the marginal size distribution (Eq. 4). For $\alpha + \beta = 0$, there is no size-dependent feedback of growth, and the size distribution exhibits fluctuations (standard deviation) of the same order as the mean size (S8A-C Fig). With increasing number of structures the size distribution becomes exponential in nature. In the regime of robust size regulation, $\alpha + \beta > 0$, the size distribution has well defined mean for any number of structures (S8D-F Fig). In the regime of autocatalytic growth, $\alpha + \beta < 0$, we observe bistability in size for any number of structures (S8G-I Fig). The mean size decreases when we increase number of structures growing from a shared pool keeping the pool size (N) fixed.
