# Supplementary material for: Size regulation of multiple organelles competing for a limiting subunit pool
Source: PLoS Comput Biol. 2022 Jun 17;18(6):e1010253. doi: 10.1371/journal.pcbi.1010253 (PMC9246132; doi:10.1371/journal.pcbi.1010253)
Supplement: S5 Text — (PDF) [file pcbi.1010253.s005.pdf]

S5 Text. Filament length control

Antenna model for microtubule length control. Microtubule filaments grow via the addition of tubulin dimers as the subunits. In presence of Kip-3 motors, the disassembly rate of microtubule filaments becomes length dependent, as found experimentally [1, 2]. The motors bind to the filaments and move towards the minus end and detaches a tubulin dimer from the filament. The amount of motors walking on a filament increases with the increasing filament length thus the disassembly rate increases with filament length. This length dependence of disassembly rate was found to be linear [1]. Hence the disassembly rate for the ith microtubule of size n_i can be written as $k^- n_i$ and the deterministic description of the growth for microtubule antenna model becomes

$$\dot{n_i} = k^+ \left(\frac{N - \sum_{i=1}^{M} n_i}{V} \right) - k^- n_i \tag{1}$$

where M is the number of microtubule filaments growing from a shared pool of tubulin. This description maps exactly to our general model with $\alpha = 0$, $\beta = 1$. Here k^+ and k^- are the bare assembly and disassembly rates, respectively, and k^- can be an increasing function of motor density [1], which can tune the mean microtubule filament size

$$n^* = \frac{N}{\left(M + \frac{k^-}{k^+} V \right)}. \tag{2}$$

Nucleotide hydrolysis model for F-actin length control. It has been reported that treadmilling-like growth of actin filaments can lead to an effective length-dependent disassembly rate, via nucleotide hydrolysis of the the bound monomers [3]. Here we take a simple case of treadmilling-like growth of one actin filament where the bound G-actin monomers can be in either of two states – ATP-bound state or ADP-bound state. We consider a minimal model where the filament length increases via binding of ATP-bound monomers at the barbed end with binding rate k^+, and length decreases only via unbinding of monomers from the pointed end (S9A Fig). The unbinding rate depends on the monomer state, and we denote k_b^- and k_u^- as the unbinding rates for ATP-bound and ADP-bound monomers respectively. If $P_b(x,t)$ and $P_u(x,t)$ define the probabilities of finding the monomer at position x (in the frame of the filament with origin at barbed end) and time t in the ATP-bound and the ADP-bound states, respectively, then we can express $P_b(x, t + \Delta t)$ as

$$P_b(x, t + \Delta t) = k^+ \Delta t P_b(x - \Delta x, t) - k^+ \Delta t P_b(x,t) + w_1 \Delta t P_u(x,t) - w_2 \Delta t P_b(x,t) \ , \tag{3}$$

which leads to the following master equation –

$$\partial_t P_b(x,t) = -k^+ \, \partial_x P_b(x,t) + w_1 - (w_1 + w_2 + 1) P_b(x,t) \ , \tag{4}$$

where w_1 and w_2 are the transition rates of monomers going from ADP-bound state to the ATP-bound state and *vice versa*. This simplified analytical description does not take into account the contributions from the end subunit unbinding on the evolution of the subunit next to the pointed end. Rather it treats all the subunits to be a subunit in the bulk of the filament. Assuming the system reaches a steady-state ($\partial_t P_b = 0$) at filament length $\bar{L}$, we can solve for $P_b(x)$ as (S9B Fig)

$$P_b(x) = A + (1 - A)e^{-\lambda x} \tag{5}$$

where $A = \frac{w_1}{w_1+w_2+1}$ and $\lambda = \frac{w_1+w_2+1}{k^+}$. Here we have employed the assumption that only ATP-bound monomers are binding to the barbed end which lead us to the following boundary condition: $P_b(0) = 1, P_u(0) = 0$. At steady-state, the effective disassembly rate (k_d^{eff}) at the pointed end and the assembly rate at the barbed end should be the same, such that

$$k^+ = k_d^{\text{eff}} = k_b^- P_b(\bar{L}) + k_u^- (1 - P_b(\bar{L})) \ . \tag{6}$$

Using Eq.5 we can then compute the length dependence of the disassembly rate

$$k_d^{\text{eff}}(\bar{L}) = k_u^- + (k_b^- - k_u^-) \left(A + (1 - A)e^{-\lambda \bar{L}} \ . \right) \tag{7}$$

The steady-state filament length $\bar{L}$ is given by

$$\bar{L} = \lambda^{-1} \ln \left(\frac{1 - A}{P - A} \right) \ , \tag{8}$$

where $P = \frac{k^+ - k_u^-}{k_b^- - k_u^-}$. In the limit of small λ, i.e. $w_1 + w_2 + 1 \ll k^+$, we get

$$k_d^{\text{eff}} = C_0 + C_1 \bar{L} \tag{9}$$

where $C_0 = k_b^-$ and $C_1 = \lambda(k_u^- - k_b^-)(1 - A)$. This limit approximately corresponds to our size-dependent growth model with $(\alpha, \beta) = (0, 1)$, and is verified by stochastic simulation of the full model (S9C Fig).

Reference

[1] Varga V, Helenius J, Tanaka K, Hyman AA, Tanaka TU, Howard J. Yeast kinesin-8 depolymerizes microtubules in a length-dependent manner. Nat Cell Biol. 2006;8:957–962.

[2] Varga V, Leduc C, Bormuth V, Diez S, Howard J. Kinesin-8 motors act cooperatively to mediate length-dependent microtubule depolymerization. Cell. 2009;138:1174–1183.

[3] Erlenkämper C, Kruse K. Uncorrelated changes of subunit stability can generate length-dependent disassembly of treadmilling filaments. Physical Biology. 2009;6(4):046016.
