# Supplementary material for: Size regulation of multiple organelles competing for a limiting subunit pool
Source: PLoS Comput Biol. 2022 Jun 17;18(6):e1010253. doi: 10.1371/journal.pcbi.1010253 (PMC9246132; doi:10.1371/journal.pcbi.1010253)
Supplement: S6 Text — (PDF) [file pcbi.1010253.s006.pdf]

S6 Text. Feedback motif in nuclear growth model

Nuclear growth model. During the growth and expansion of nucleus, the surrounding microtubule structures assist in the nuclear growth. The growth equations written in terms of nuclear radius and microtubule length are given by

$$\dot{n} = K^{+}(\bar{L}, R_n)\left(\frac{N-n}{V}\right) - k^{-}n \,, \tag{1}$$

$$\dot{L}_i = k_m^{+}\left(\frac{N_m - \sum_i L_i}{V}\right) - k_m^{-}L_i \,, \tag{2}$$

where R_n is the nucleus radius, n and L_i are the sizes of NE (in building block units) and the ith microtubule filament, respectively. The total amount of tubulin and NE building blocks are given by N_m and N, respectively, and V is the cell (or system) volume. Here $k^{\pm}$ and $k_m^{\pm}$ are the bare rates of assembly and disassembly for NE and microtubules, respectively. It is important to note that the above equations (in terms of R_n and n) will not change when nuclear growth by nucleoplasm assembly is considered but the relationship between R_n and n will change. We shall now simplify the microtubule growth description by only considering the dynamics of mean microtubule length given by

$$\dot{\bar{L}} = k_m^{+}\left(\frac{N_m - M\bar{L}}{V}\right) - k_m^{-}\bar{L} \,, \tag{3}$$

where $\bar{L}$ is the mean (over all filaments) length of microtubule and M is the number of filaments in the system. The assembly rate in Eq. 1 is given by

$$K^{+}(\bar{L}, R_n) = k^{+}(4\pi/3)\left((\bar{L}+R_n)^3 - R_n^3\right) \,. \tag{4}$$

Now, when the filaments are much smaller compared to the nuclear radius, $\bar{L} \ll R_n$ the Eq. 1 can be written as

$$\begin{aligned}\dot{n} &= k^{+}(4\pi/3)N_{av}R_n^3\left((1+\frac{\bar{L}}{R_n})^3 - 1\right) - k^{-}n \\ &\simeq k^{+}(4\pi/3)N_{av}R_n^3\left(\frac{3\bar{L}}{R_n}\right) - k^{-}n \\ &= k^{+}(4\pi\bar{L})N_{av}R_n^2 - k^{-}n\end{aligned} \tag{5}$$

where $N_{av} = \frac{N-n}{V}$. This approximate growth description gives rise to two equations when considered in the context of nuclear growth by surface assembly (NE) and nucleoplasm assembly (NV):

$$\dot{n} = k^{+}(4\pi\bar{L})N_{av}n - k^{-}n \,, \qquad R_n^2 \sim n \text{ for NE} \tag{6}$$

$$\dot{n} = k^{+}(4\pi\bar{L})N_{av}n^{2/3} - k^{-}n \,, \qquad R_n^3 \sim n \text{ for NV} \,. \tag{7}$$

These two equations show the size dependent positive feedback in assembly rate when the aster size is smaller compared to the nucleus size. In the later stage of growth we reach the other limit where $\bar{L} >> R_n$. The Eq. 1 in this limit can br written like:

$$\begin{aligned} \dot{n} &= k^+(4\pi/3)N_{av}\bar{L}^3\left((1+\frac{R_n}{\bar{L}})^3 - \frac{R_n^3}{\bar{L}^3}\right) - k^- n \\ &\simeq k^+(4\pi\bar{L})N_{av}\bar{L}^3 - k^- n\,, \qquad \text{as}\,\frac{R_n}{\bar{L}} \to 0\,. \end{aligned} \quad (8)$$

This corresponds to the general model with $\alpha = 0\,, \beta = 1$. Note that in this limit both considerations (NE and NV) give rise to this equation as the disassembly rate is proportional to either nuclear envelope area (in NE) or nucleus volume (in NV) both of which scale with $\sim n$ in the respective models.
