# Supplementary material for: Size regulation of multiple organelles competing for a limiting subunit pool
Source: PLoS Comput Biol. 2022 Jun 17;18(6):e1010253. doi: 10.1371/journal.pcbi.1010253 (PMC9246132; doi:10.1371/journal.pcbi.1010253)
Supplement: S7 Text — (PDF) [file pcbi.1010253.s007.pdf]

S7 Text. Bistable size distribution from autocatalytic growth

Kip-3 and tubulin limiting pool model. In a recent study by Rank *et al* [1], it was demonstrated how Kip-3 motors that disassemble microtubules subunits (the tubulin dimers) from the plus-end give rise to bistable microtubule length distribution. Here, we show how this mechanism is related to our model in the limit $\alpha + \beta < 0$, where we find bistable size distribution due to autocatalytic growth from size-dependent positive feedback and negative-feedback from the limiting pool. In Rank *et al*'s study, the assembly rate is given by

$$K_{\mathrm{on}} = \gamma_0 \left(c_T - \frac{L}{\delta_L V} \right) \tag{1}$$

where L is the filament, V is the cell/system volume, $c_T = N/V$ is the initial subunit density, and δ_L is the subunit length. The volume V was considered to be an accessible area around the microtubule from where the motors can bind [1] but here we consider well mixed solution and take V to be the volume of the cell/system. Varying the value of V did not change the qualitative results in the original study [1]. The constant γ_0 is the same as the bare assembly rate, k^+, in our description. Disassembly occurs at the microtubule plus-end, when a Kip-3 motor reaches the end and falls off with a subunit. Thus the disassembly rate is given by

$$K_{\mathrm{off}} = \rho_+(t)\,\Delta \tag{2}$$

where $\rho_+(t)$ is the probability that the plus-end has a motor at time t, and Δ is the dissociation rate in the presence of a motor. The estimate of the probability leads to the following expression [1] for the disassembly rate

$$\rho_+(t)\,\Delta = \frac{w_A L}{\delta_L} - \left(\frac{(w_A + w_D) w_A L^2}{2\delta_L^2 v} \right) \,, \tag{3}$$

where w_A and w_D are the rates at which Kip-3 motors bind and unbind from the filament, respectively, and v is the rate at which motors move towards the plus end of the microtubule. The binding rate w_A depends on the cytosolic motor density c_m as $w_A = w_A^0\,(c_m - m/V)$, where w_A^0 is a constant, m is the abundance of bound motors, and V is the system volume. Given that the bound motor abundance equilibrates very fast compared to the microtubule growth dynamics [1], we can assume that w_A is constant. The length dynamics of microtubules are described by the following equation

$$\dot{L} = \delta_L\,(K_{\mathrm{on}} - K_{\mathrm{off}})\,. \tag{4}$$

The length here is $L = n\delta_L$ where n is the number of tubulins in the filament. The subunit density is $c_T = \frac{N}{V}$, where N is the total subunit pool size and V is the system size. Now the length dynamics can be re-written in terms of the subunit number as follows,

$$\dot{n} = k^+ \left(\frac{N_{av}}{V} \right) + C_1 n^2 - C_2 n \; , \tag{5}$$

where $C_1 = \frac{w_A(w_A+w_D)}{2v}$ and $C_2 = w_A$. The above equation approximately maps to our size-dependent growth model with $\alpha = -2$ and $\beta = 1$, belonging to the regime $\alpha + \beta < 0$. We have shown that in this regime our model exhibits bistable size distribution, which agrees with the reported results of Rank et al. Interestingly this model has a regime where the positive feedback is very weak (i.e., $\frac{k^+ N_{av}}{V} \gg C_1 n^2$). In this regime, microtubule growth can be mapped to our model with $\alpha = 0, \beta = 1$, leading to robust length control for multiple microtubule filaments competing for a limiting pool. This is consistent with the absence of bistability in low motor concentrations [1]. However our reduced description here cannot capture the observed monostability at high motor concentration and a more detailed analysis is required to address this issue.

Reference

[1] Rank M, Mitra A, Reese L, Diez S, Frey E. Limited Resources Induce Bistability in Microtubule Length Regulation. Phy rev lett. 2018;120(148101):1301–1305.
