# Supplementary material for: Size regulation of multiple organelles competing for a limiting subunit pool
Source: PLoS Comput Biol. 2022 Jun 17;18(6):e1010253. doi: 10.1371/journal.pcbi.1010253 (PMC9246132; doi:10.1371/journal.pcbi.1010253)
Supplement: S8 Text — (PDF) [file pcbi.1010253.s008.pdf]

S8 Text. Growth of multiple structures with constant subunit concentration

Multiple structures growing from a shared subunit pool with a constant subunit concentration (i.e., structure growth does not exhaust the subunit pool which is always maintained at some constant concentration) can be described by the following equation

$$\dot{n}_i = k_i^+ \rho - k_i^- \ , \tag{1}$$

where $k_i^{\pm}$ is the assembly and disassembly rates for i^{th} structure and n_i is the size of the ith structure in number of subunits. The structures will keep growing without bound when $\dot{n}_i > 0$.

This growth cannot regulate size unless it is at the critical subunit density ρ_c given by $\dot{n}_i = 0$ -i.e., $\rho_c = k_i^-/k_i^+$. Even then there is no equilibration of size is possible in this case. Recovery from this failure in size control can be achieved with size-dependent negative feedback

$$\dot{n}_i = k_i^+ \rho (1 + n_i)^{-\alpha} - k_i^- n_i^\beta \tag{2}$$

where $\alpha + \beta > 0$. We take a simple case of two structures where the corresponding master equation for the growth of two structures of size n_1 and n_2 is given by

$$\begin{aligned}\frac{\mathrm{d}P(n_1, n_2, t)}{\mathrm{dt}} &= k_1^+ \rho n_1^{-\alpha} P(n_1 - 1, n_2, t) + k_2^+ \rho n_2^{-\alpha} P(n_1, n_2 - 1, t) \\ &+ k_1^- (n_1 + 1)^\beta P(n_1 + 1, n_2, t) + k_2^- (n_2 + 1)^\beta P(n_1, n_2 + 1, t) \\ &- \left(k_1^+ \rho (n_1 + 1)^{-\alpha} + k_2^+ \rho (n_2 + 1)^{-\alpha} + k_1^- n_1^\beta + k_2^- n_2^\beta \right) P(n_1, n_2, t) \ ,\end{aligned}$$

where $P(n_1, n_2, t)$ is the probability that the two structures have sizes n_1 and n_2 at time t. The steady-state probability is obtained by solving the above master equation using the following detailed balance conditions –

$$\begin{aligned} k_1^- n_1^\beta P(n_1, n_2) &= k_1^+ \rho n_1^{-\alpha} P(n_1 - 1, n_2) \\ k_2^- n_2^\beta P(n_1, n_2) &= k_2^+ \rho n_2^{-\alpha} P(n_1, n_2 - 1) \ , \end{aligned} \tag{3}$$

which yields –

$$P(n_1, n_2) = \left(\frac{\kappa_1 \rho}{n_1^{\alpha+\beta}} \right) \left(\frac{\kappa_2 \rho}{n_2^{\alpha+\beta}} \right) P(n_1 - 1, n_2 - 1) \tag{4}$$

where $\kappa_1 = \frac{k_1^+}{k_1^-}$ and $\kappa_2 = \frac{k_2^+}{k_2^-}$. We can use this relation to iteratively compute the steady-state probability distribution:

$$P(n_1, n_2) = \left(\frac{\kappa_1^{n_1} \kappa_2^{n_2} \rho^{n_1+n_2}}{(n_1!)^{\alpha+\beta} (n_2!)^{\alpha+\beta}} \right) P(0, 0) \ , \tag{5}$$

where $P(0,0)$ is the probability of finding both the structures at zero size and it can be calculated using the normalization condition. This exact solution of steady state joint distribution of size may or maynot be written in a closed functional form, but we can easily calculate the marginals $P(n_1)$ and $P(n_2)$ by summing over the other variable –

$$P(n_{1,2}) = \sum_{n_{1,2}=0}^{\infty} \left(\frac{\kappa_1^{n_1} \kappa_2^{n_2} \rho^{n_1+n_2}}{(n_1!)^{\alpha+\beta}(n_2!)^{\alpha+\beta}} \right) P(0,0). \quad (6)$$

We compute this sum numerically to calculate the discrete size distributions.
