# Supplementary figures and images for: Size regulation of multiple organelles competing for a limiting subunit pool
Source: PLoS Comput Biol. 2022 Jun 17;18(6):e1010253. doi: 10.1371/journal.pcbi.1010253 (PMC9246132; doi:10.1371/journal.pcbi.1010253)

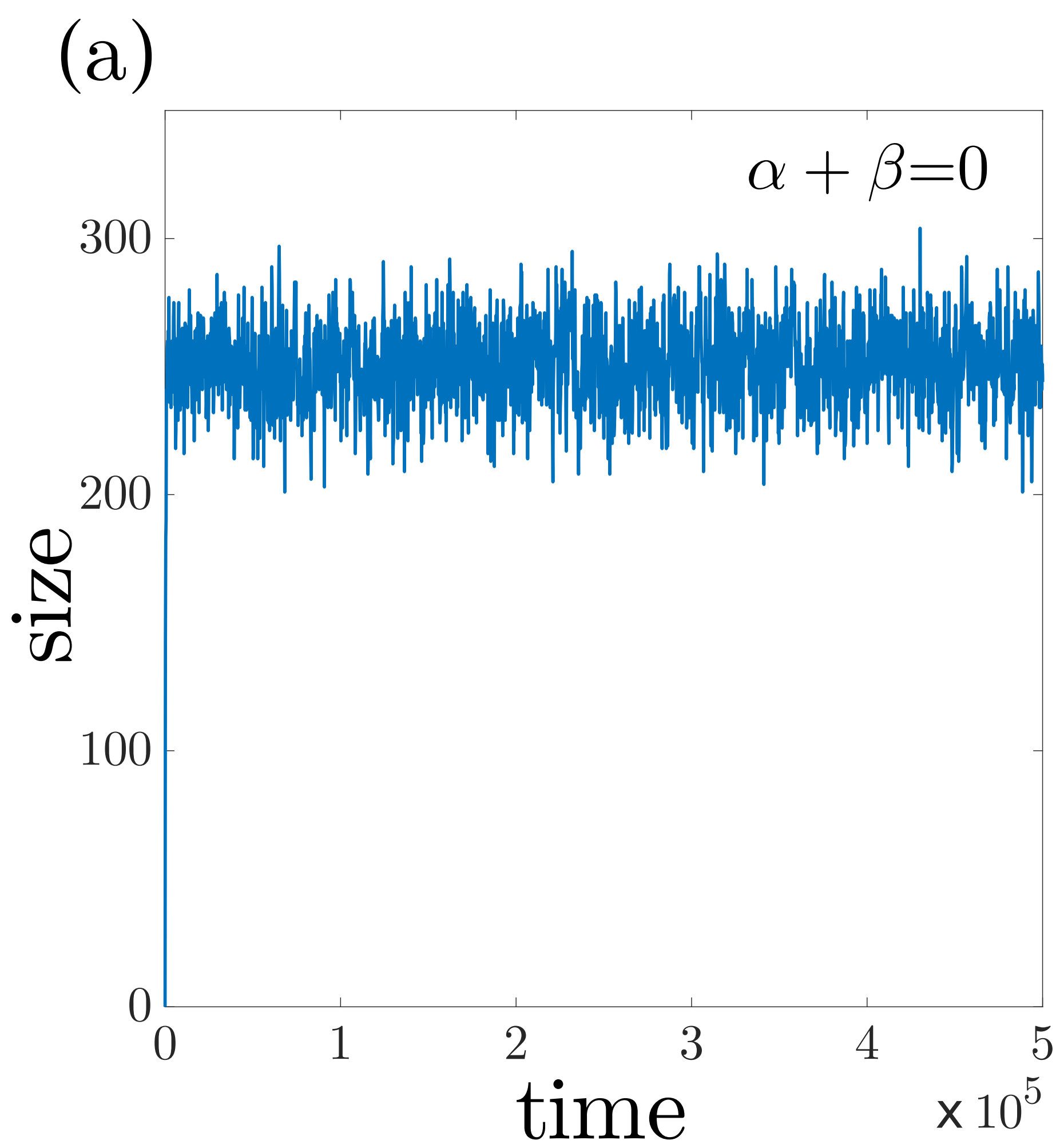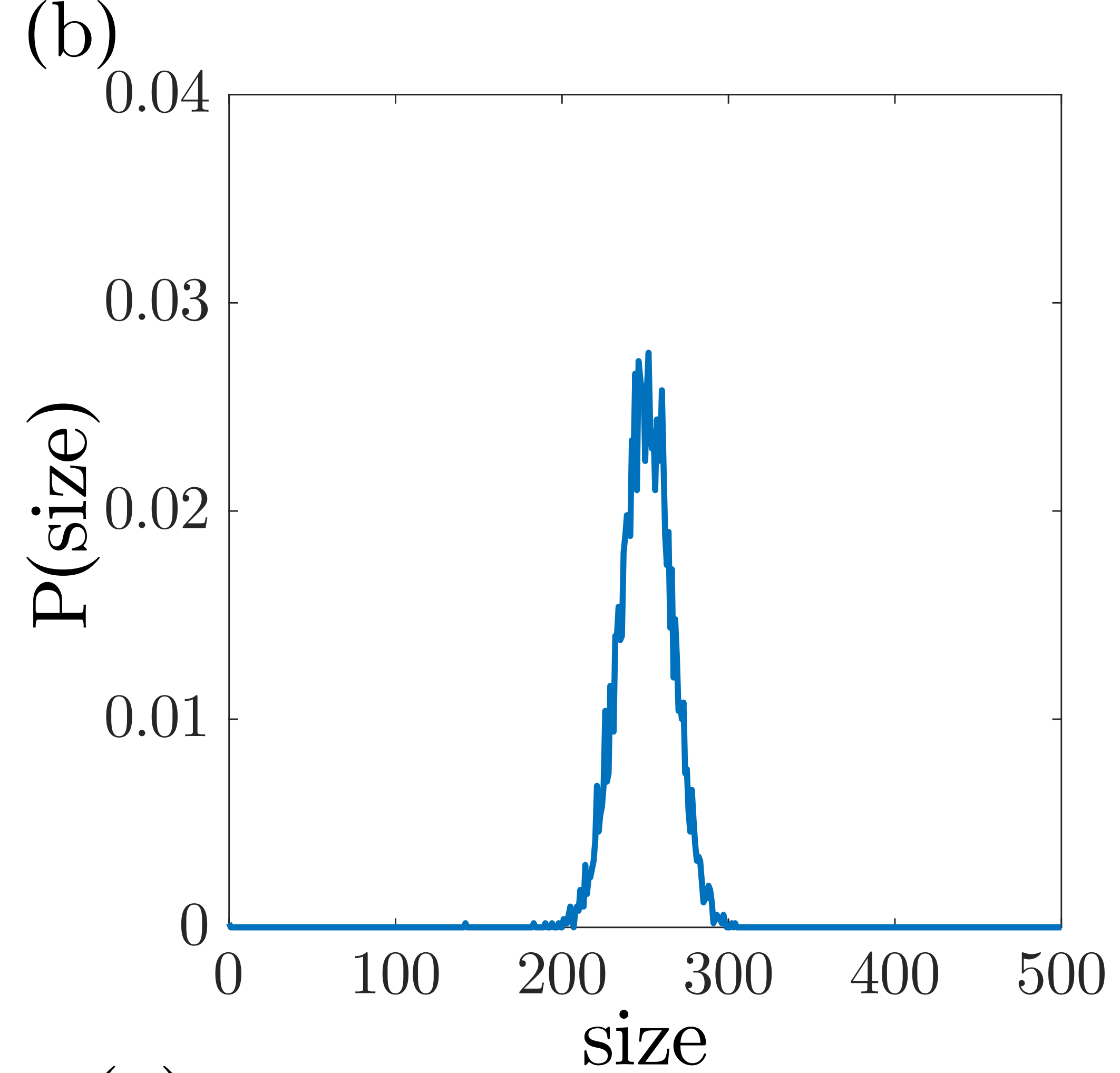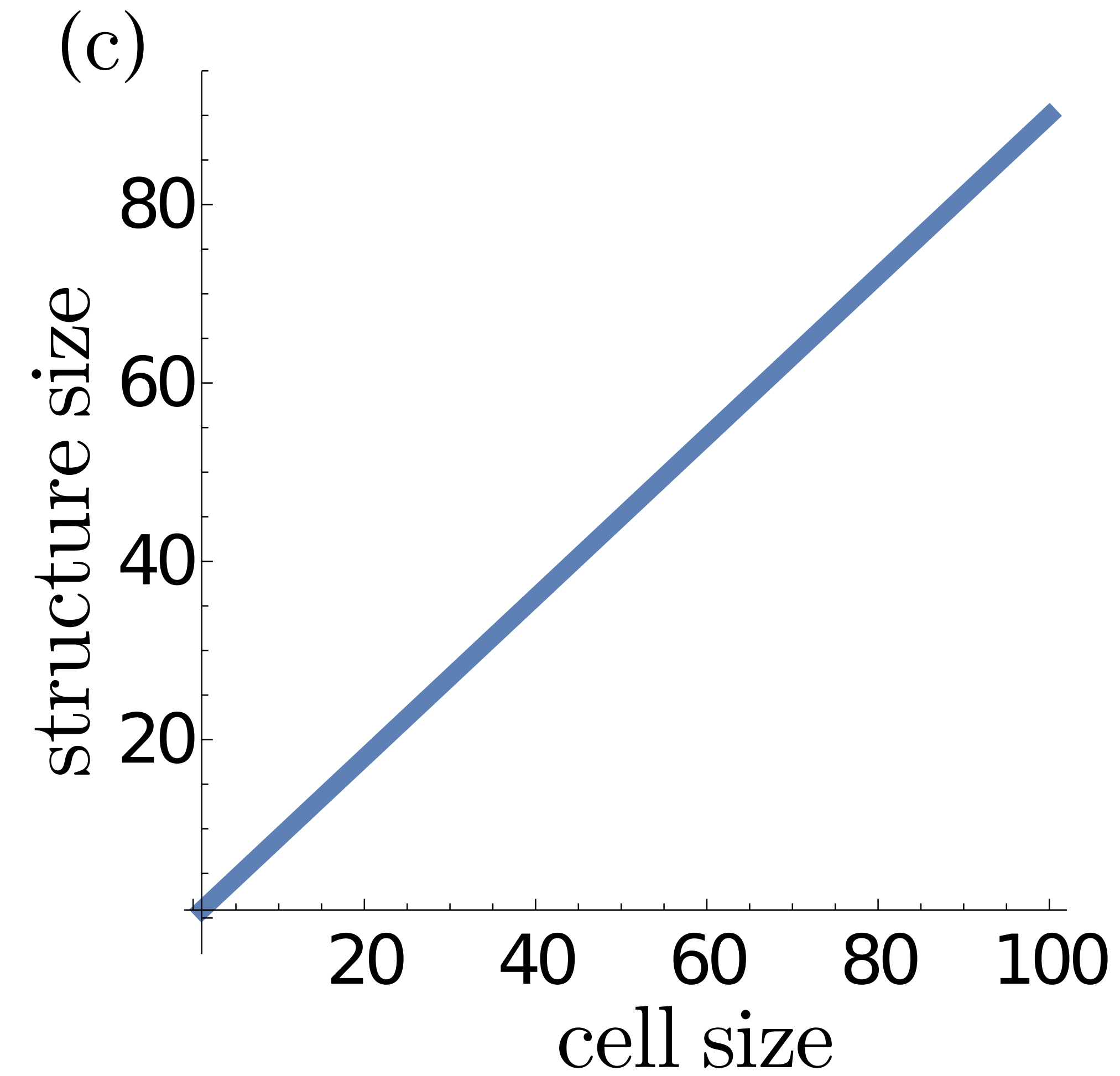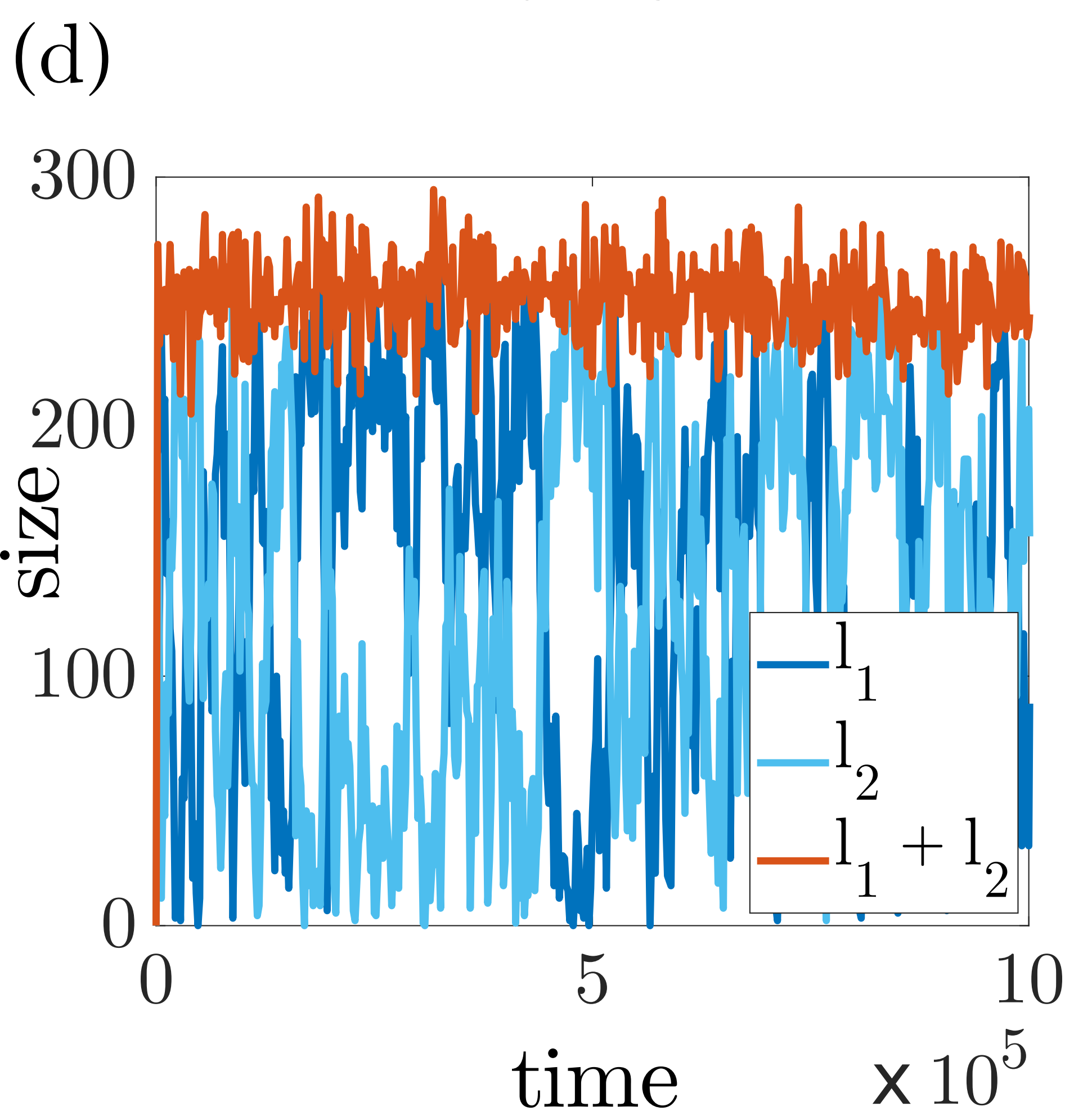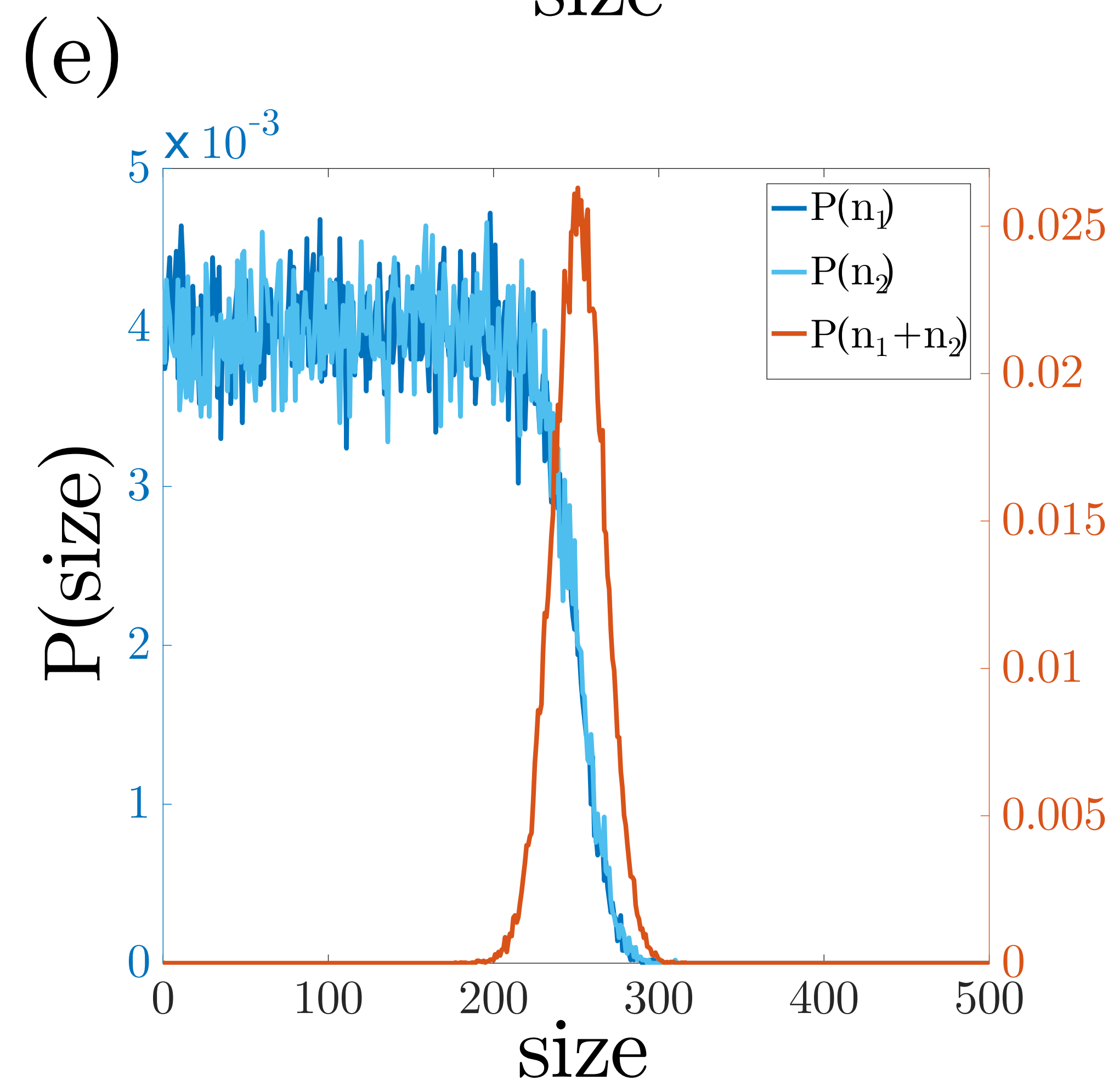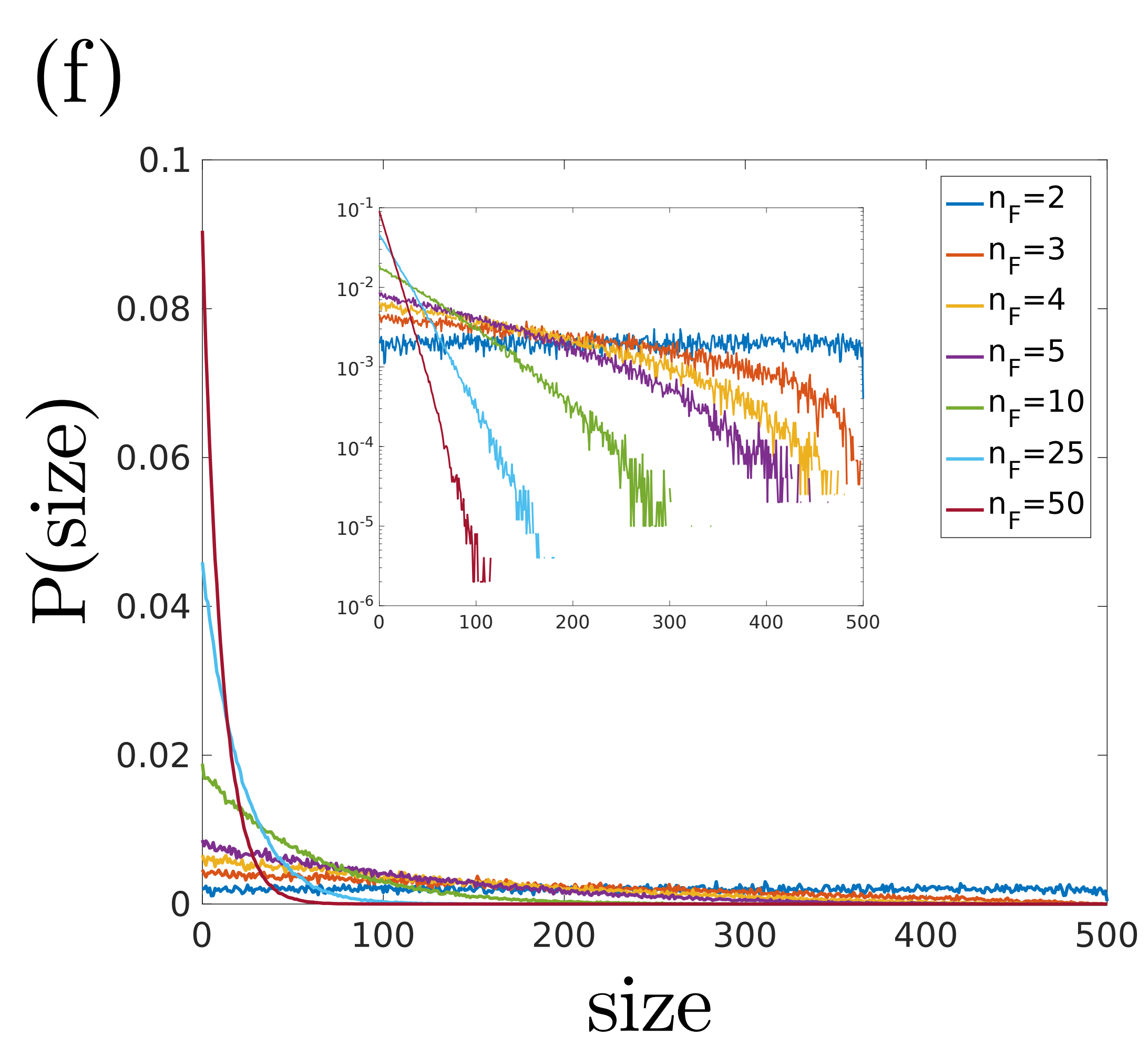

Supplement: S1 Fig — (A) For a single structure, the limiting pool model can provide robust size control. The structure reaches a steady state size after an initial period of fast growth. (B) The steady state size distribution shows a unimodal peaked distribution, characterising a well-defined mean size for the structure. (C) The limiting pool mechanism captures structure size scaling with cell size. (D-E) Limiting pool fails to control the individual size for two structures grown from a shared pool of subunits, giving rise to large anti-correlated fluctuations (D). The total size of the structures is a well controlled quantity, with temporal stability (D) and unimodal peaked distribution (E). The individual size distributions are almost uniform in a range of 0 to N − κ−1V (E). (F) For many structures, the individual size distributions converge to an exponential distribution—i.e., the standard deviation of size fluctuations are as large as the mean size, which is indicative of poor size control. (PDF) [file pcbi.1010253.s009.pdf]

● unstable    ● stable    ● saddle-point

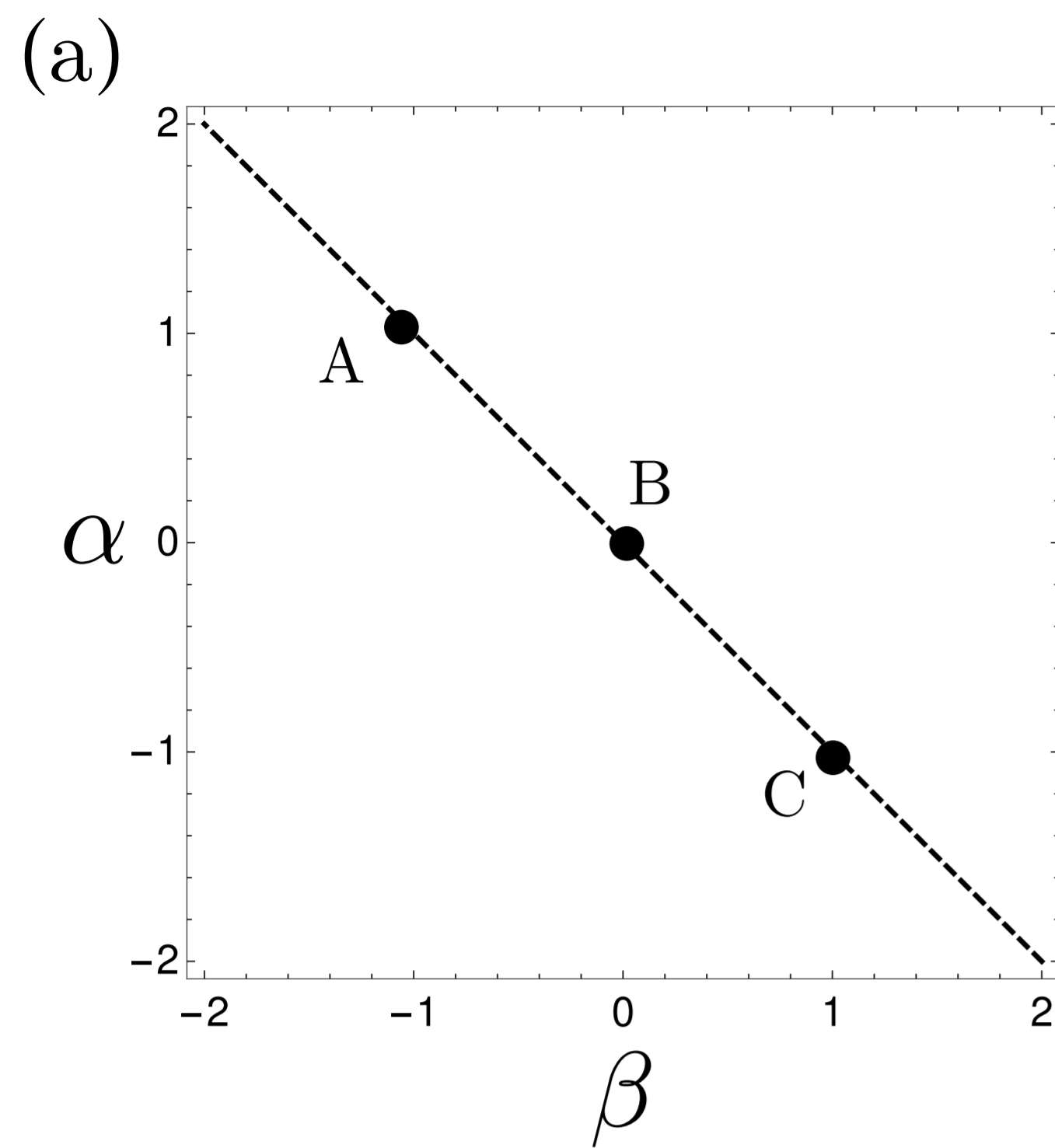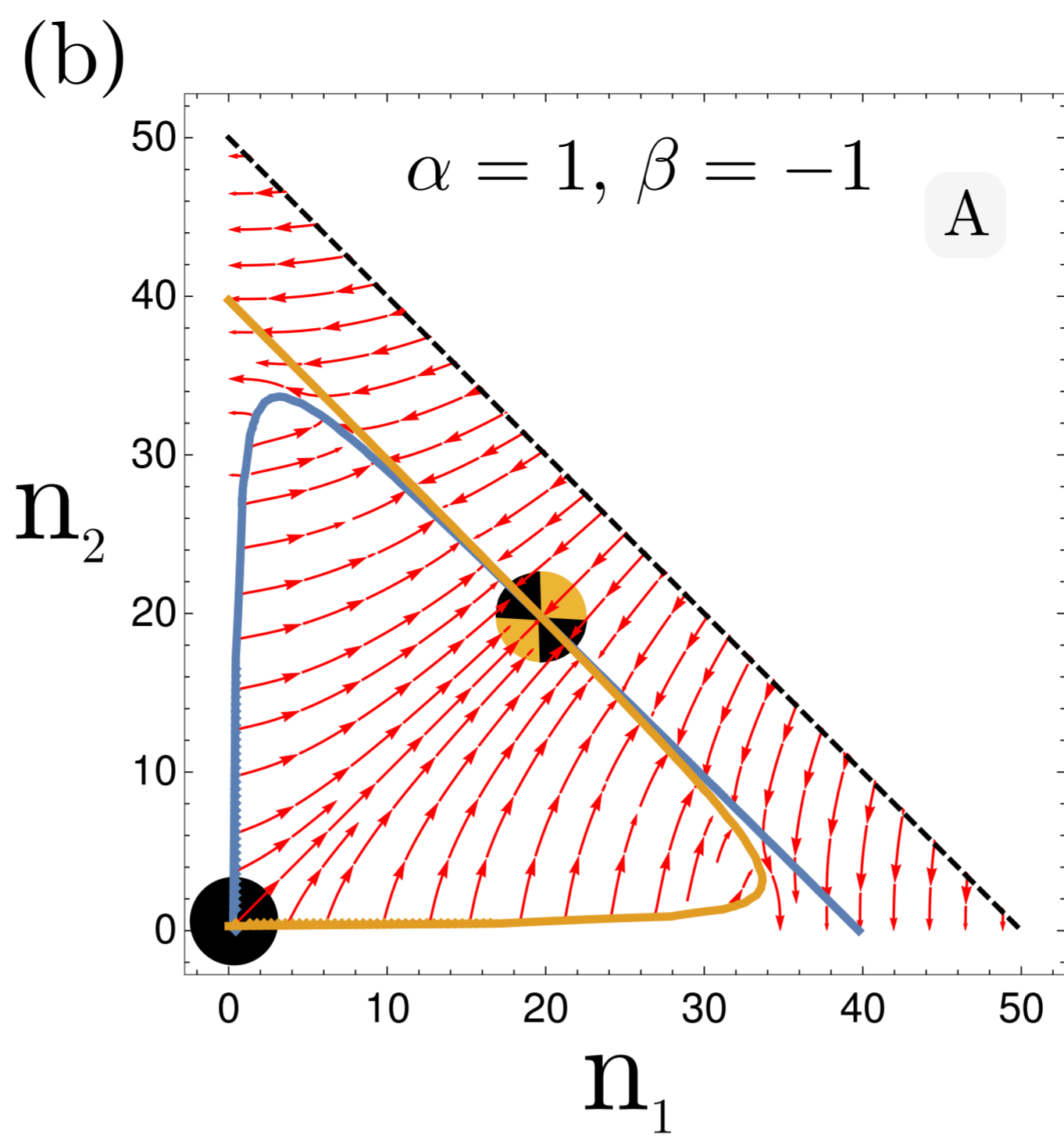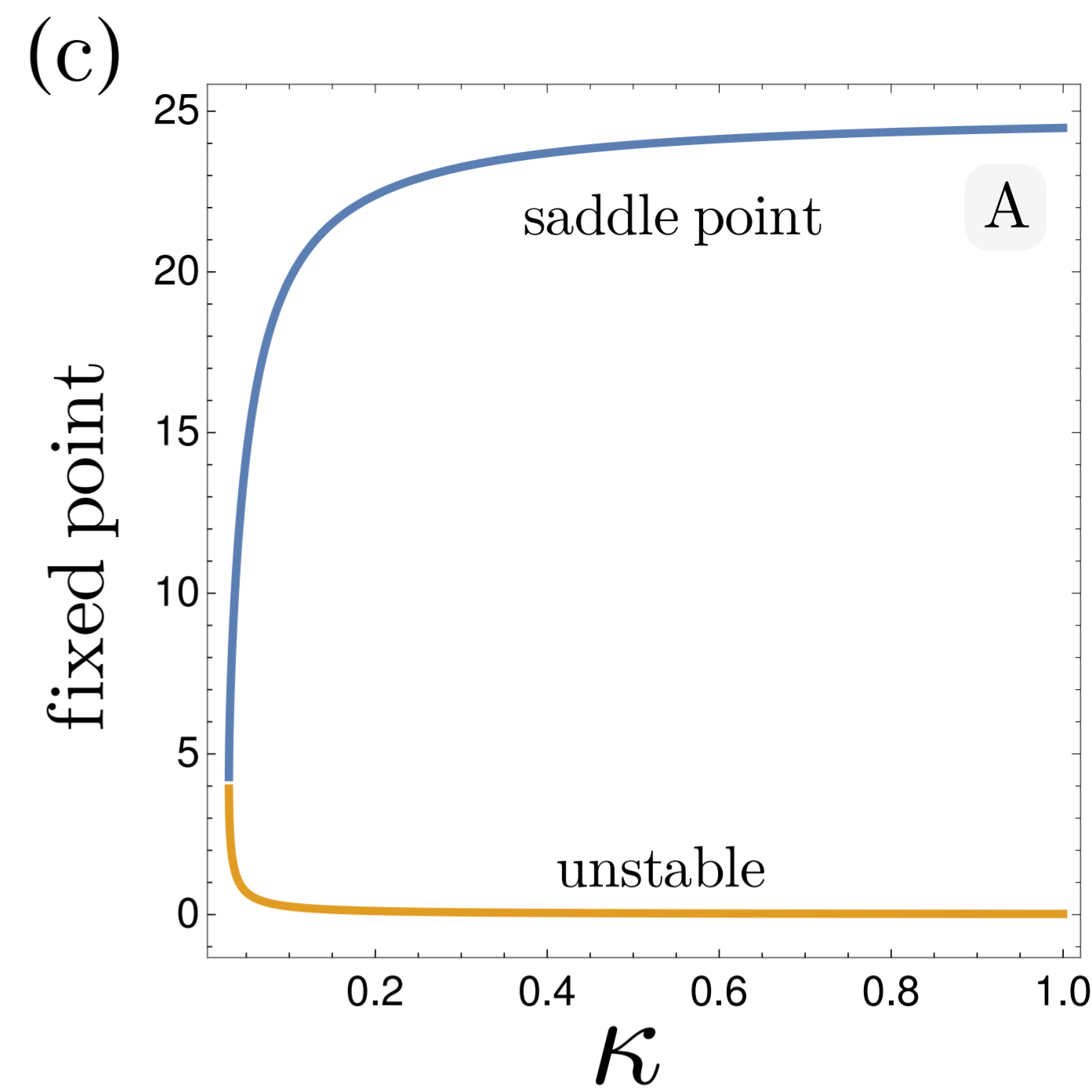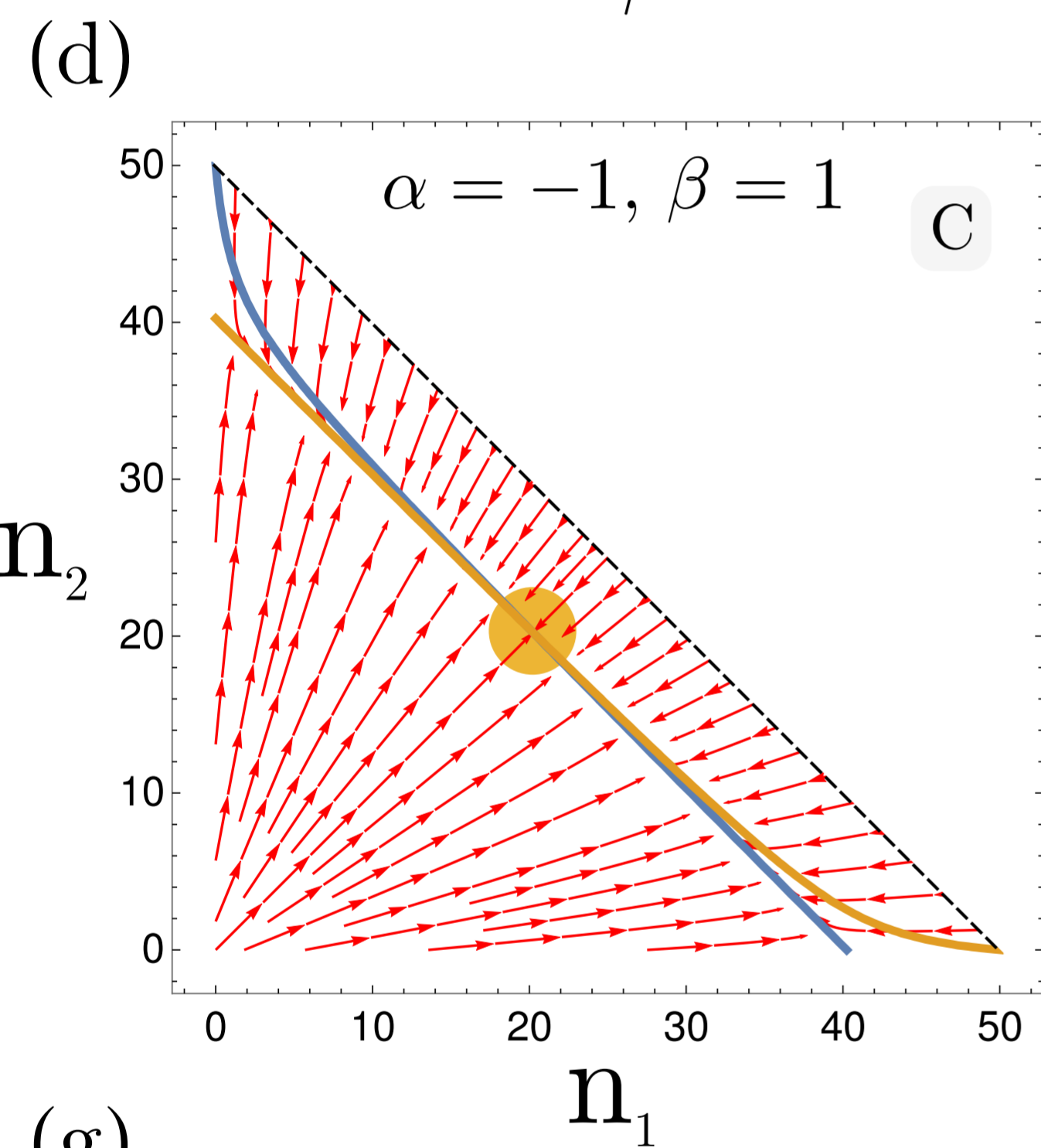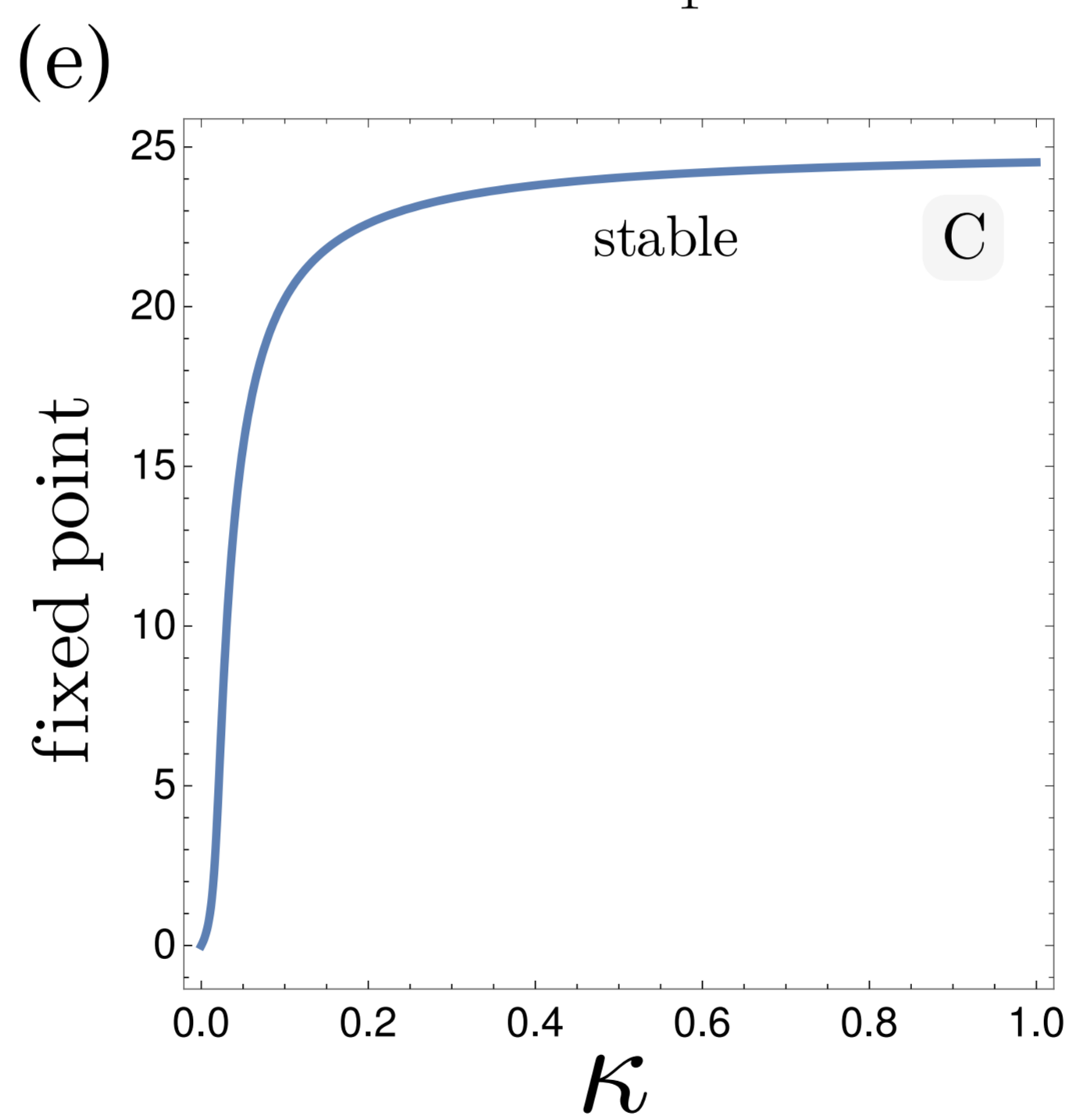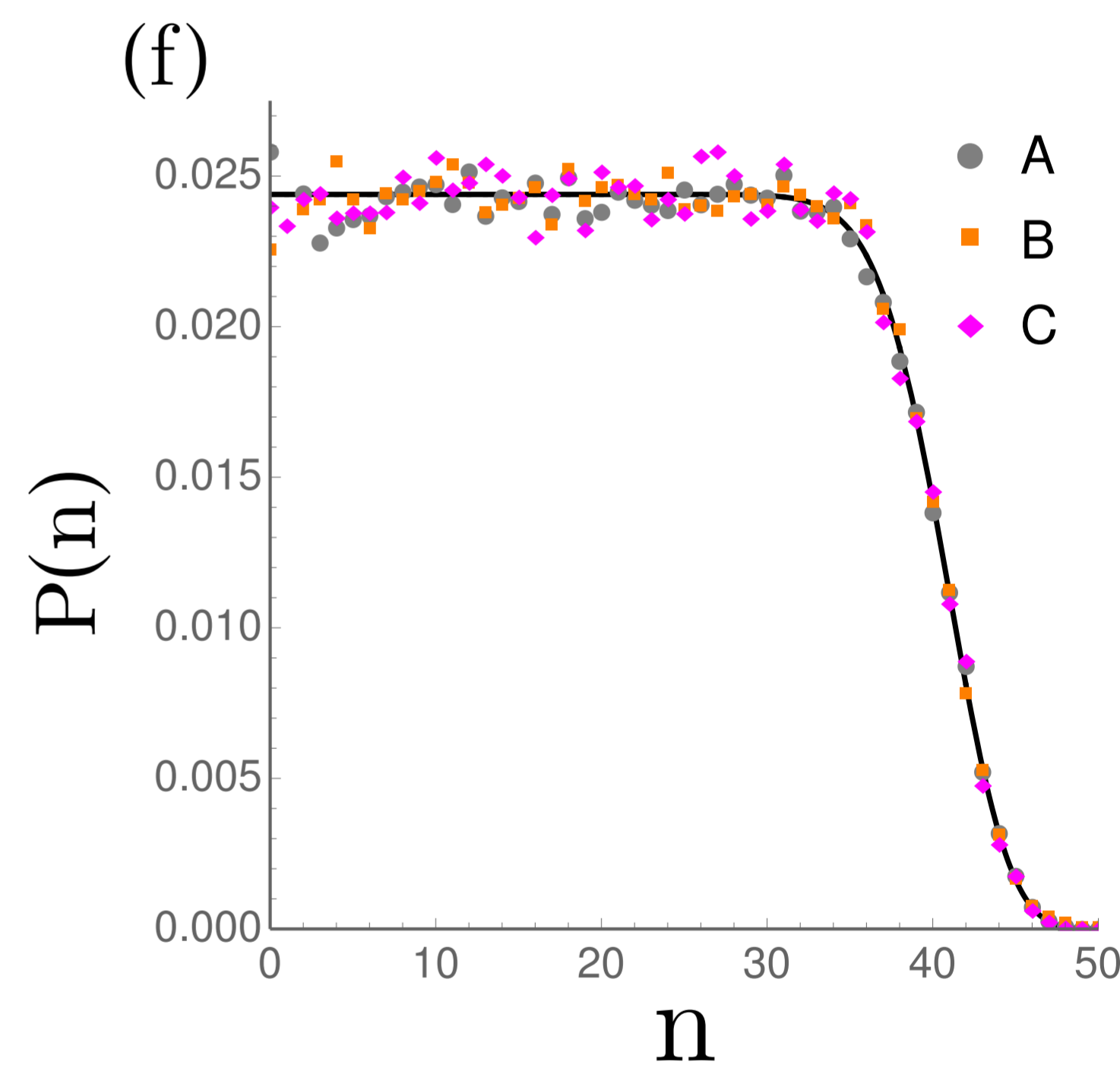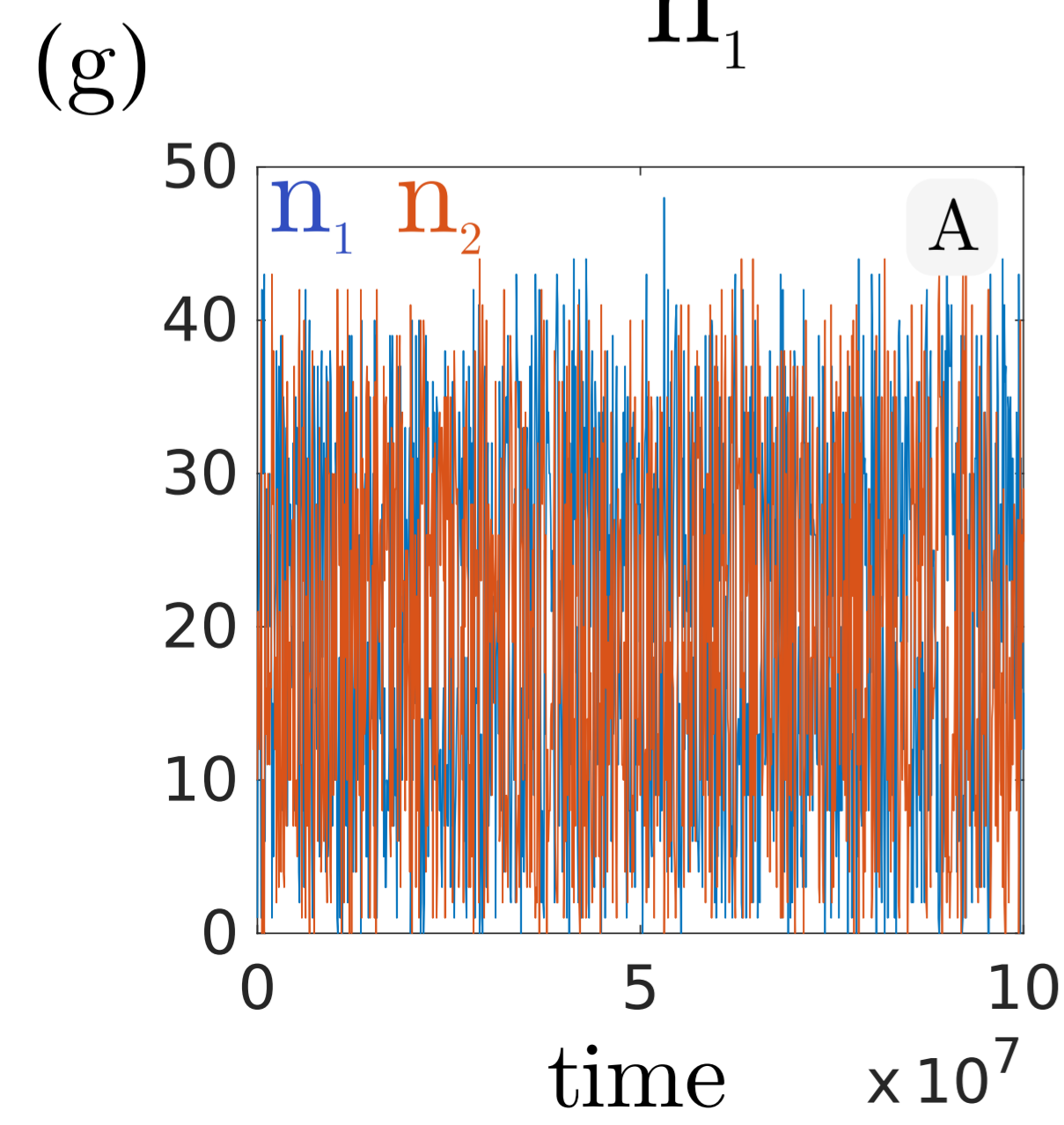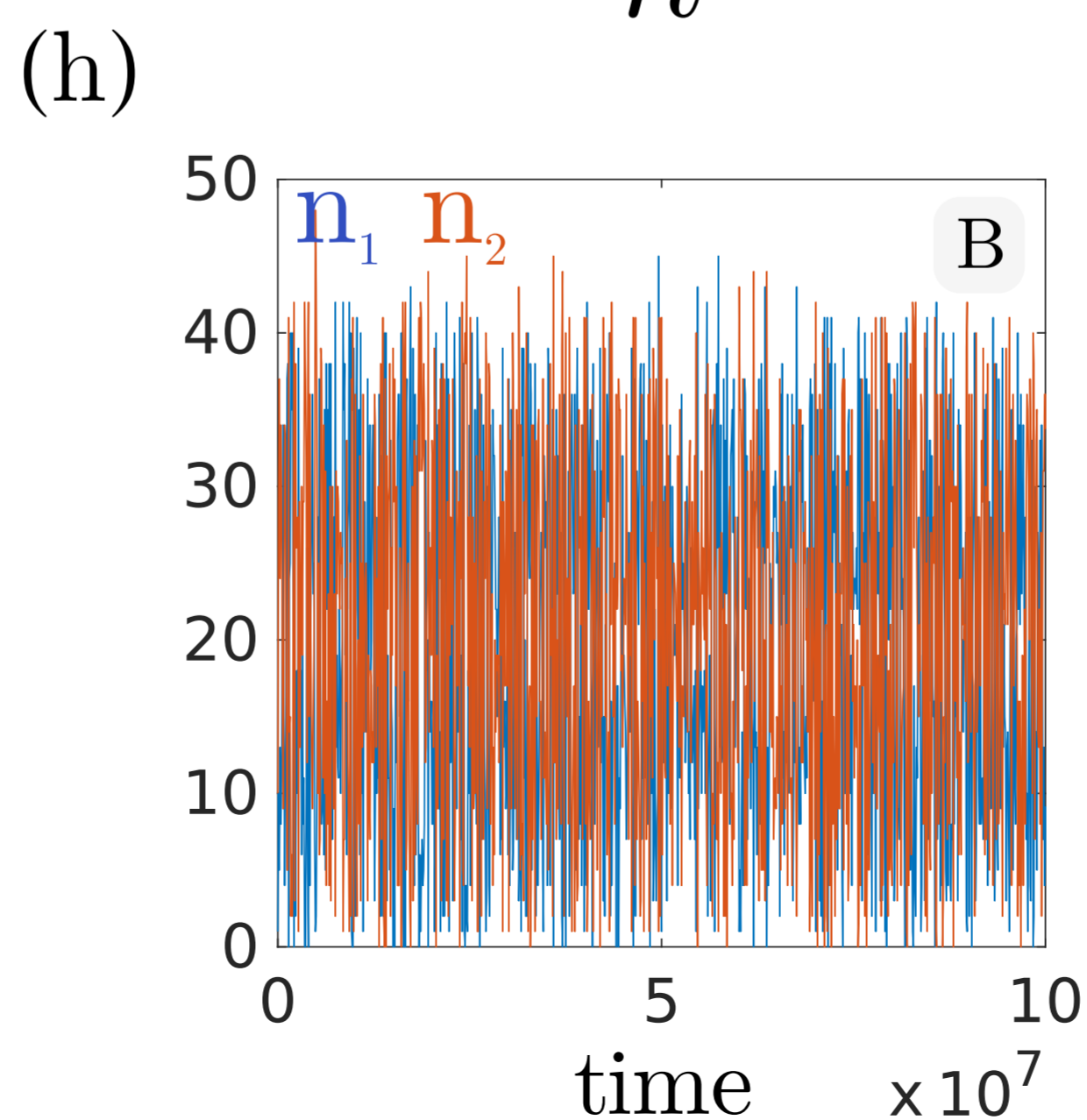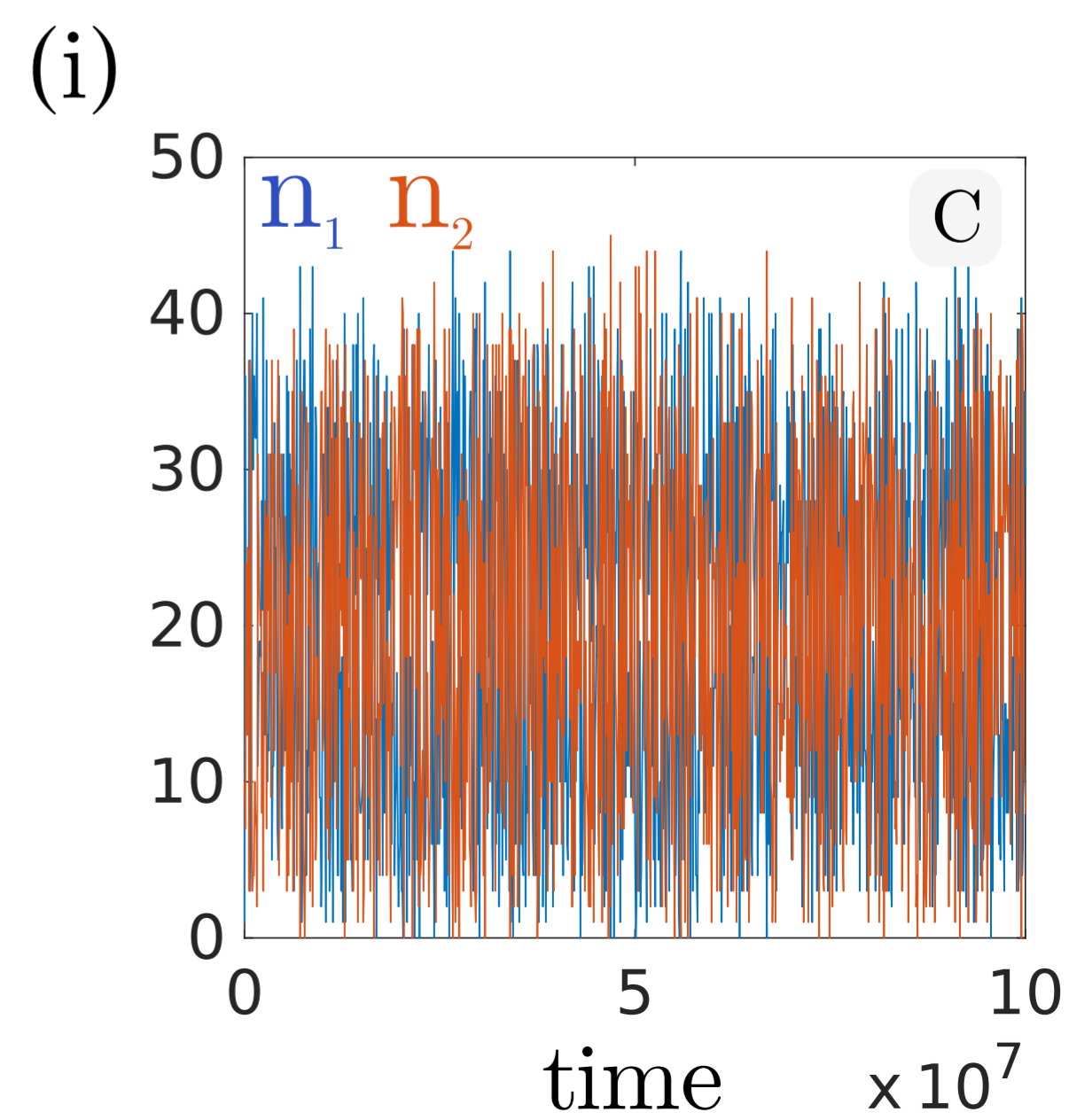

Supplement: S2 Fig — (A) We study the growth dynamics of structures A, B and C with coefficients α and β that lie on the line α + β = 0. B corresponds to the canonical limiting pool model. (B) For the case A, there are two fixed points and linear stability analysis indicates one to be a saddle node and the other to be an unstable fixed point near origin. (C) The fixed points in case A move away from each other as κ increases. (D) For case C, we find a single fixed point and linear stability indicates that the fixed point is stable. (E) The steady-state size given by the stable fixed point increases with κ, saturating at high κ values. The blue (n˙1=0) and yellow (n˙2=0) lines (in panels B and D) are the nullclines and the red arrows represent the flow in the n1 − n2 phase plane. (f) Structure size distributions in the three cases in (A), given by the solution chemical master equation solution (solid line) and stochastic simulations (points). (G-I) Structure size dynamics obtained from stochastic growth simulations show large anti-correlated fluctuations (in all three cases), leading to a failure in size control. Parameters: N = 50 and κ = 0.1. (PDF) [file pcbi.1010253.s010.pdf]

● unstable    ● stable    ● saddle-point

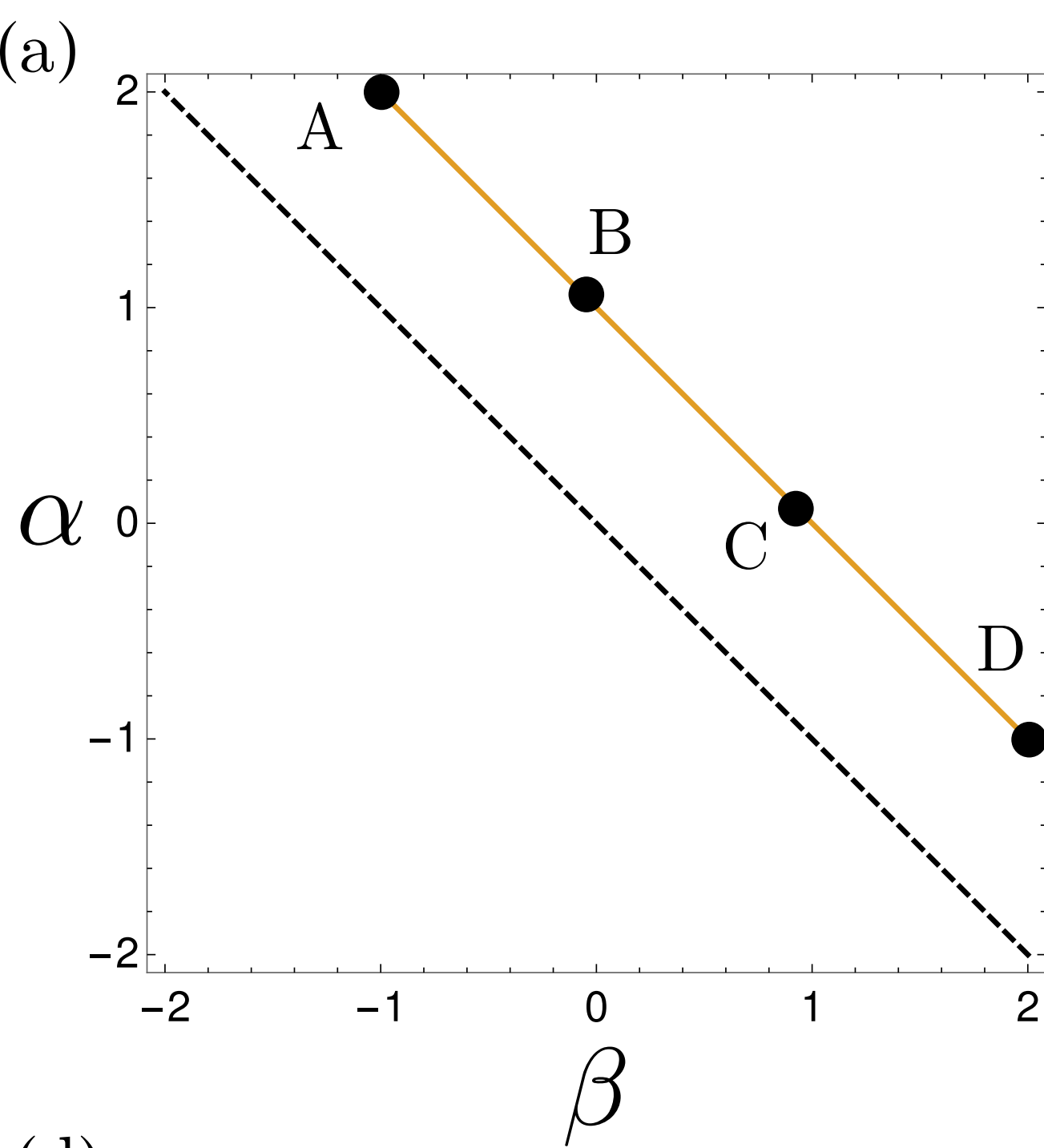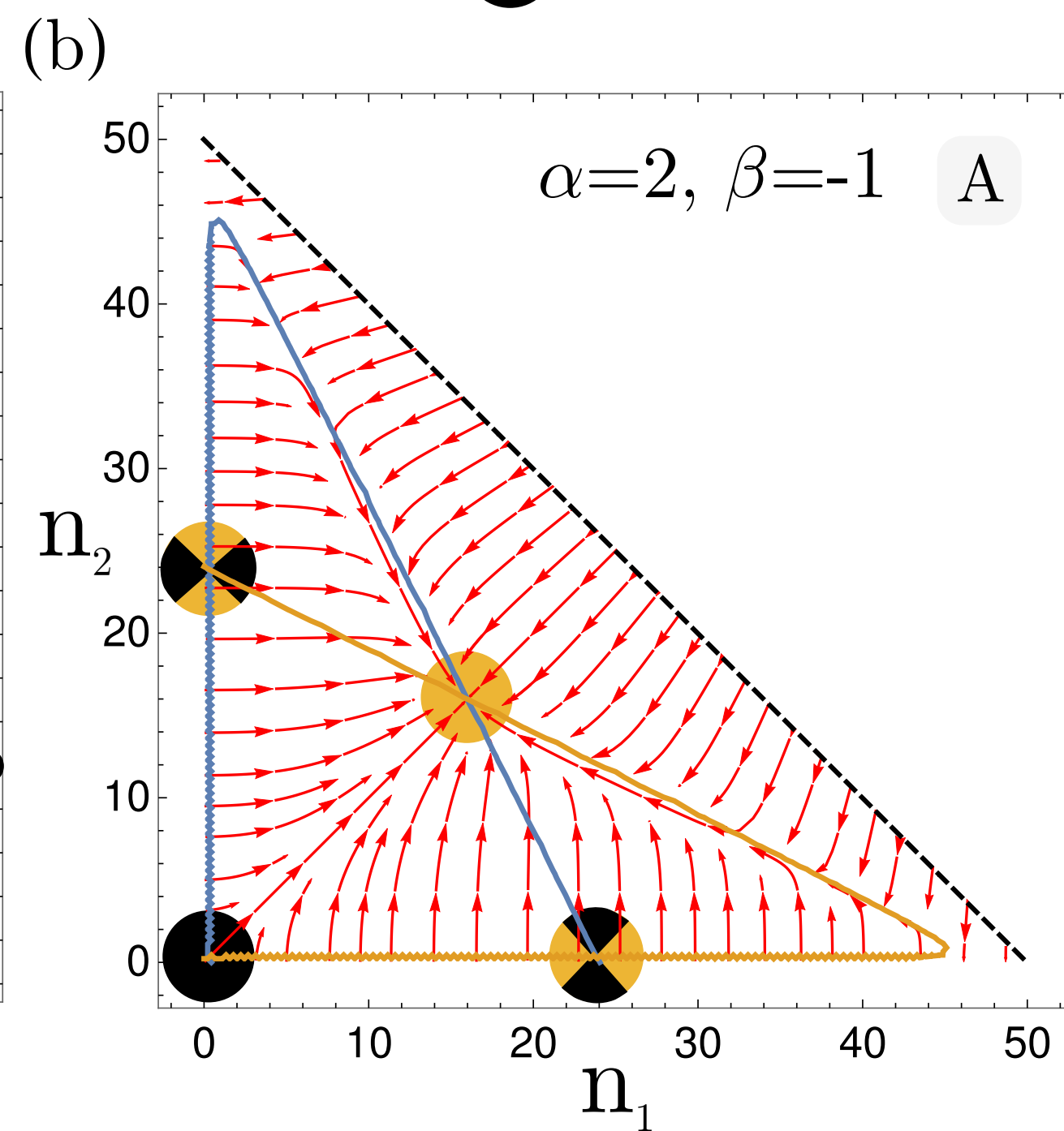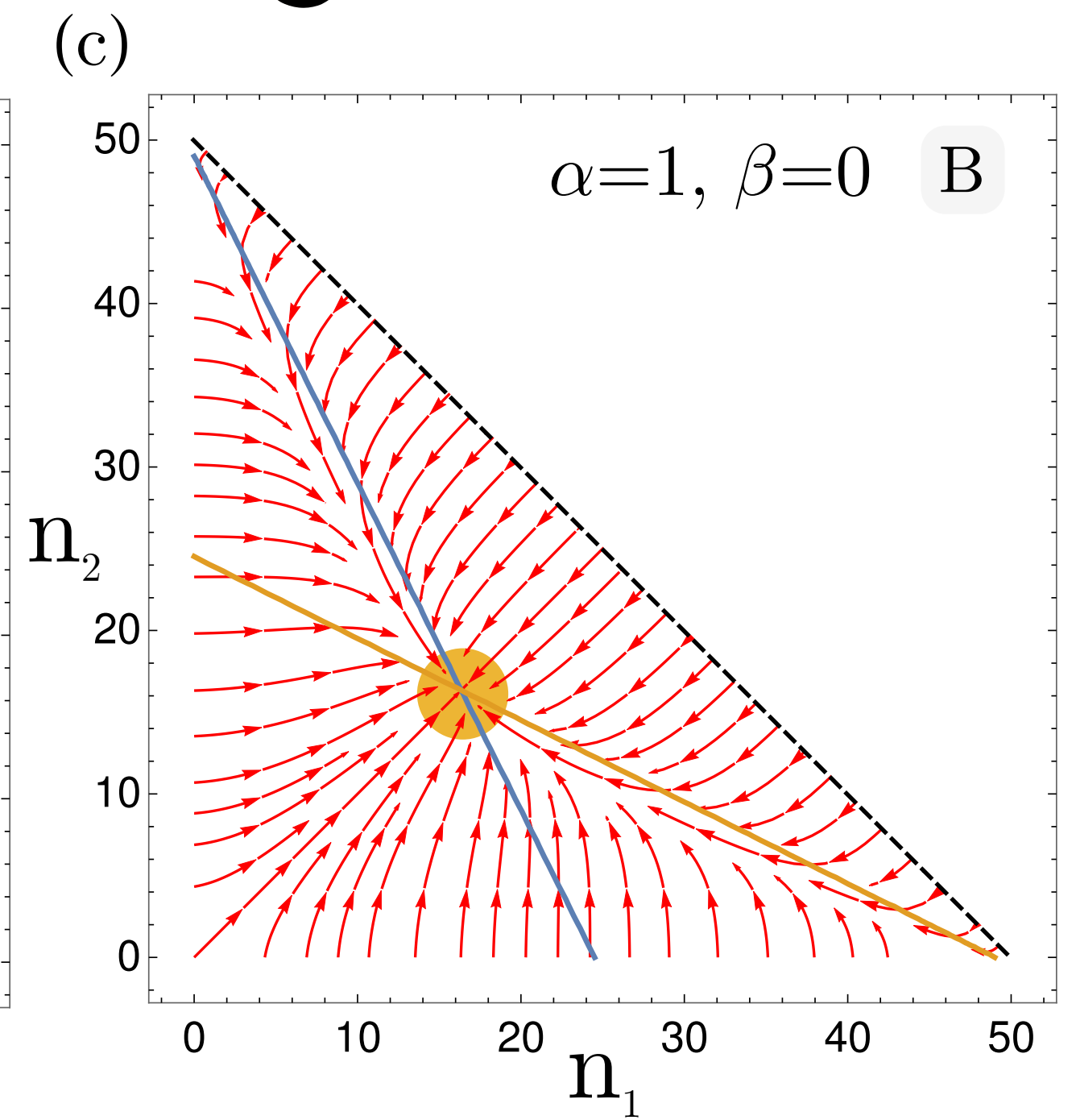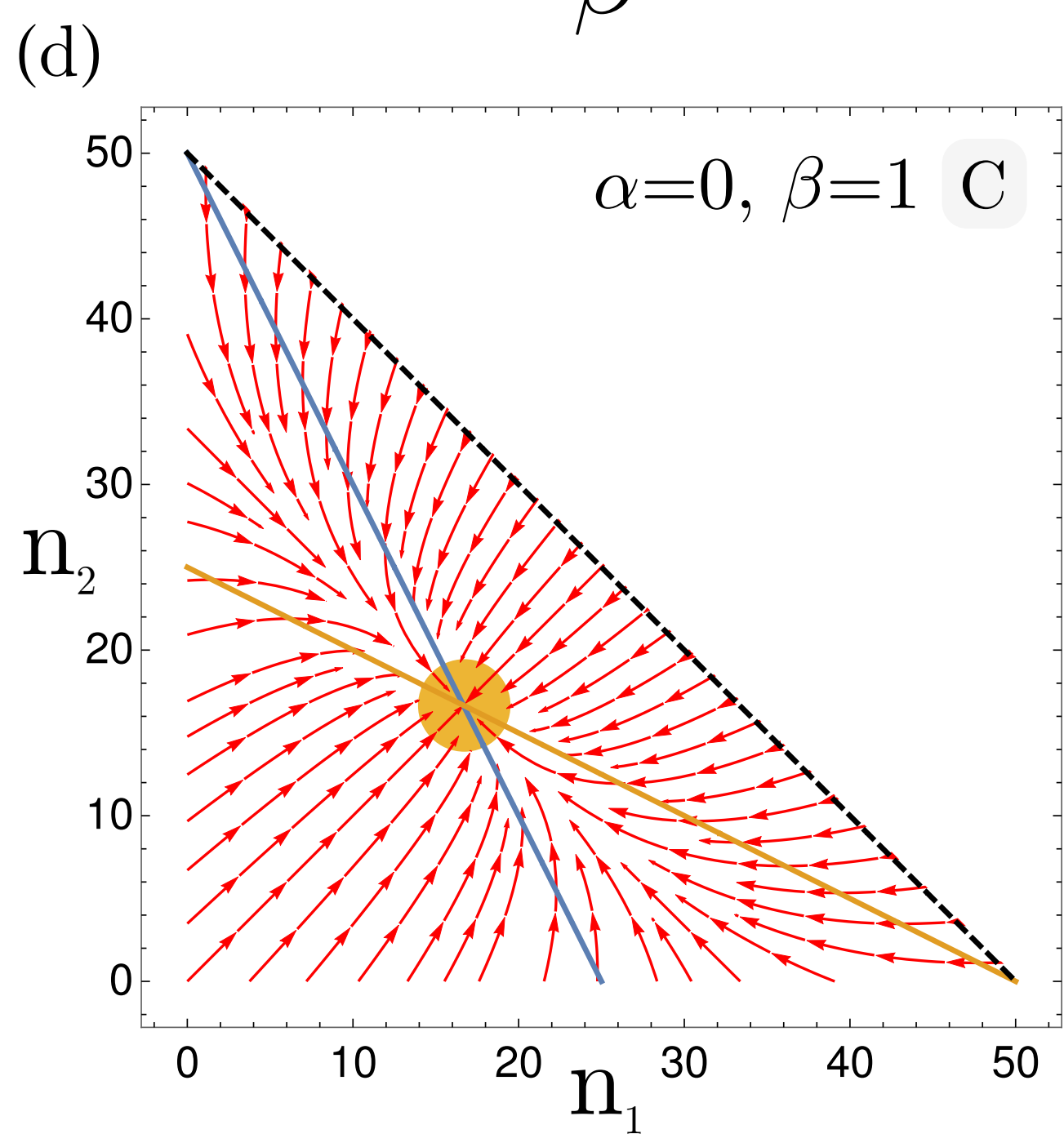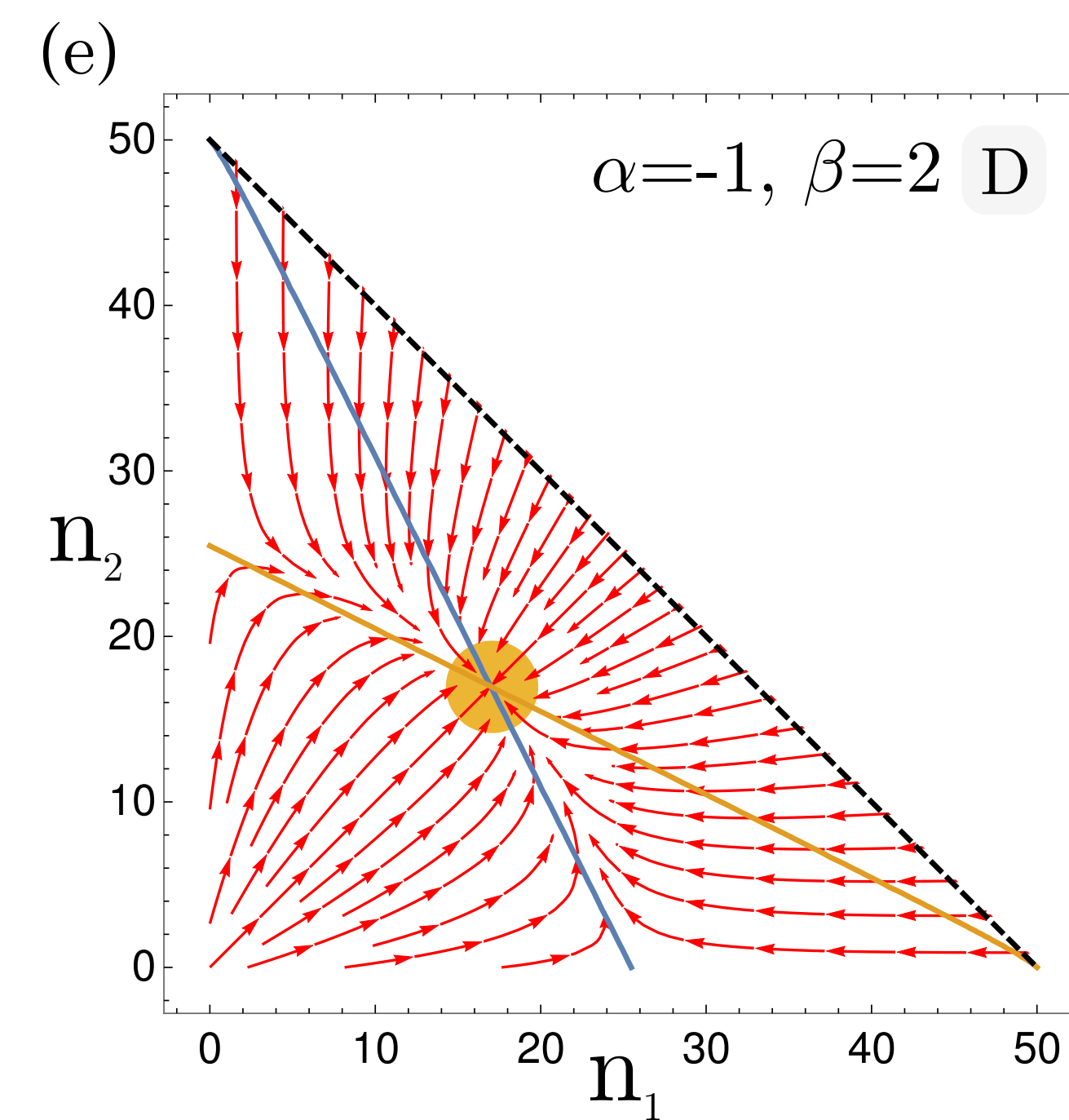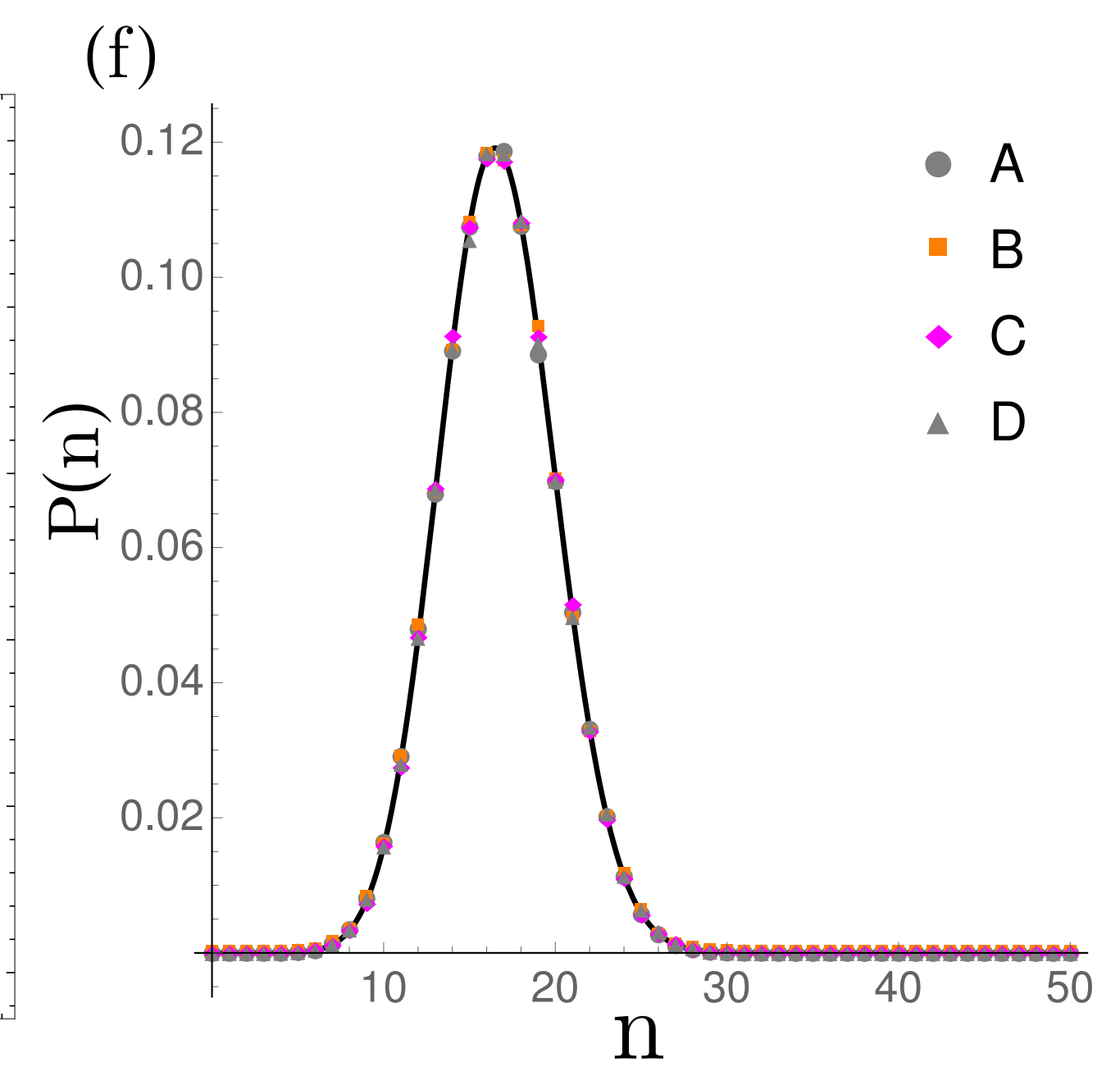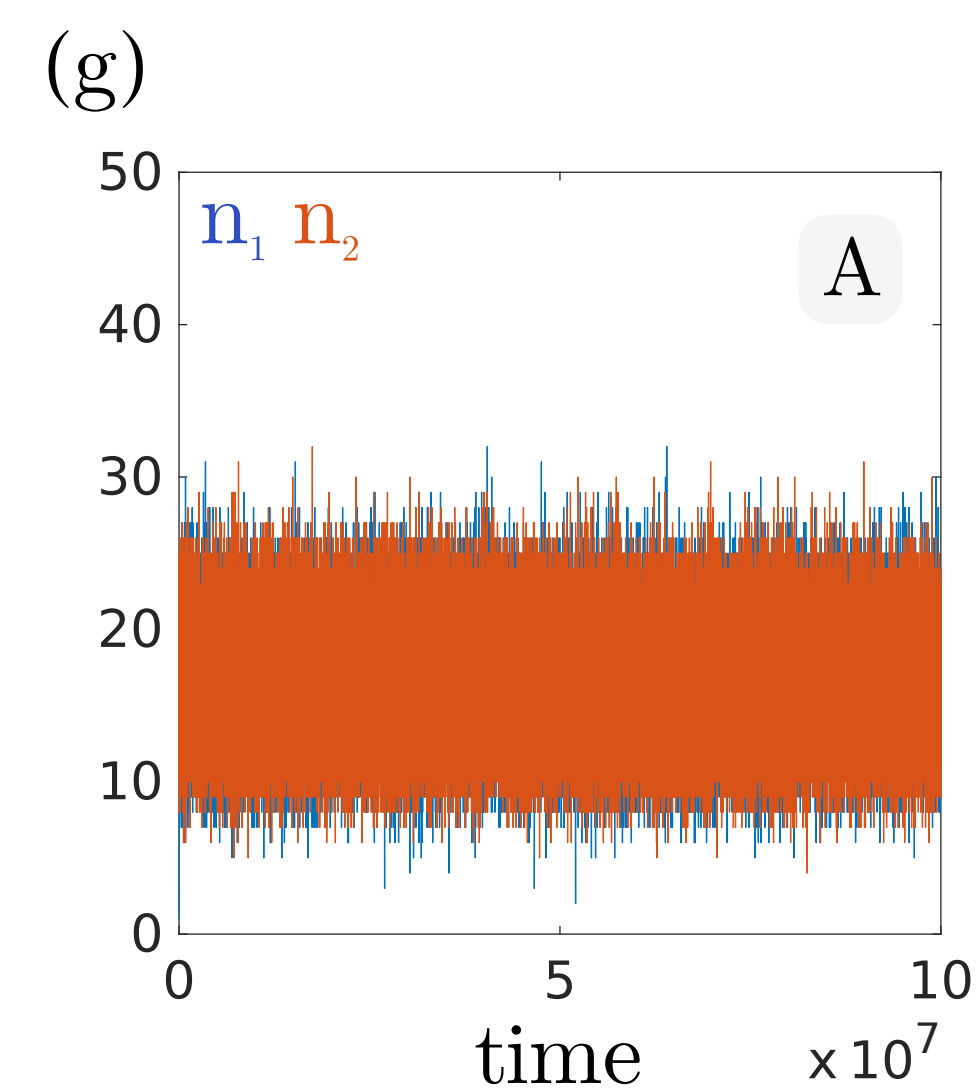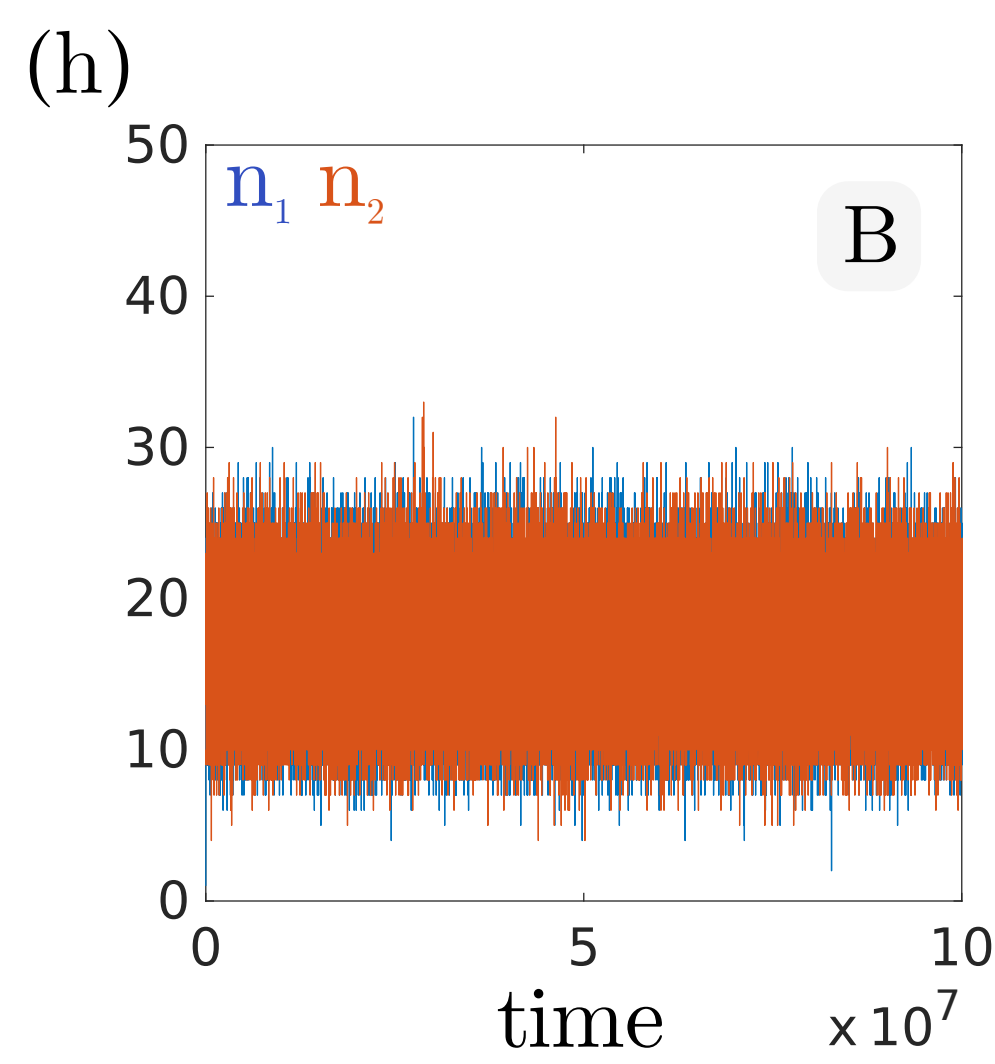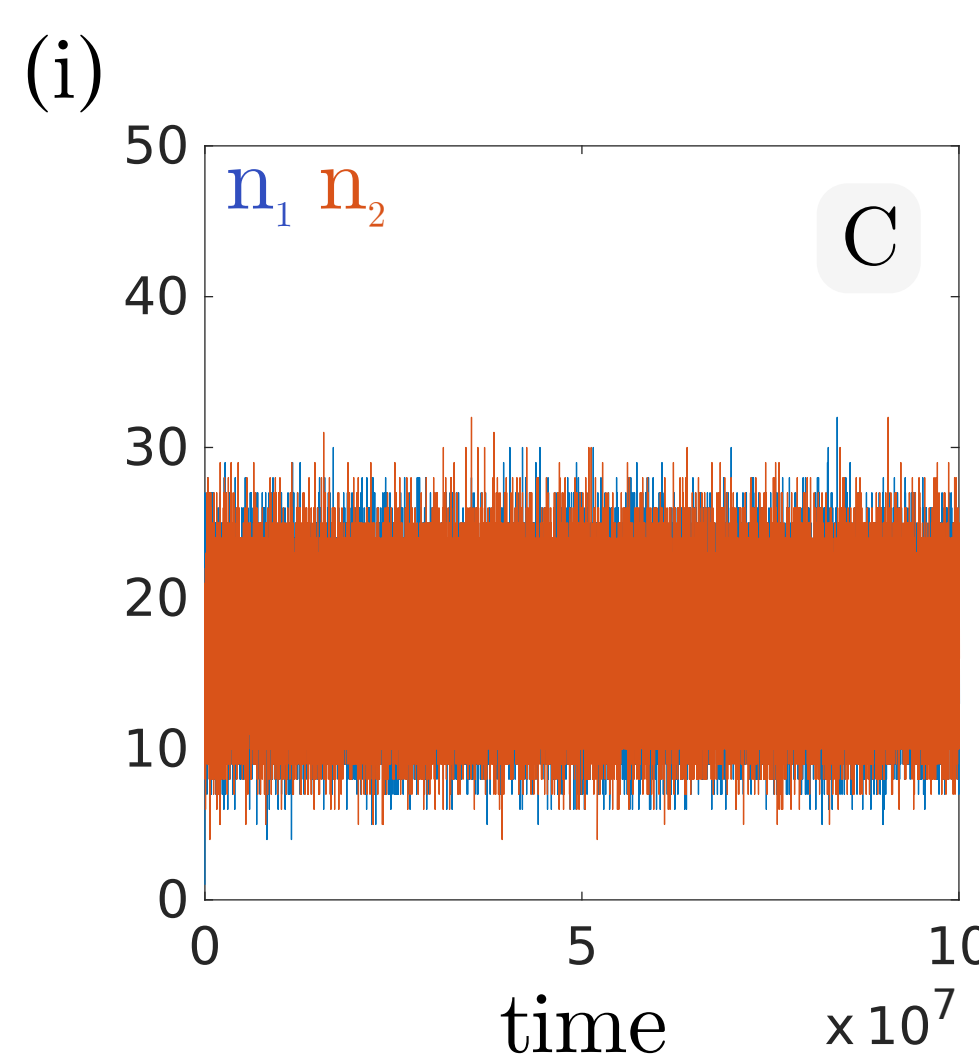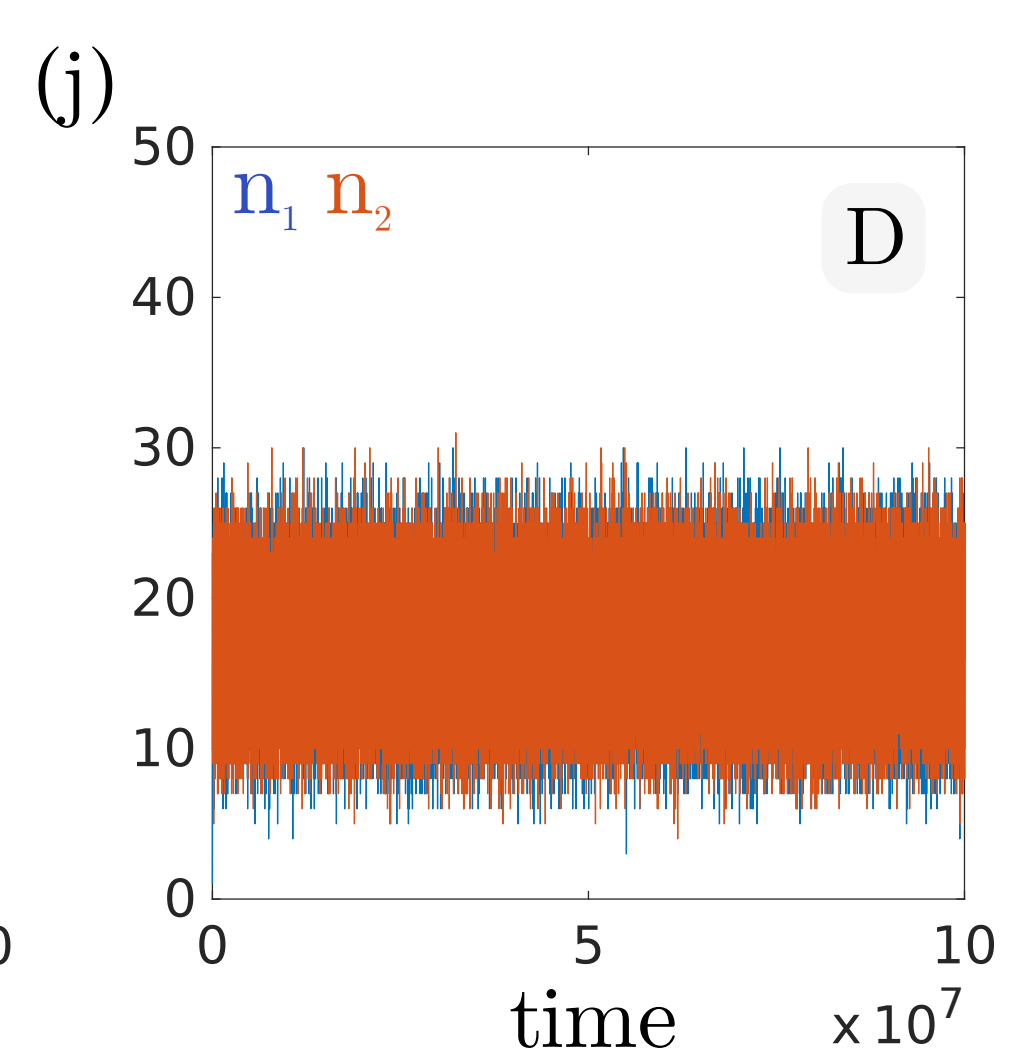

Supplement: S3 Fig — (A) We study the dynamics of structure growth at four points A, B, C and D on the α + β = 1 line which lies in the regime where there is negative feedback control of structure growth. (B) For the case A we find four fixed points—one unstable node, one stable node and two saddle nodes inferred from linear stability. (C-E) In all three cases B, C and D we find a single stable node from linear stability. The blue (n˙1=0) and the yellow (n˙2=0) lines (in panels B-E) are the nullclines and the red arrows represent the flow in the n1−n2 phase space. (F) The chemical master equation solution (black line) and stochastic simulations (points) predicts the same size distributions in all four cases, with a well defined mean value and comparatively small standard deviation, reflecting size control. (G-J) The temporal dynamics from stochastic simulations show well defined mean size at all times. Despite the difference in individual α, β values the statistical properties of the size dynamics are the same in all the four cases. (PDF) [file pcbi.1010253.s011.pdf]

○ stable    ● unstable    ● stable

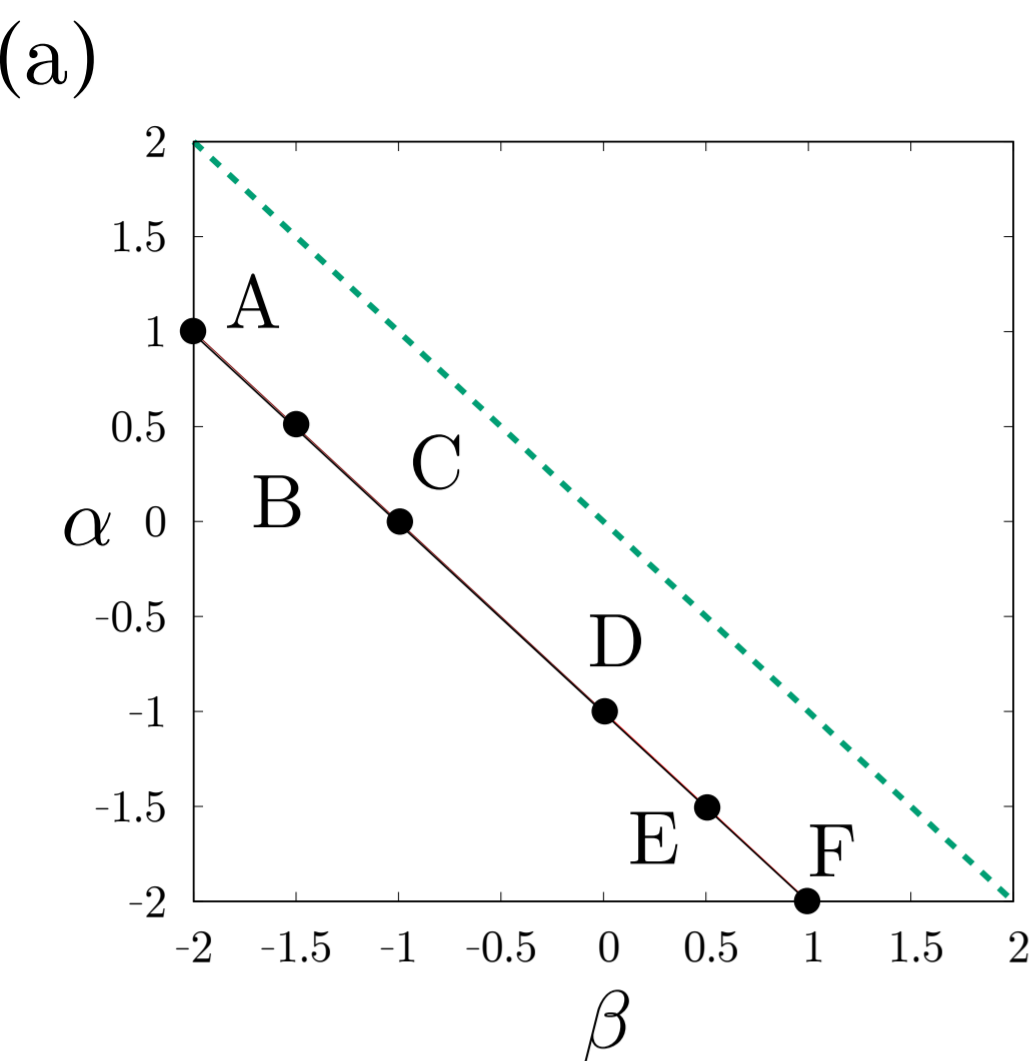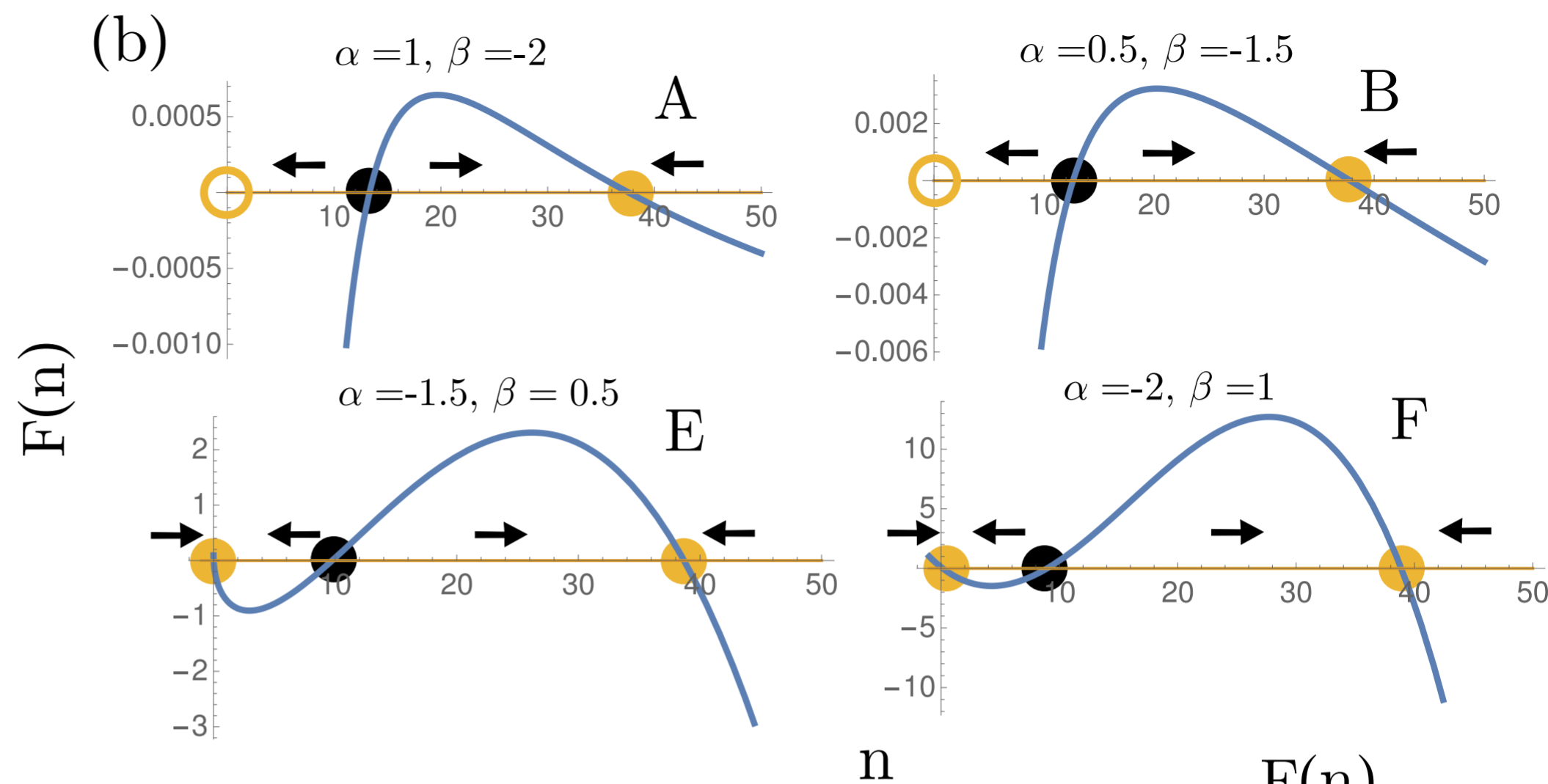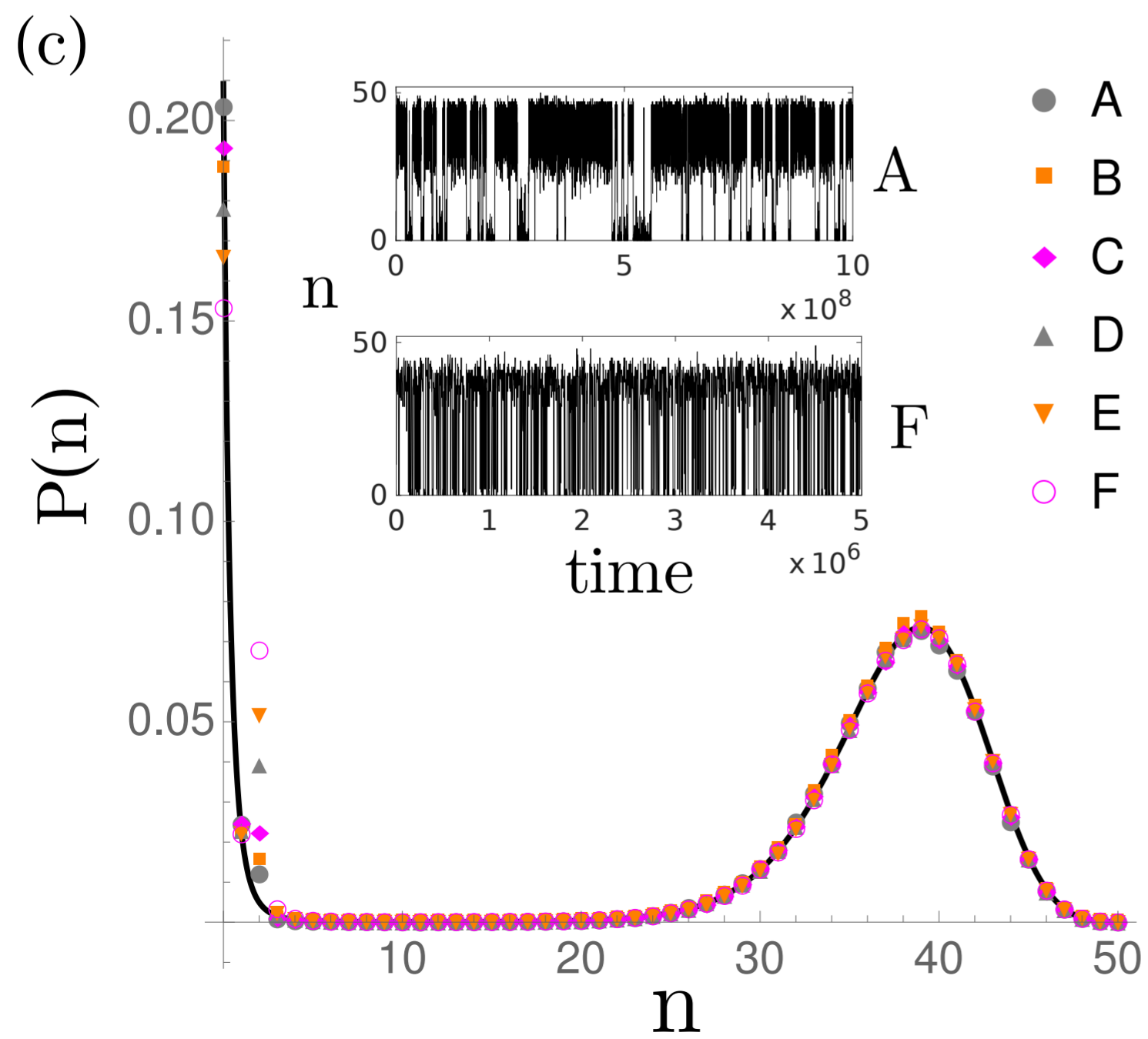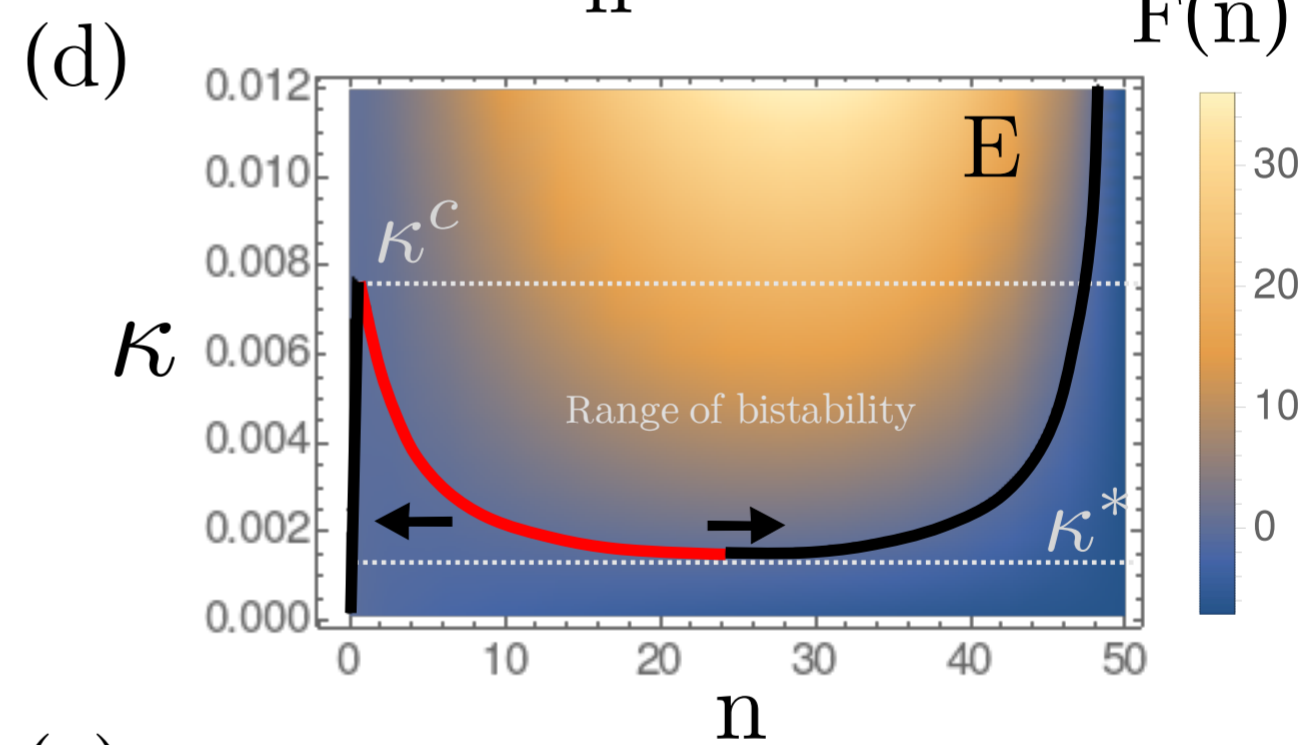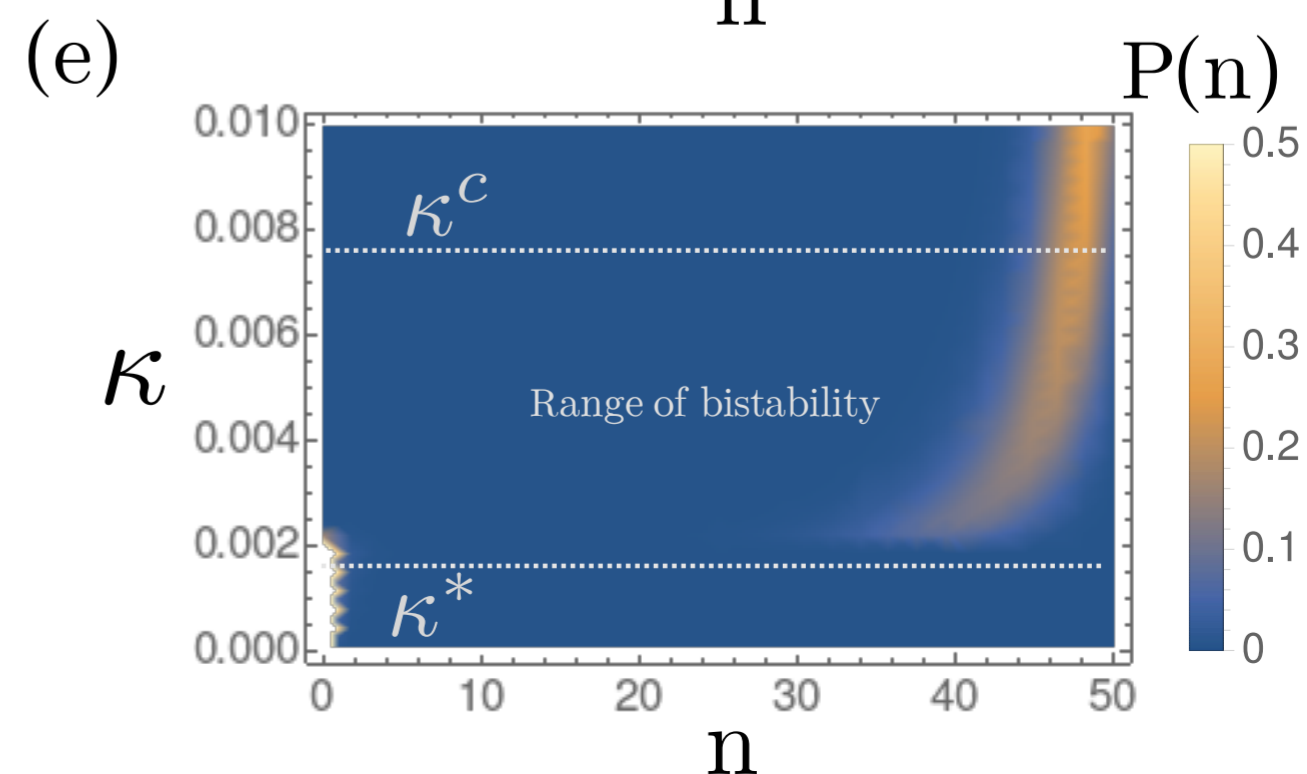

Supplement: S4 Fig — (A) We study the size dynamics of a growing structure at six different parameter regimes, namely the points A, B, C, D, E and F lying on the line α + β = −1. In this regime, there is a size-dependent positive feedback on the growth of the structure. (B) Stability diagram showing growth rate n˙=F(n) vs structure size n in the deterministic model. Top row: For β < 0 there are only two fixed points, one stable and the other unstable, showing no apparent presence of bistability. The second stable fixed point (open circle) is obtained from treating the divergence at boundary (n = 0) and considering Koff(0) = 0. Bottom row: when β > 0, there are three fixed points—two stable and one unstable, and thus the system is bistable. (C) Structure size distribution obtained from solution to the chemical master equation solution (solid line), and from stochastic simulations (points), showing bimodality of size distributions in all six parameter regimes. Inset: Temporal evolution of structure size in cases A and F, illustrating that residence times in the two stable states is dependent on the α and β values. (D) Evolution of the fixed points (solid lines) as a function of growth rate κ showing that for a single structure the bistability is only present in the range κ* < κ < κc. The black and red lines indicate the position of the stable and unstable fixed points, respectively. Heatmap shows dn/dt. (E) Steady-state probability distribution P(n), as a function of κ and n. For all calculations N = 50 and κ = 0.0022 (except in e panel) was taken. (PDF) [file pcbi.1010253.s012.pdf]

unstable    
  stable    
  unstable    
  stable    
  saddle-point

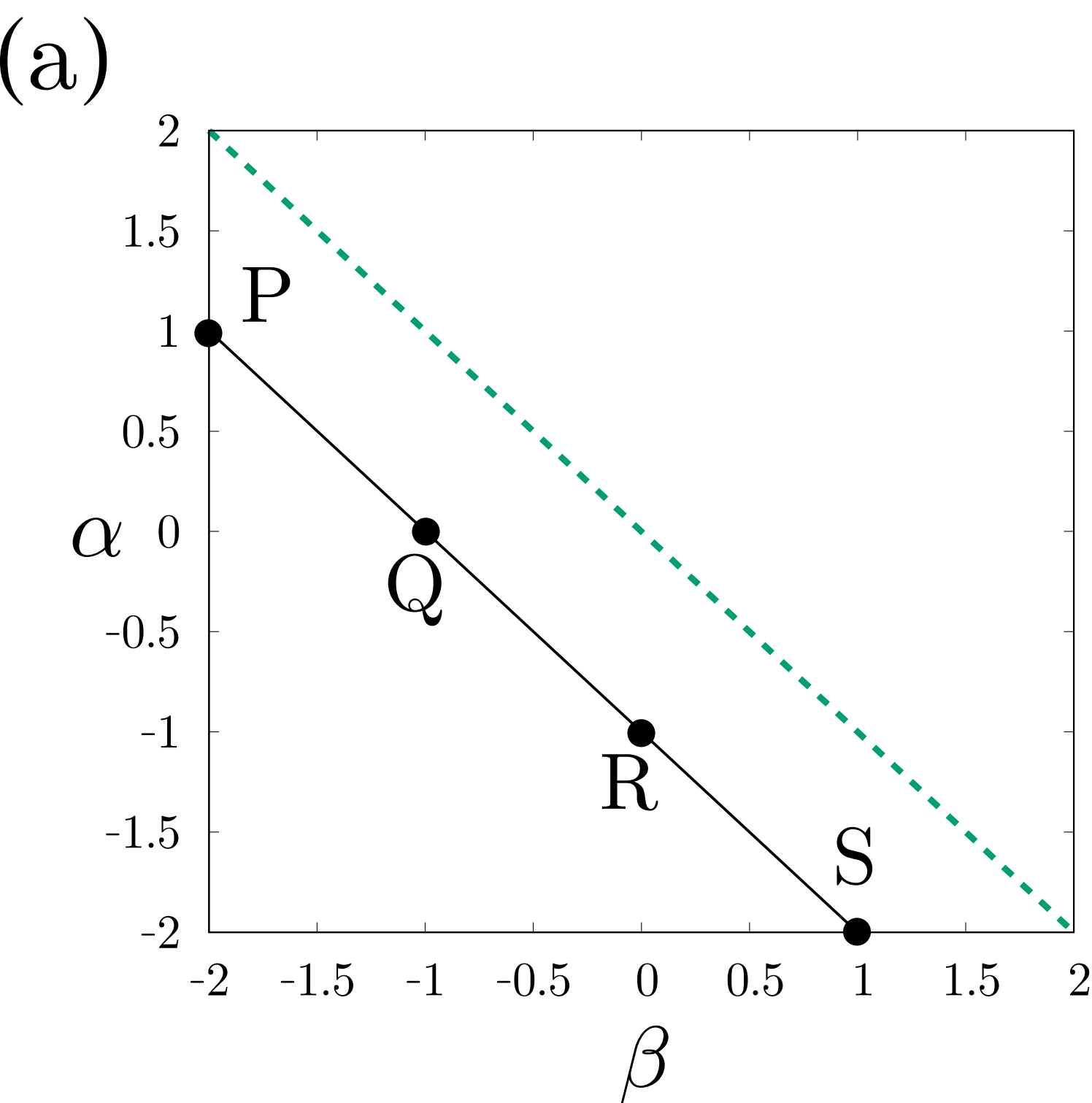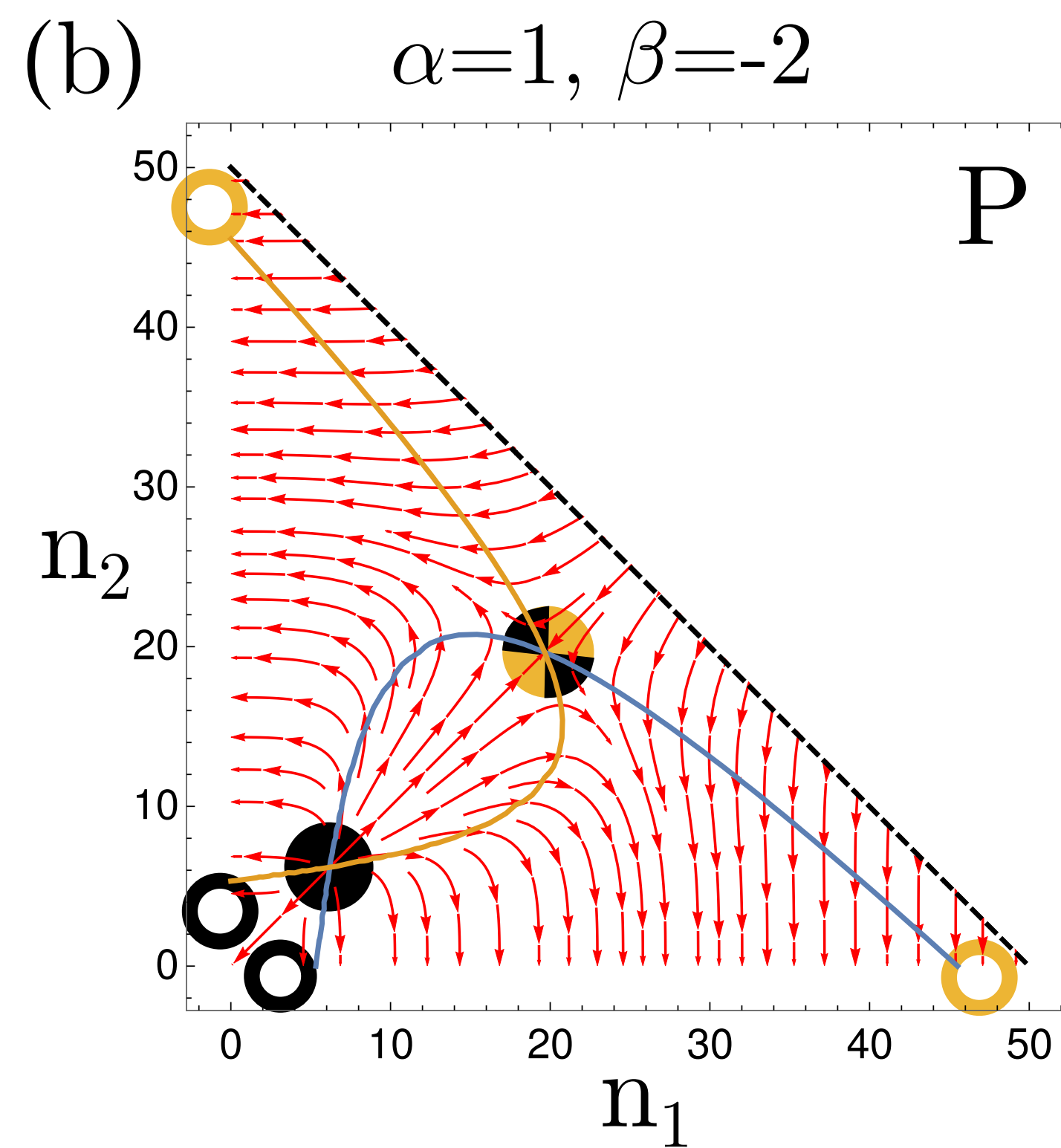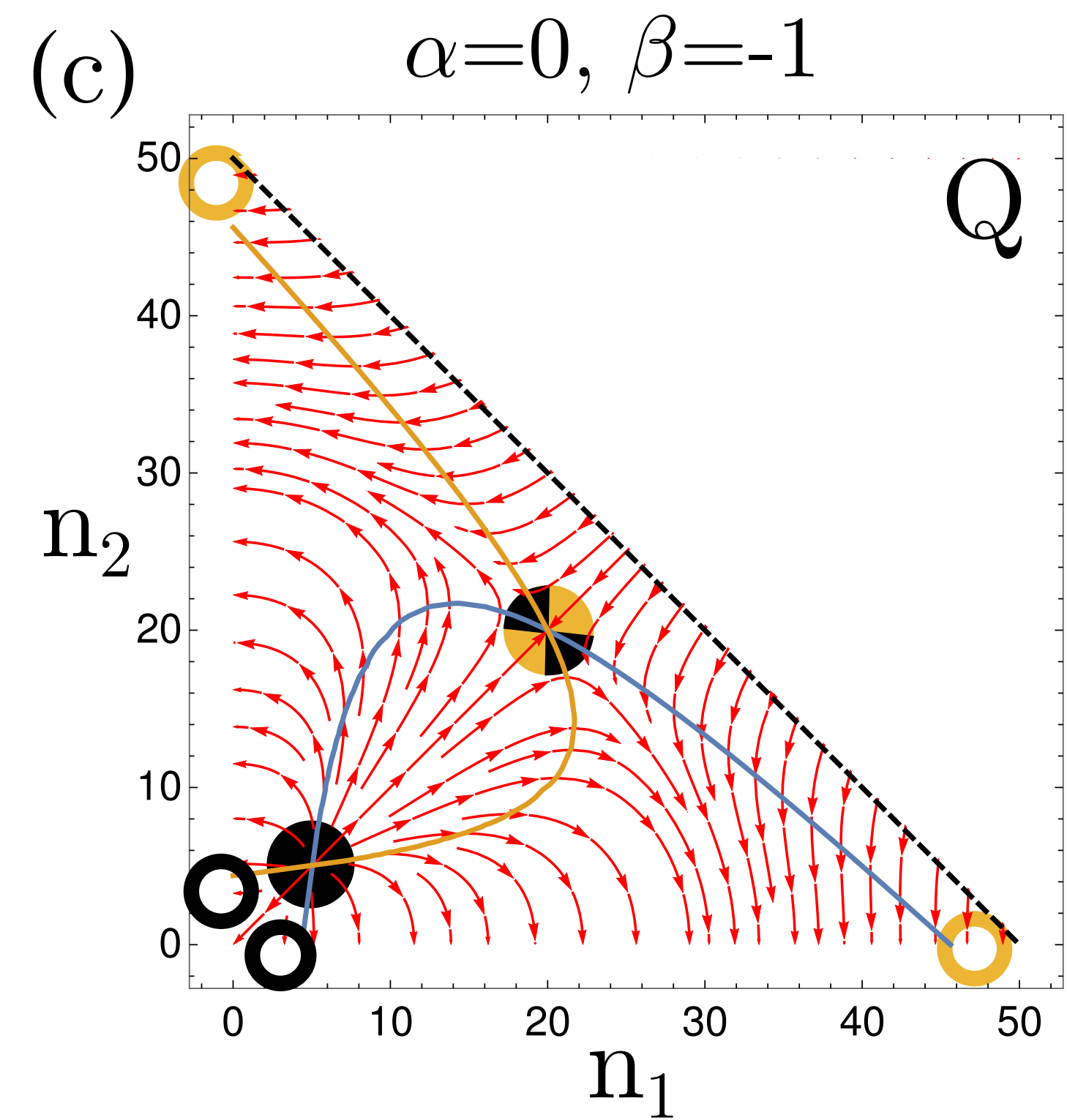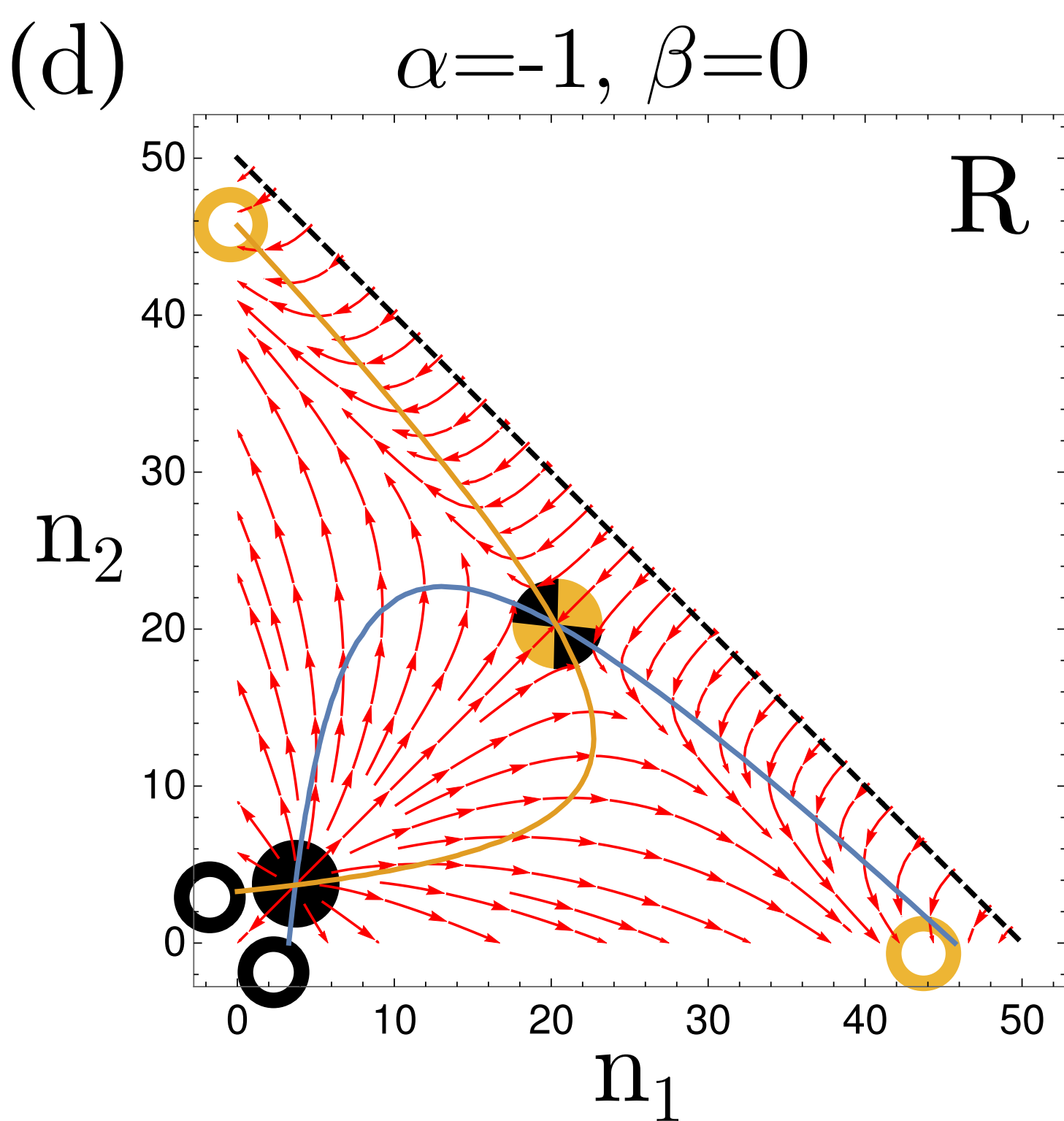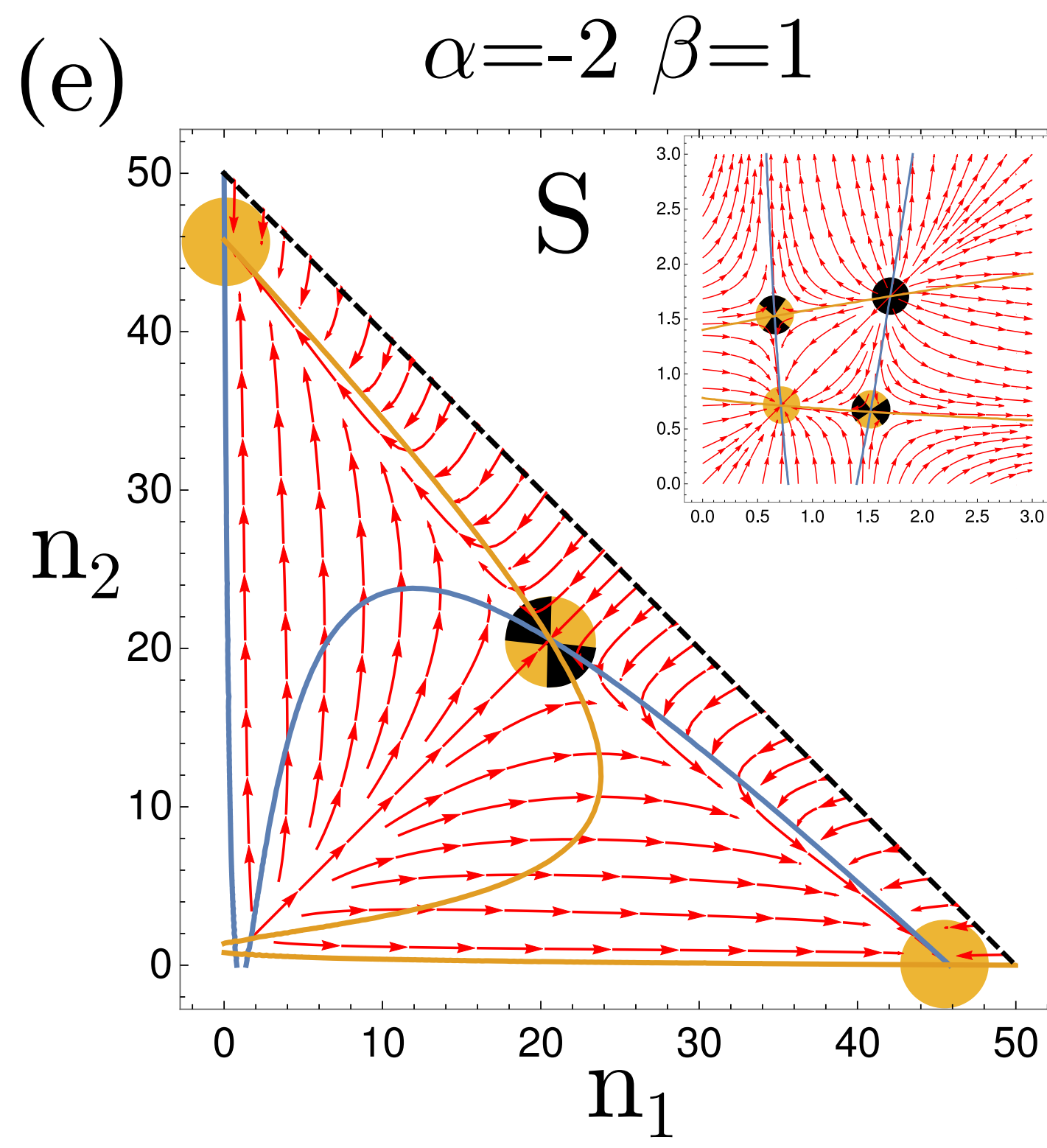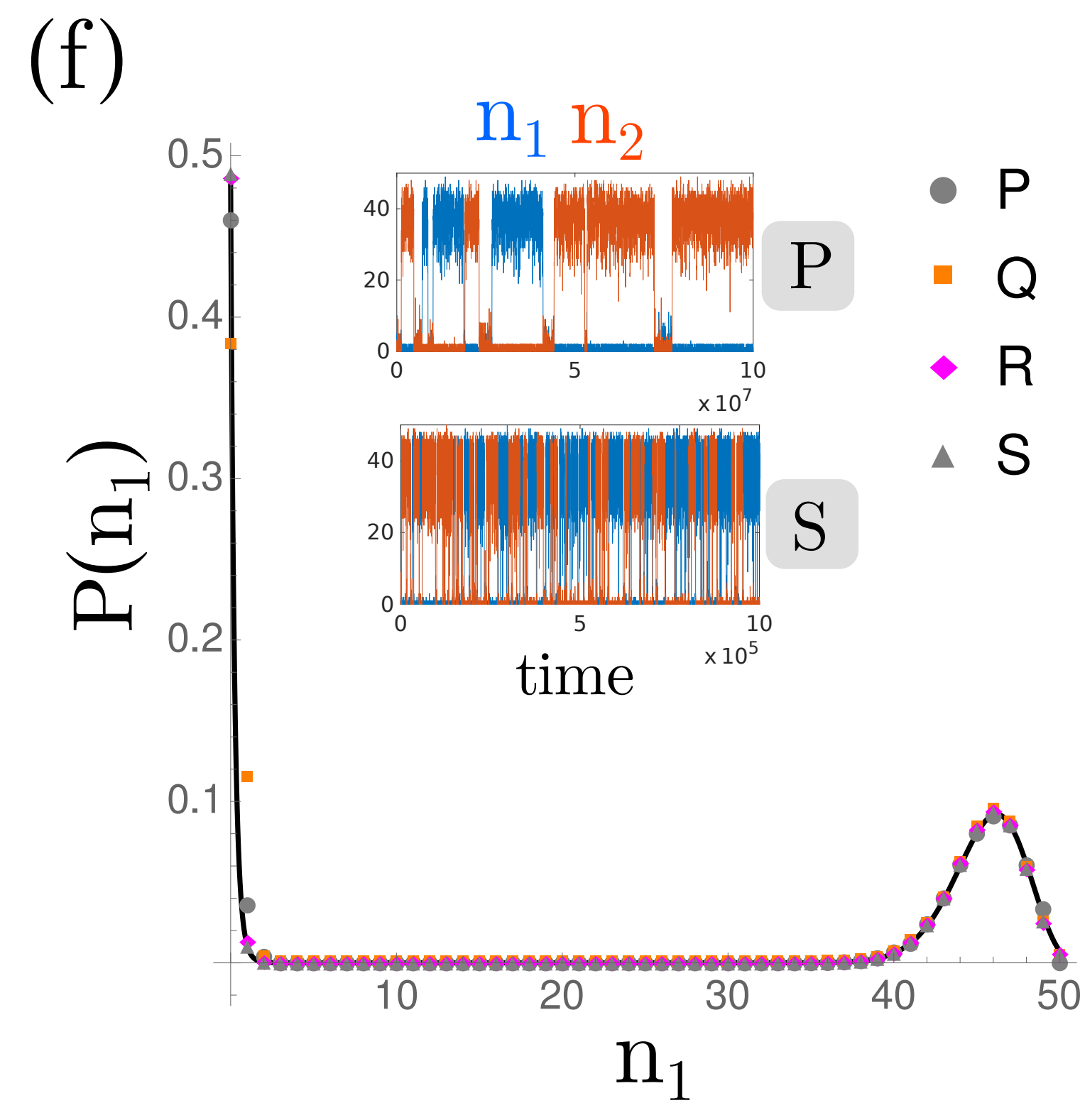

Supplement: S5 Fig — (A) We study the size dynamics of two growing structures with coefficients α and β satisfying the condition α + β = −1 (points P, Q, R and S). (B-E) The phase portrait in n1 and n2 plane show that there are two fixed points when β ≤ 0 (panels B-D), one saddle node and another unstable node. We obtain the additional boundary fixed points (open circles) by separately treating the divergence at the boundary. We get one stable and one unstable fixed points at the proximity of each boundary n1 = 0 and n2 = 0. In contrast when β > 0 (panel E), we find two stable nodes, leading to bistable size dynamics. (E, inset) There are four more fixed points at small size—one stable, one unstable and two saddle nodes. The stable point in small size has a very small basin of stability and it disappears at higher κ values. (F) The chemical master equation solution (solid line) and results from stochastic simulations (points) show that size distributions in all four cases are bimodal arising from bistability in size dynamics. Inset: Temporal evolution of the size of the structures in cases P and S, illustrating that residence times in the two stable states is dependent on the individual α and β values. For all calculations, N = 50 and κ1 = κ2 = κ = 0.005 (except in the last panel) were taken. (PDF) [file pcbi.1010253.s013.pdf]

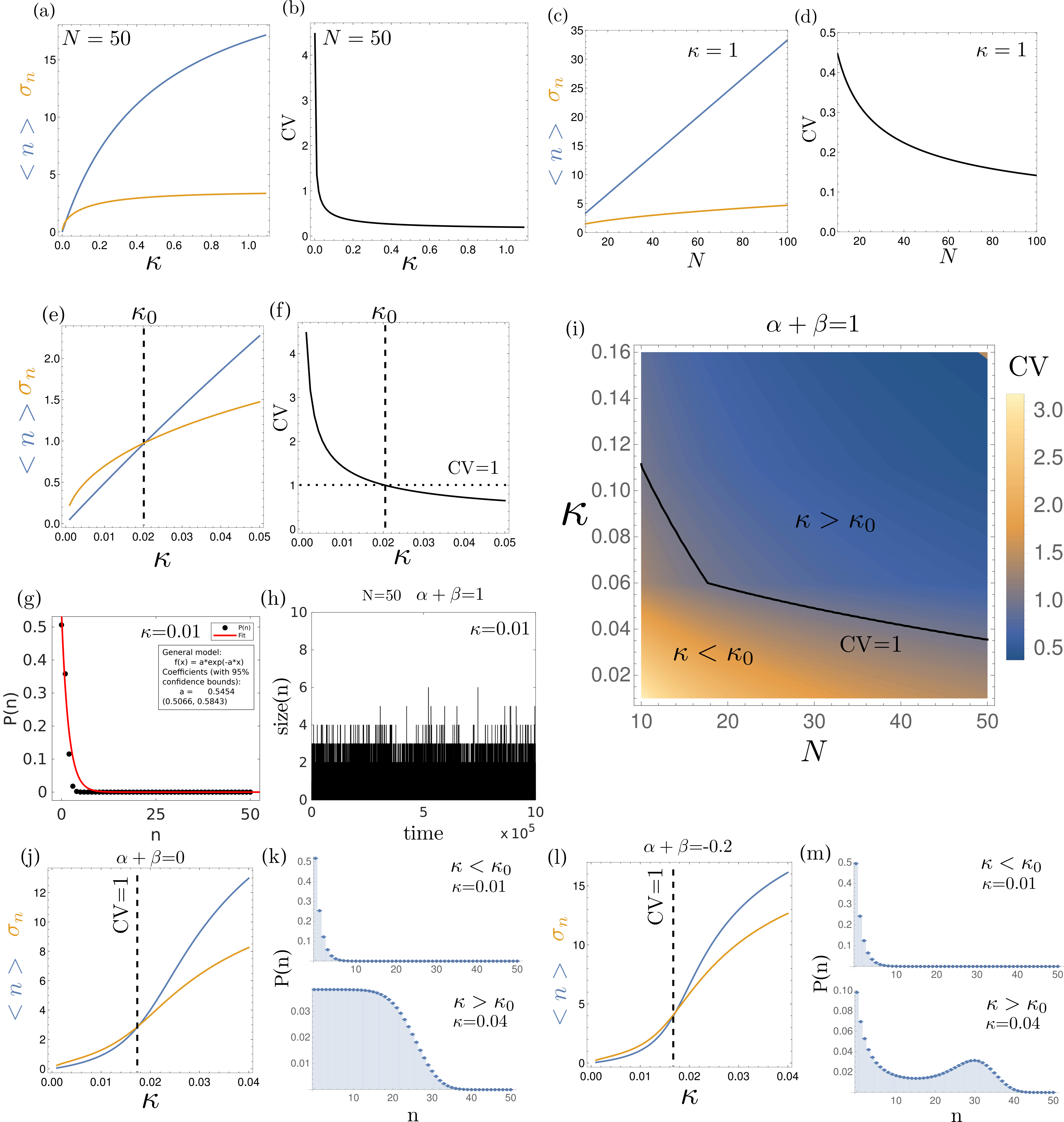

Supplement: S6 Fig — Here we show the failure of size regulation for small growth rate or subunit density. (A) Mean size and standard deviation in size increases monotonically and eventually saturates with increasing growth rate κ. (B) CV in size decreases as κ increases. The high CV value at small κ shows that the small structures at very low growth rates lacks robust control of size. (C) Mean size and standard deviation in size increases monotonically with increasing the total pool size N. (D) CV in size decreases as N increases. (E) Size fluctuations are larger than the mean size for very small values of growth rate κ. (F) CV is also larger than unity for small growth rates. We define the critical growth rate κ0 to be the growth rate where CV = 1. (G-H) The structures do not grow to be much larger in the low growth rate regime, and the size distribution can be fitted well to an exponential function, P(n) = λe−λn, where λ is a constant. (I) CV decreases with increasing growth rate κ and total pool size N, underlying a transition of size dynamics from no-growth to robust size regulation for κ > κ0. The κ0 value decreases as the total pool size increases indicating that this transition in size dynamics can occur due to reduction in subunit density. For all the results discussed up to this point we take α + β = 1. (J-K) Large size fluctuations compared to the mean size and a characteristic exponential size distribution for κ < κ0 is also present in the limit α + β = 0. (L-M) Large size fluctuations compared to the mean size and a characteristic exponential size distribution for κ < κ0 is also seen in the case of autocatalytic growth (α + β < 0). The effects of feedback in the growth becomes apparent when κ > κ0. These results were obtained from the solution to the master equation for two growing structures with N = 50. (PDF) [file pcbi.1010253.s014.pdf]

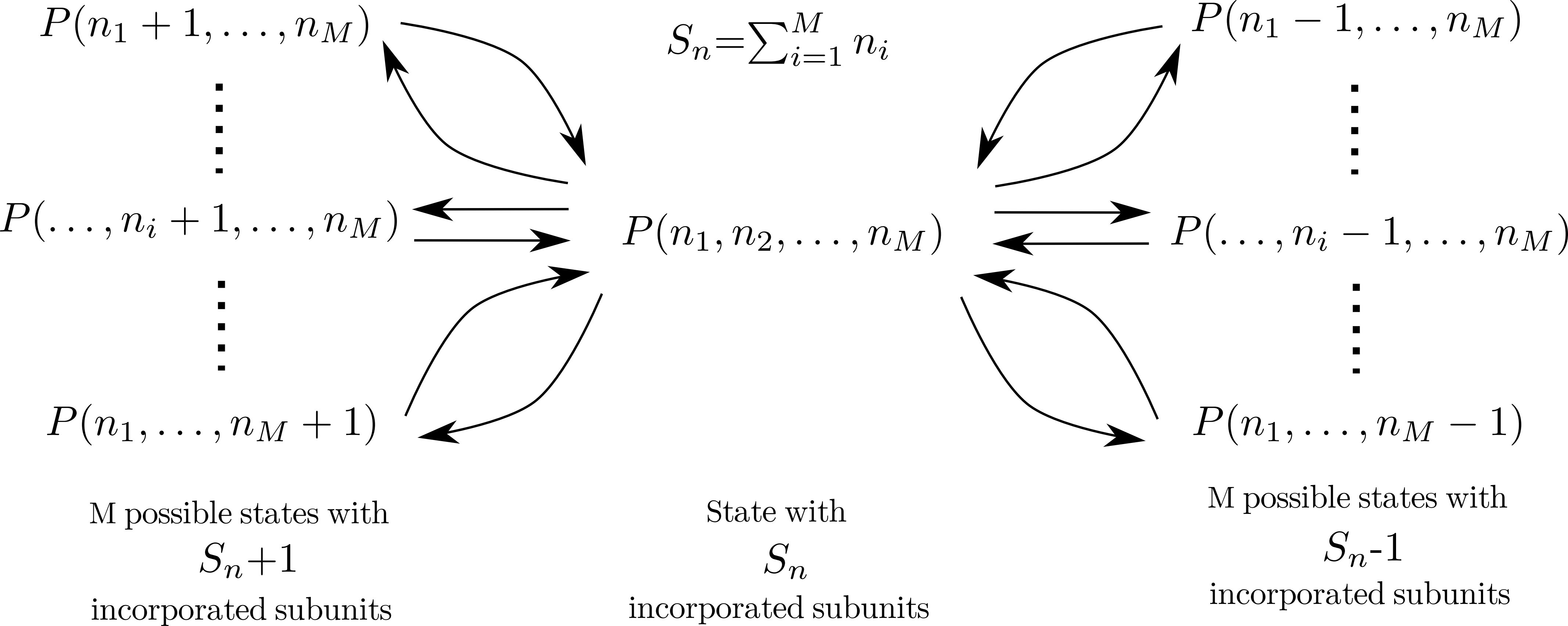

Supplement: S7 Fig — Illustration of all possible transitions into and from the state {n1, n2, …, nM} via assembly and disassembly processes. (PDF) [file pcbi.1010253.s015.pdf]

M=2

(a)

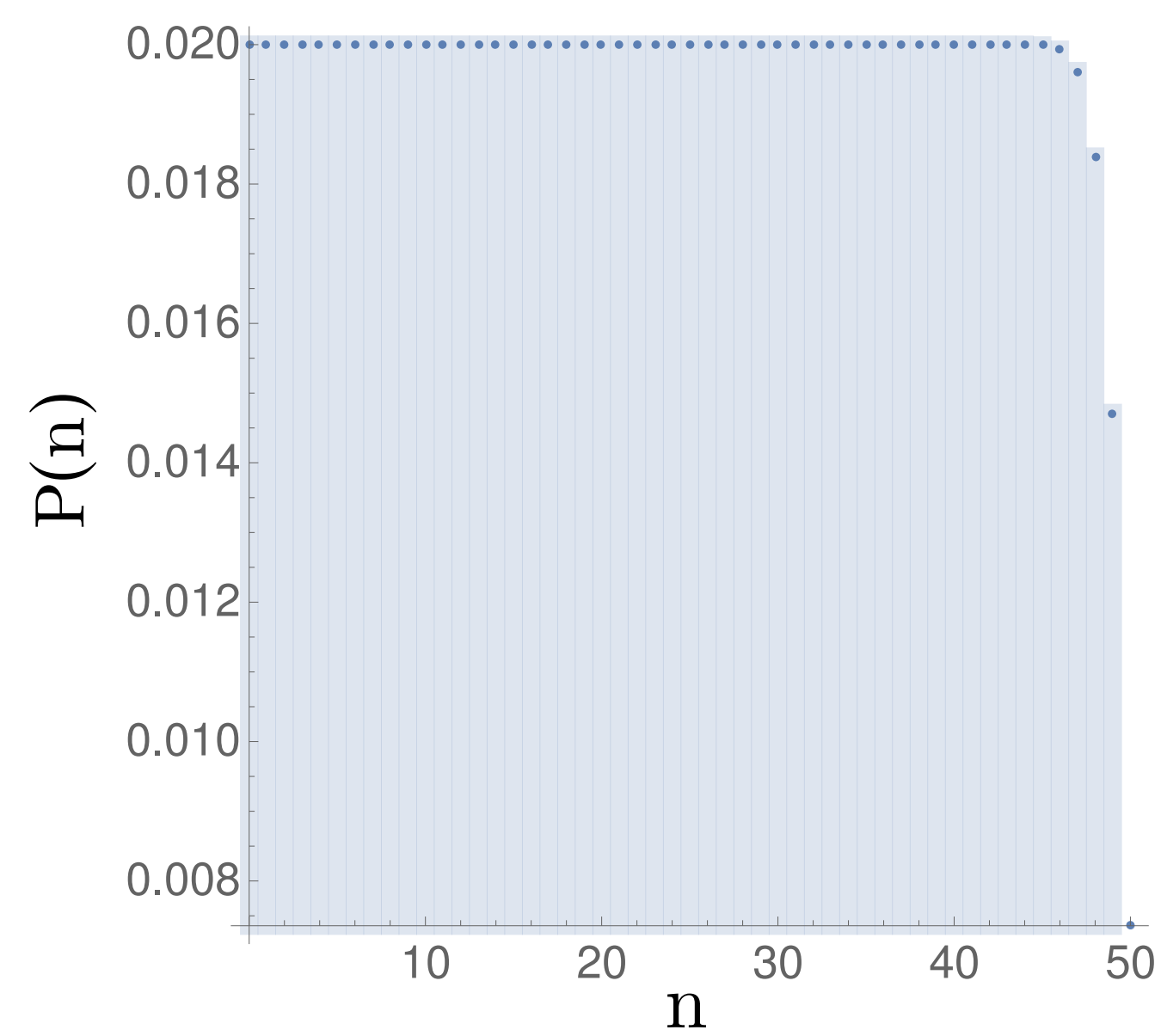

M=3

(b)

 $\alpha + \beta = 0$ 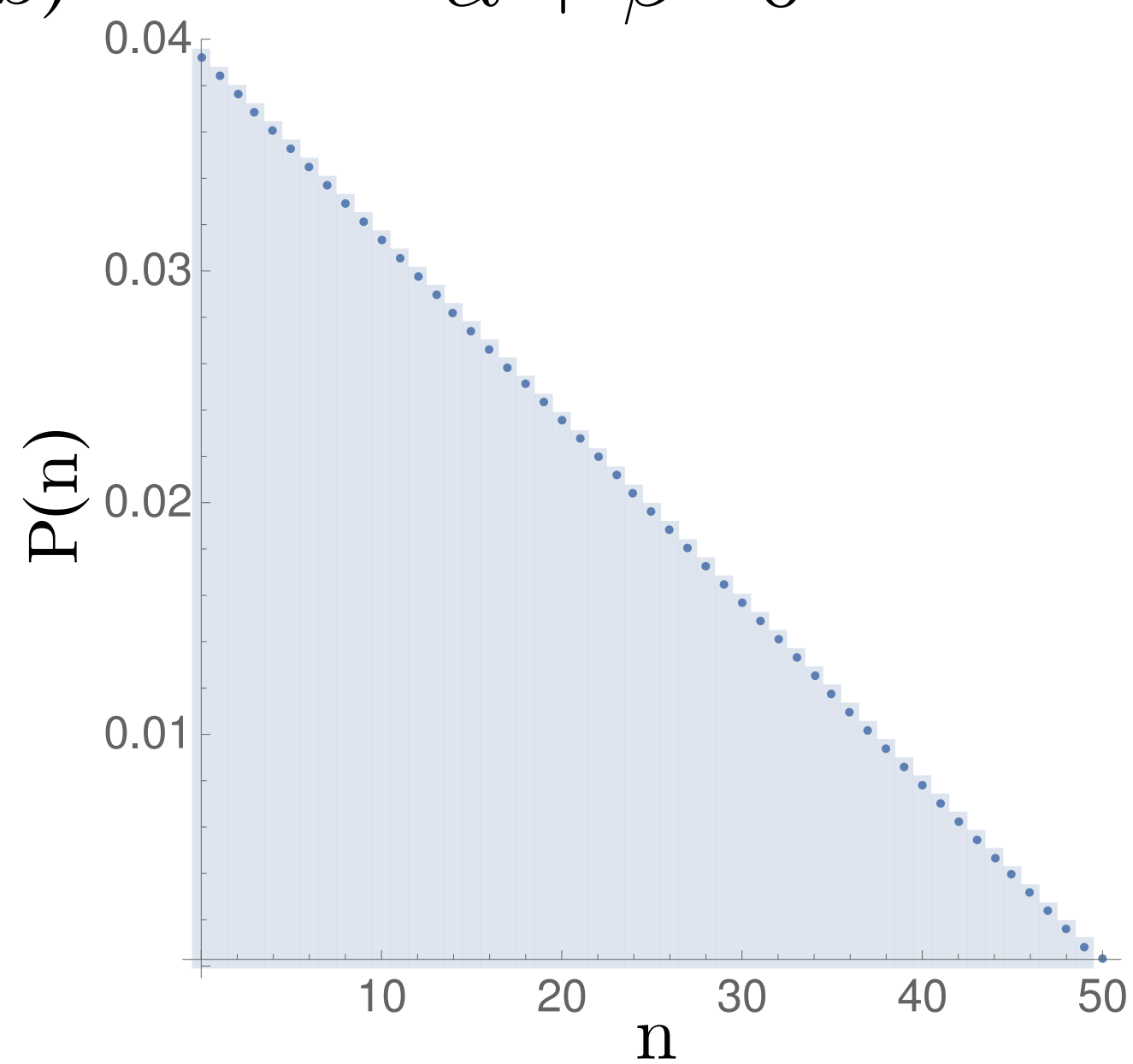

M=4

(c)

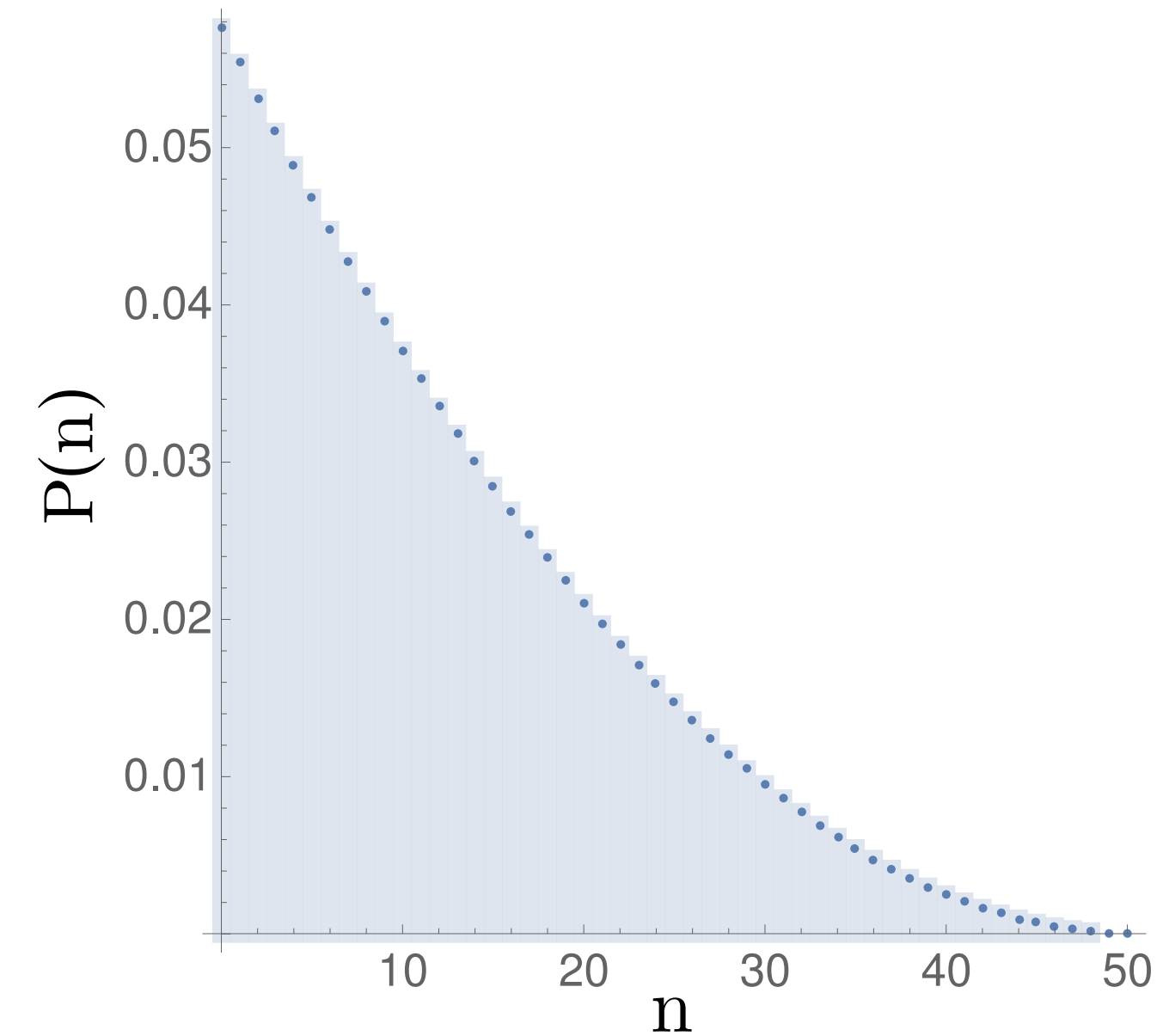

(d)

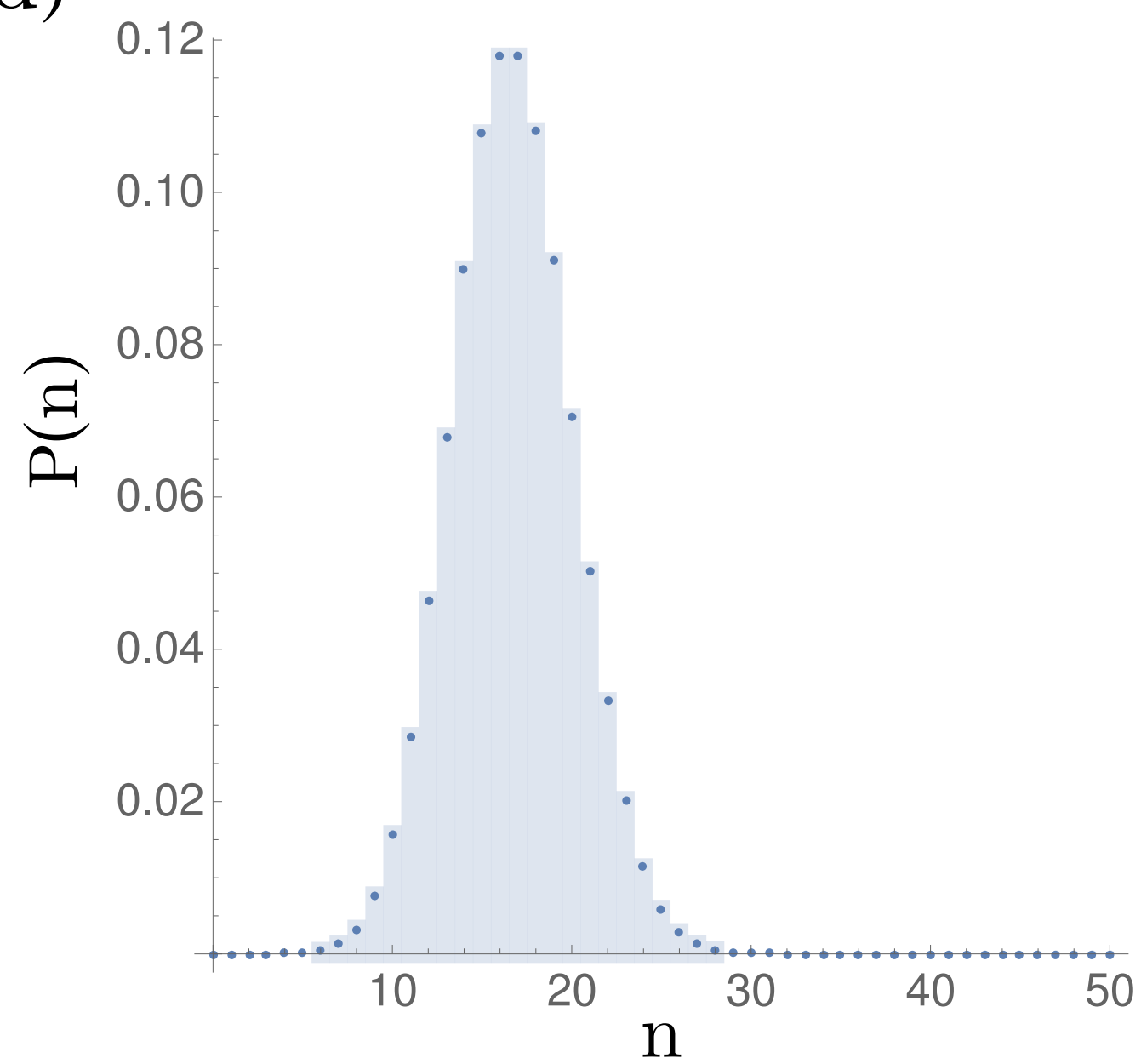

(e)

 $\alpha + \beta = 1$ 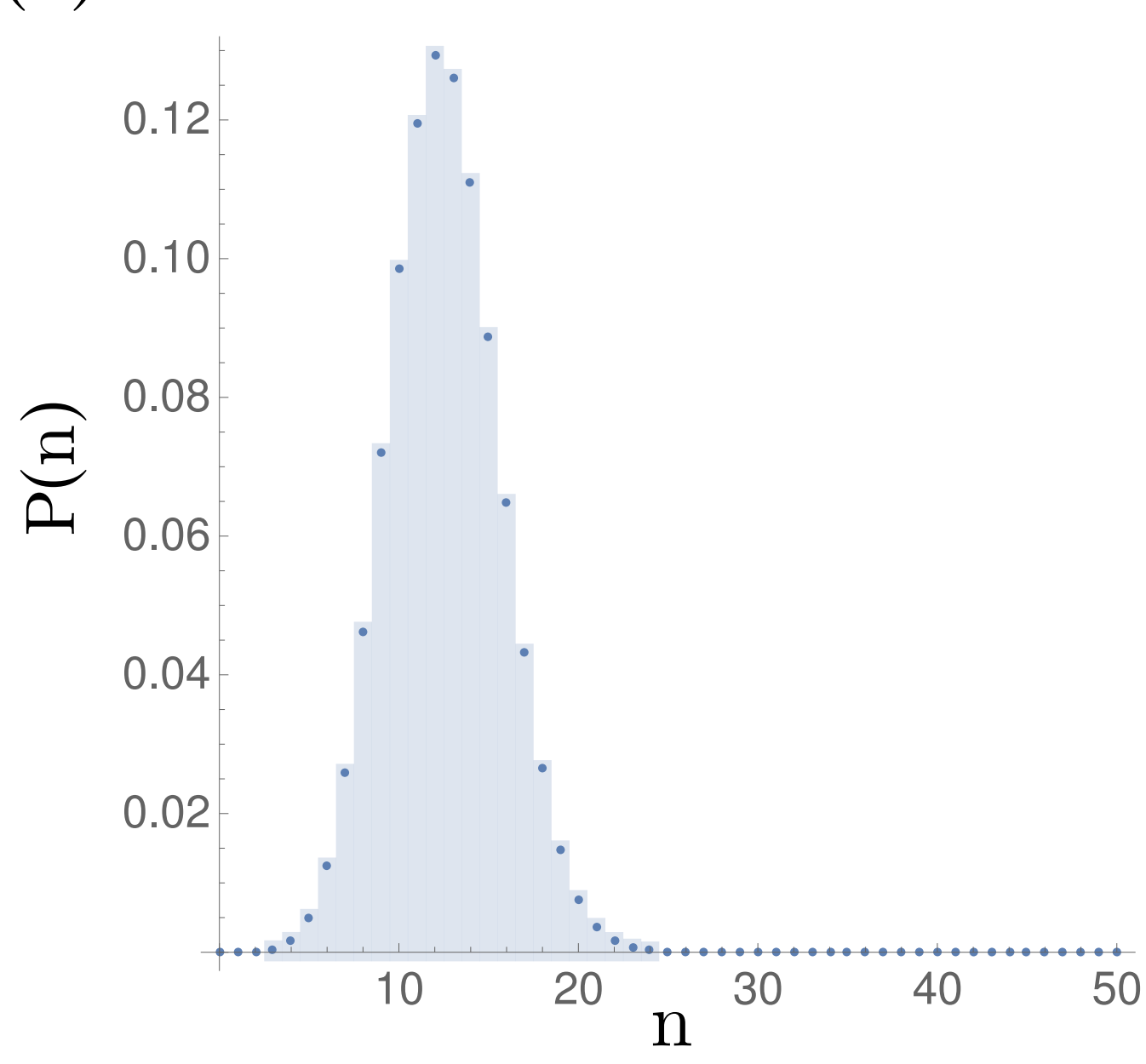

(f)

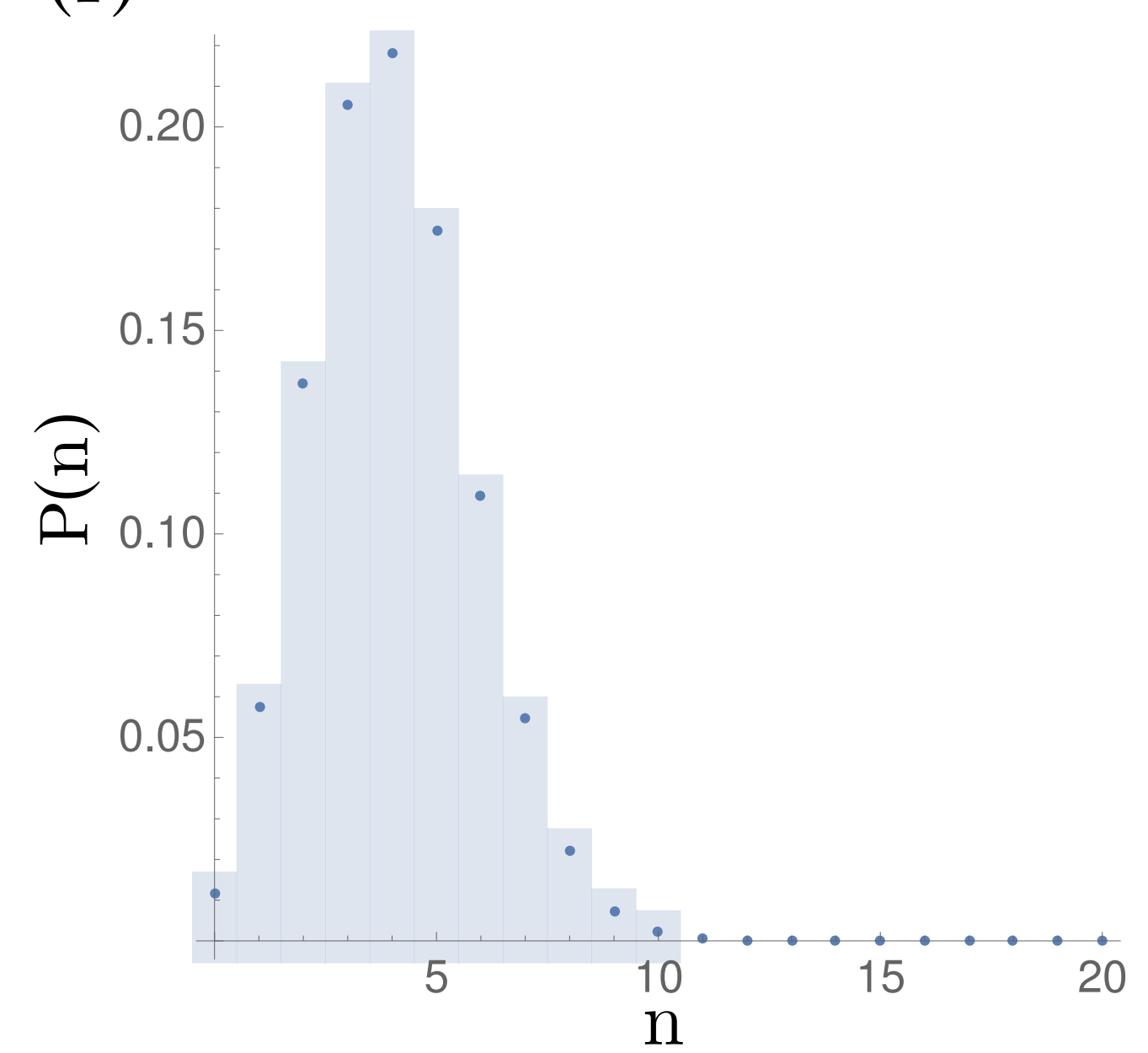

(g)

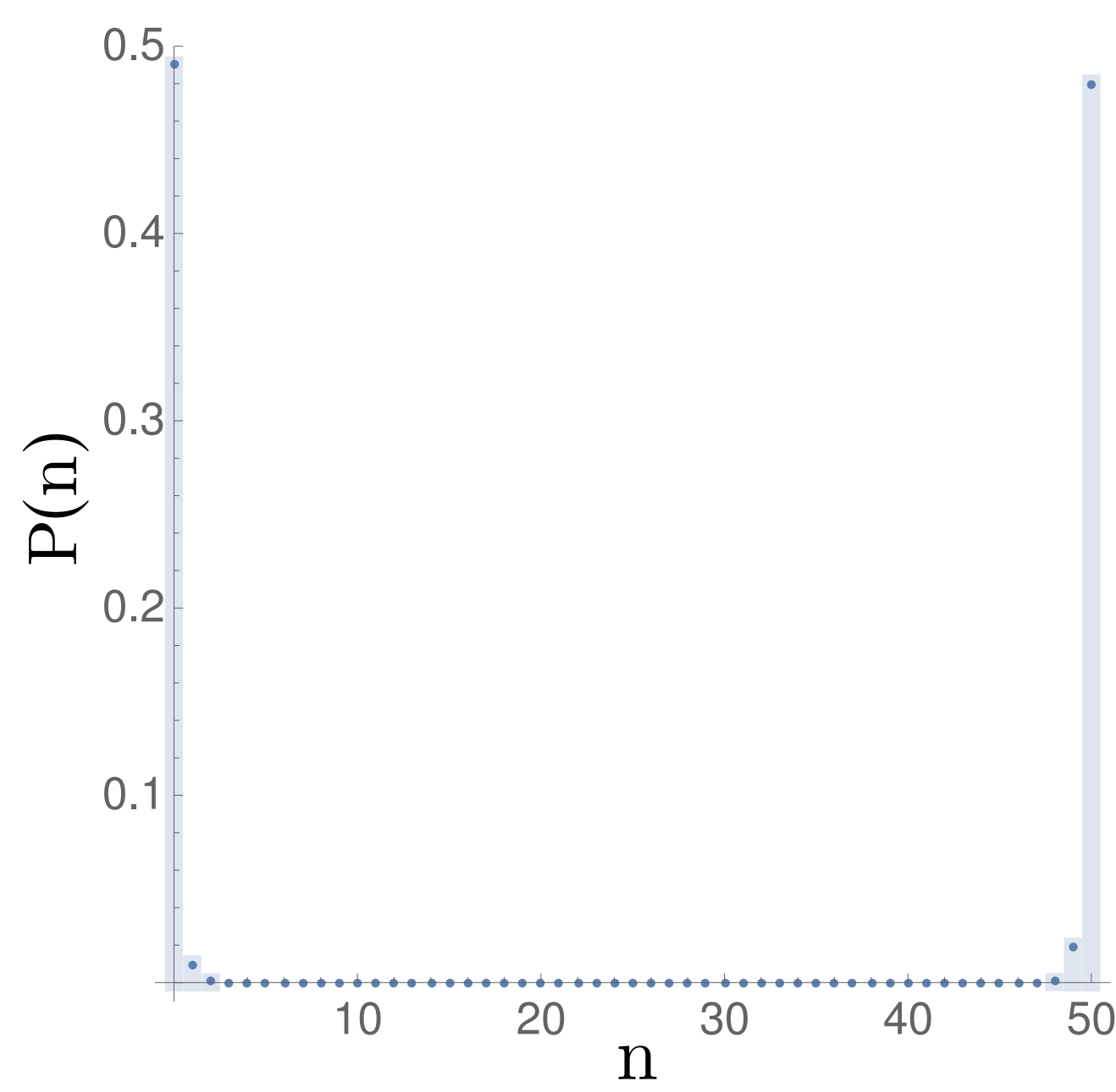

(h)

 $\alpha + \beta = -1$ 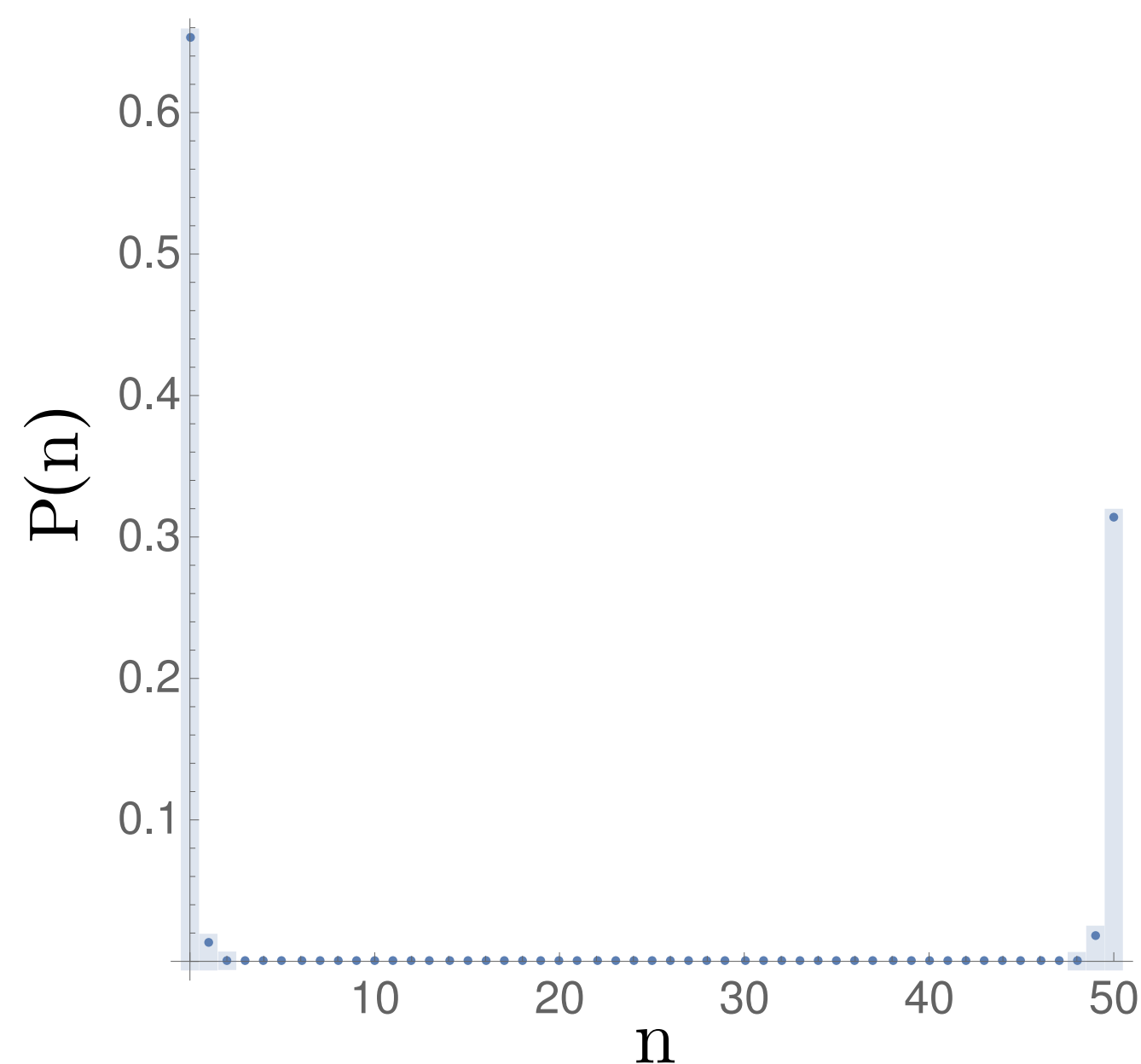

(i)

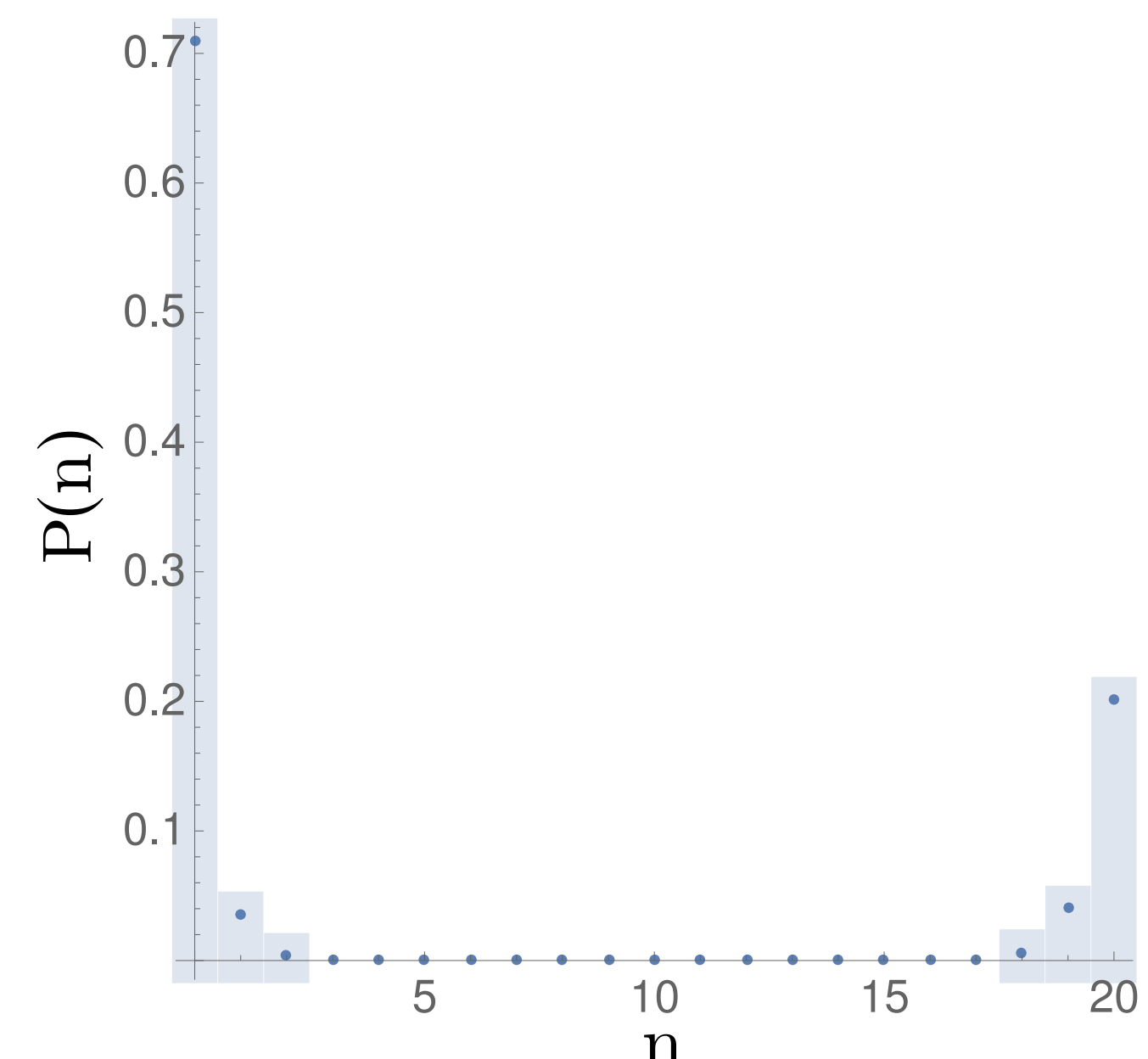

Supplement: S8 Fig — (A-C) Individual size distribution of M identical structures in the limit α + β = 0. (D-F) With negative feedback control of growth (α + β > 0), robust size regulation is achieved for any M number of structures, with the mean size decreasing with increasing M with fixed pool size N. (G-I) The size dynamics is bistable in the presence of positive feedback, α + β < 0. The parameter values are N = 50, κ = 1 for all cases except (F) and (I), where N = 20. (PDF) [file pcbi.1010253.s016.pdf]

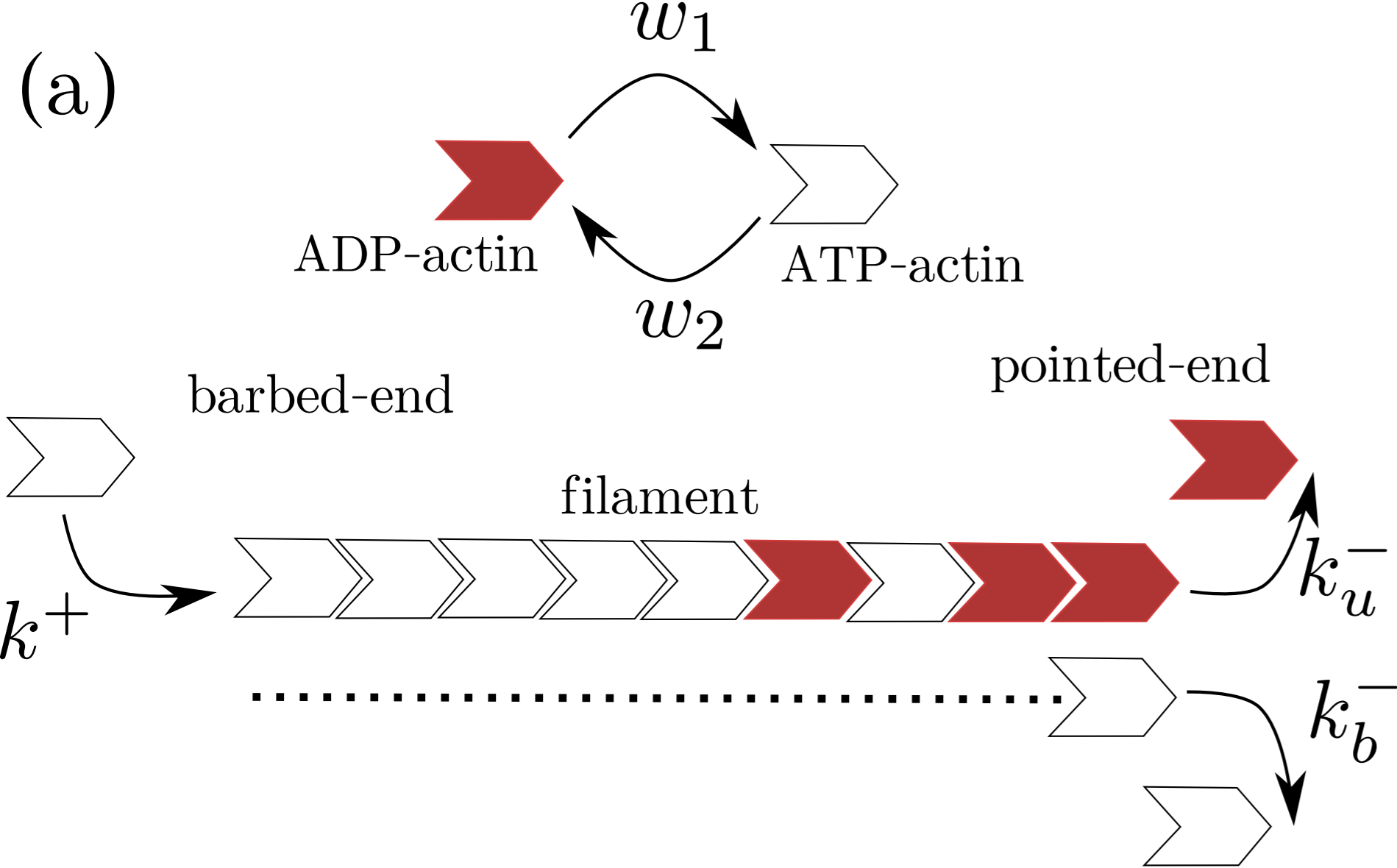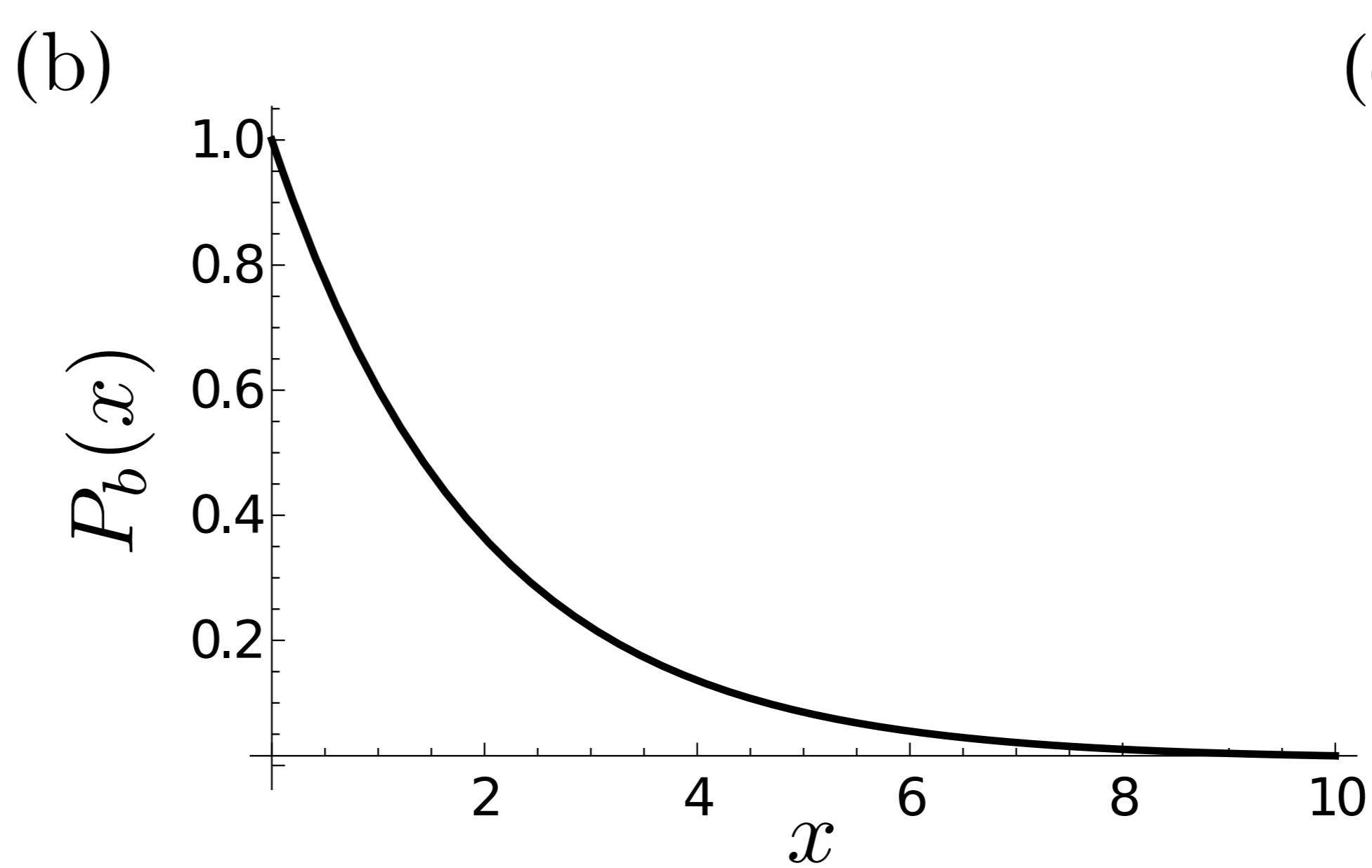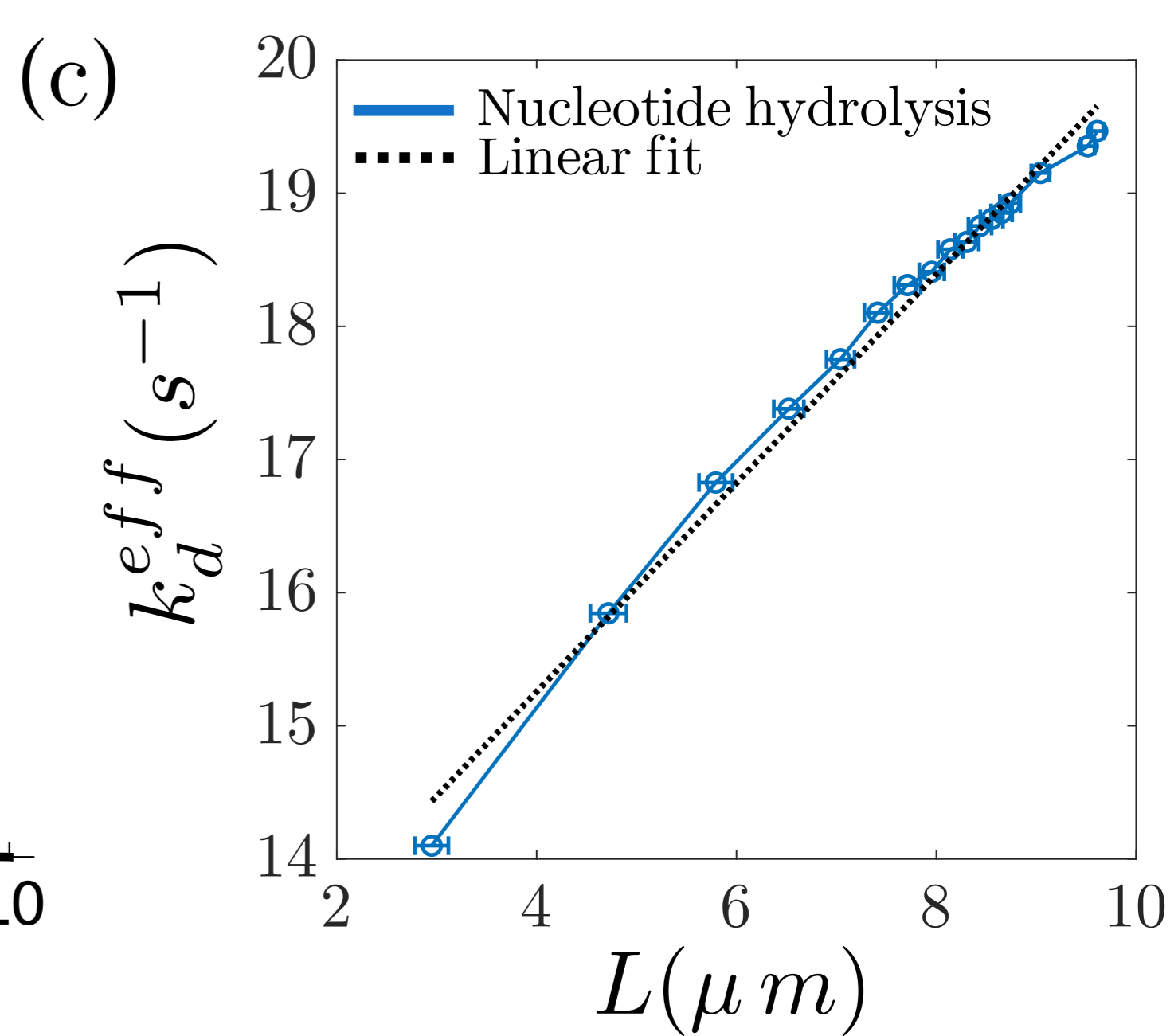

Supplement: S9 Fig — Length dependent disassembly rate arises when monomers switch between different states with distinct disassembly rates. (A) Schematic of filament growth with monomer state switching. (B) At steady-state the probability of ATP-bound monomer Pb(x) decreases towards the pointed end with a length scale λ−1. Parameters: k+ = 2, kb-=0.5, ku-=2.5, w1 = 0.01 and w2 = 0.02. (C) The effective disassembly rate of the filament, computed from a stochastic simulation (using Gillespie algorithm) of filament growth with nucleotide hydrolysis and in a limiting subunit pool. kdeff(L) increases linearly with filament length. Parameters: kb-=10 s−1, ku-=200 s−1, ρ0 ≃ 3 μM, w1 = 0.005 s−1 and w2 = 0.01 s−1. The parameter k+ was varied to span the range of filament lengths. (PDF) [file pcbi.1010253.s017.pdf]

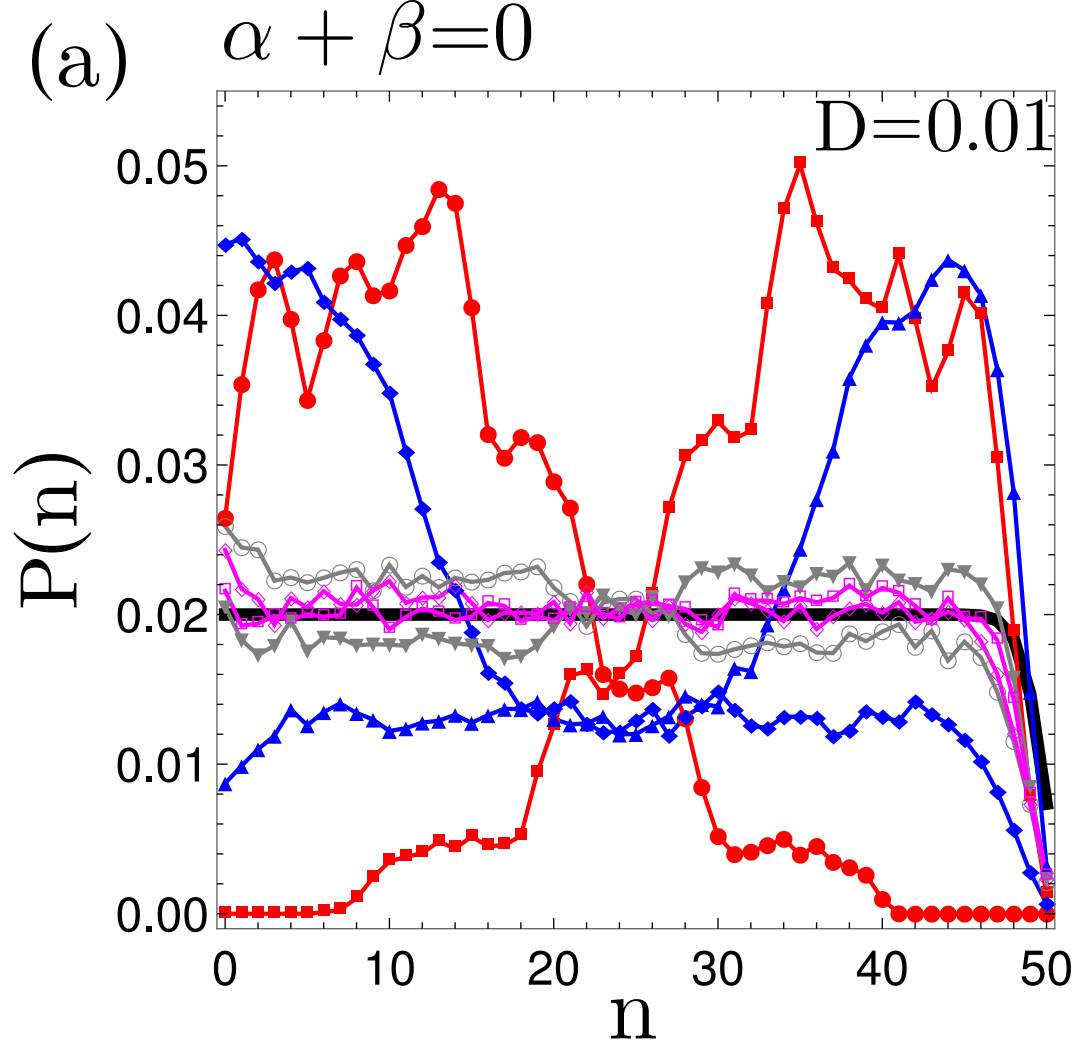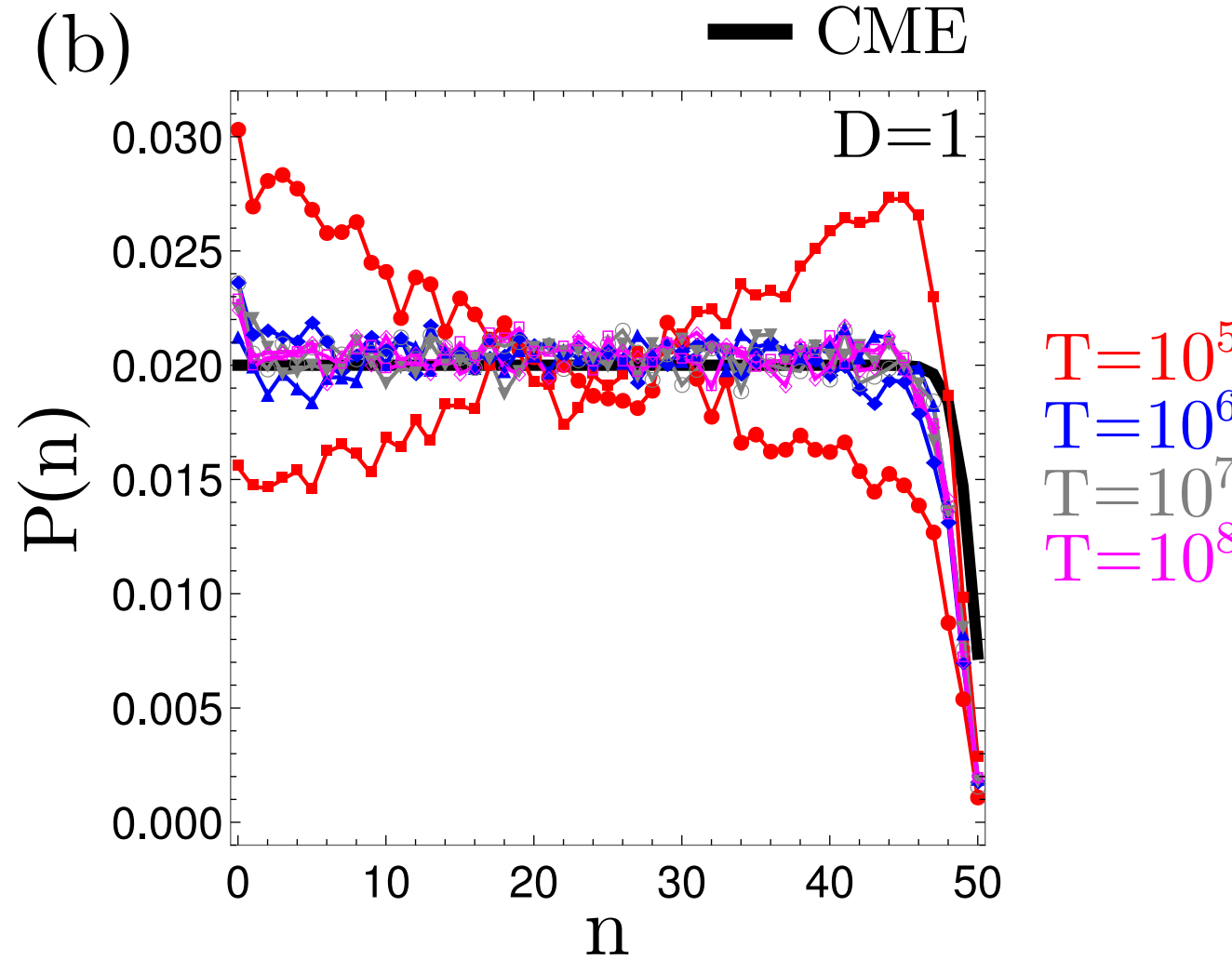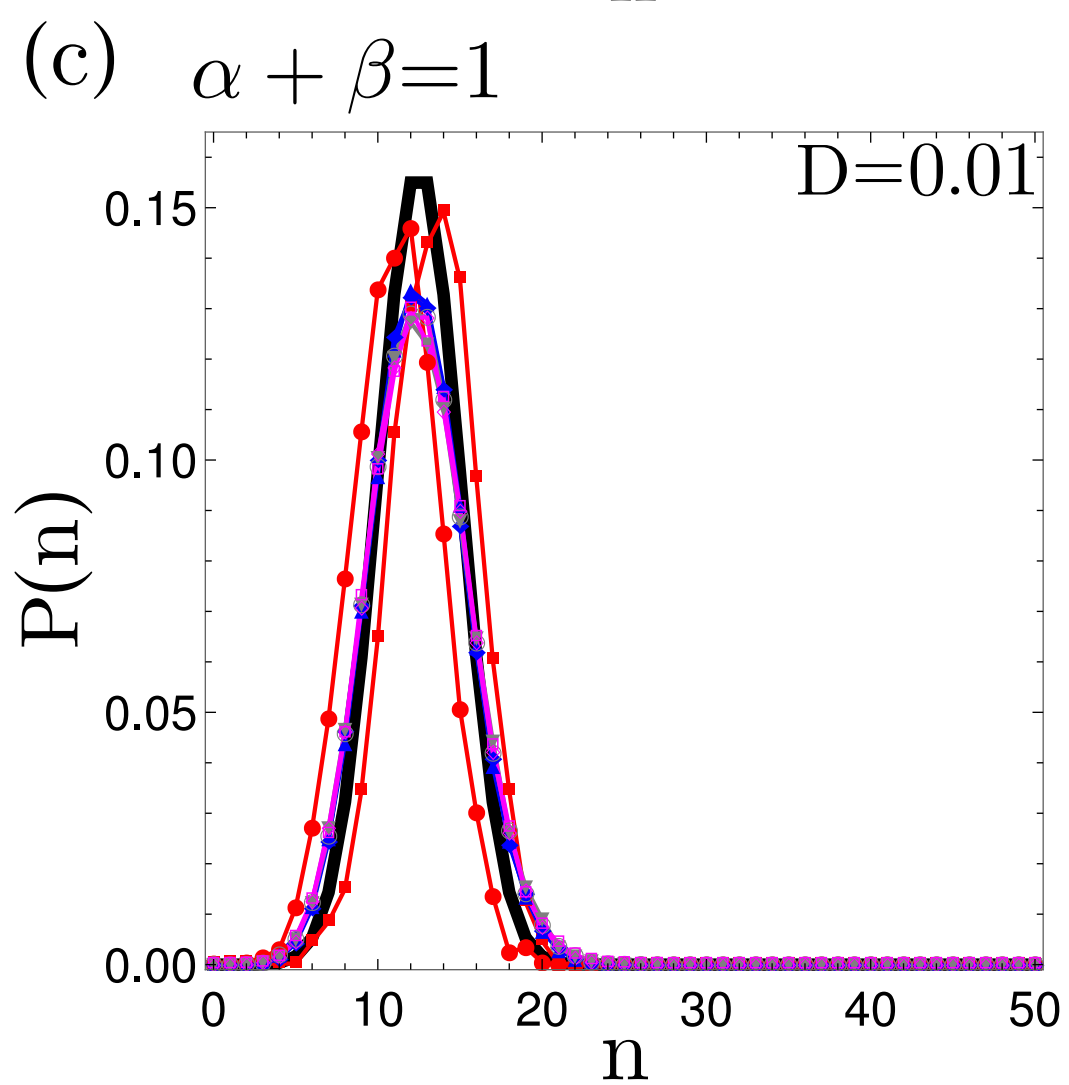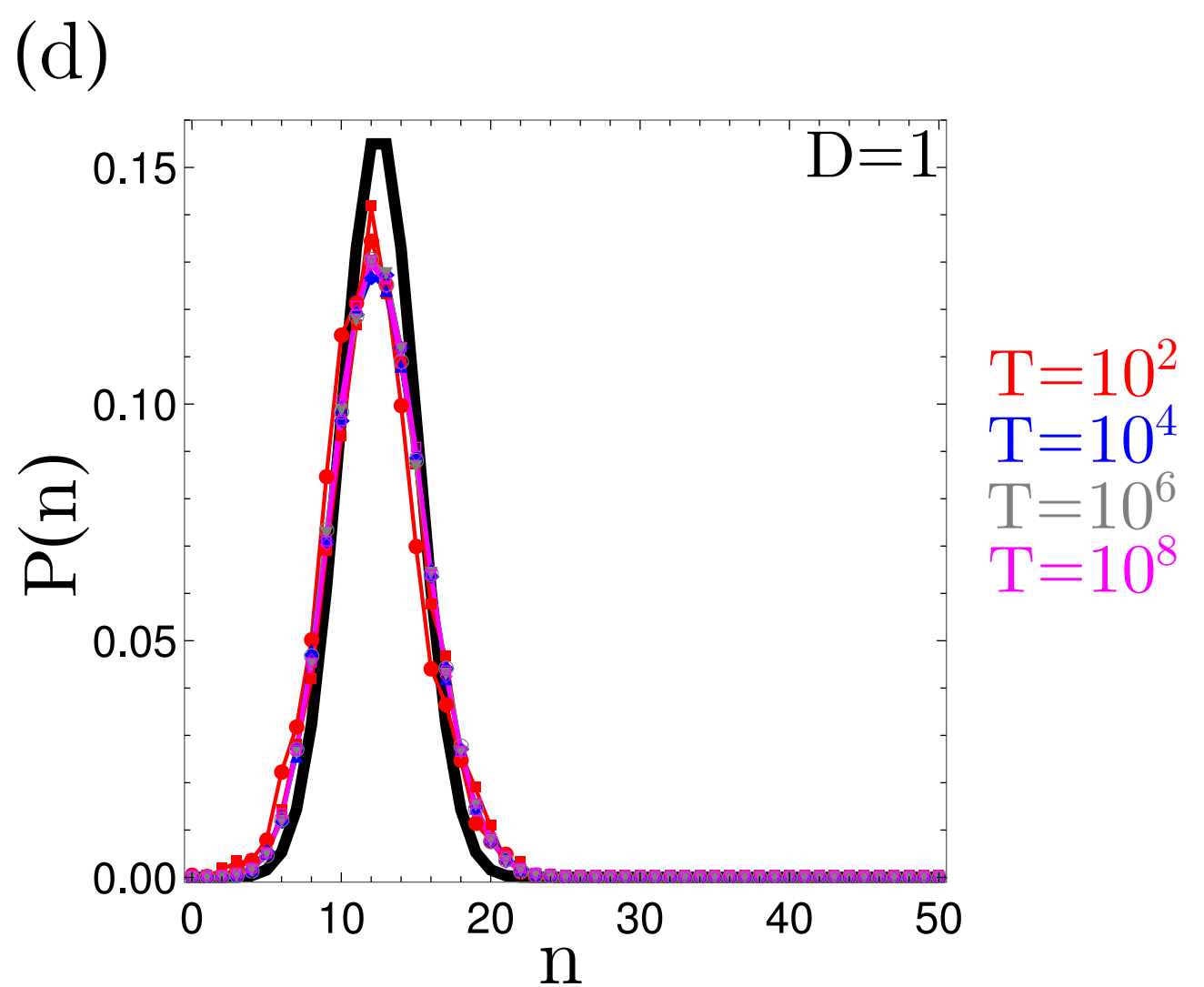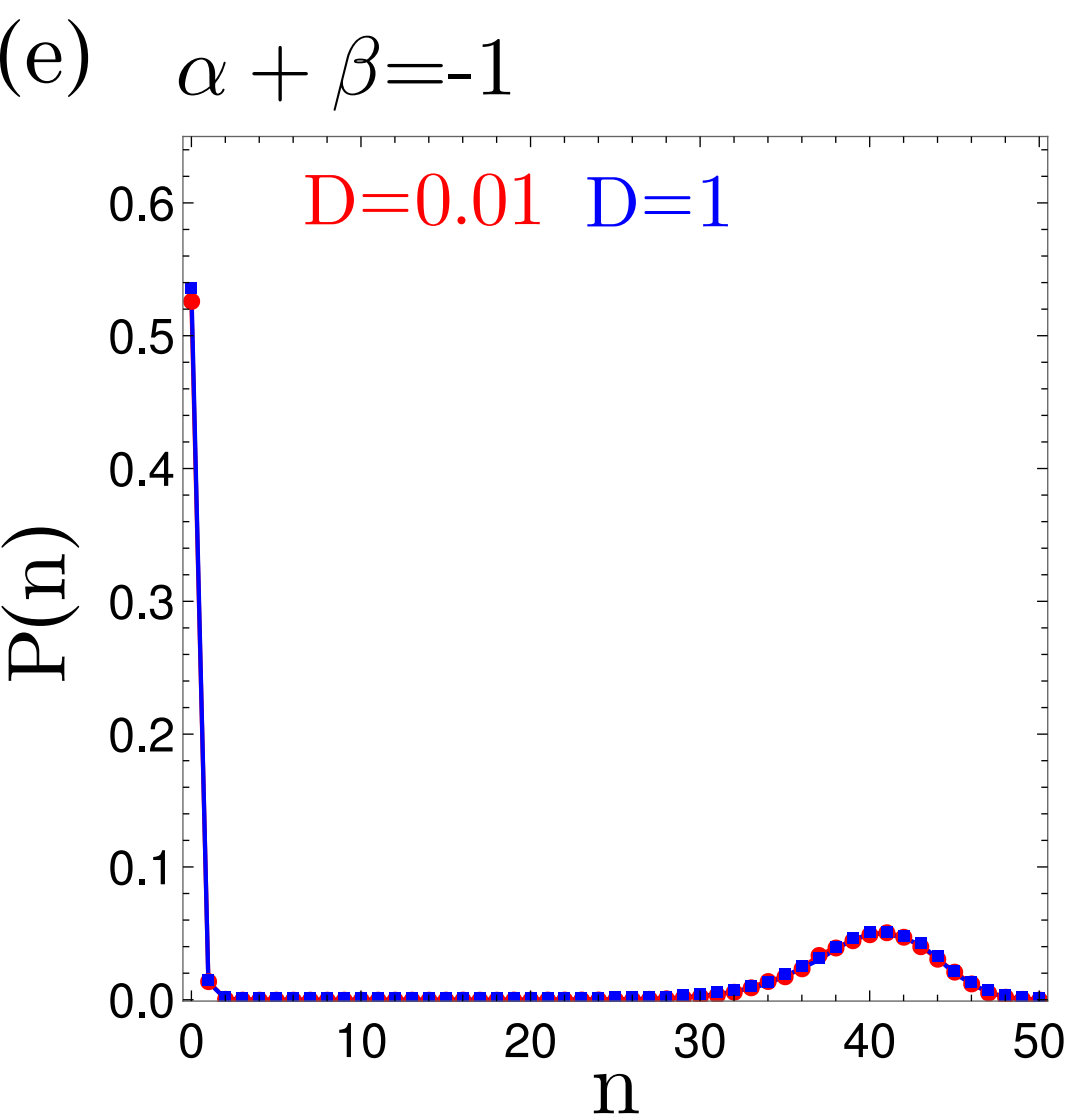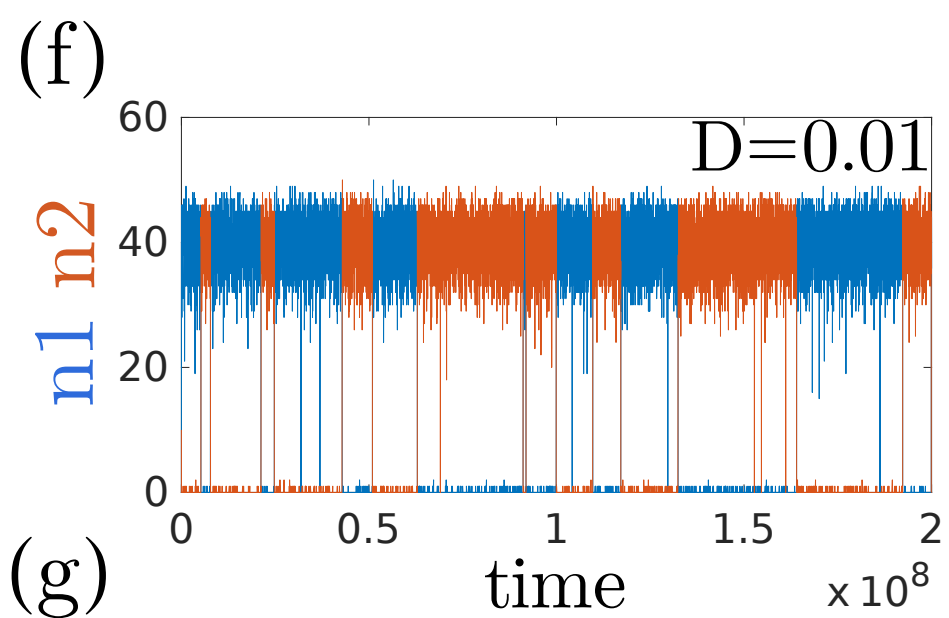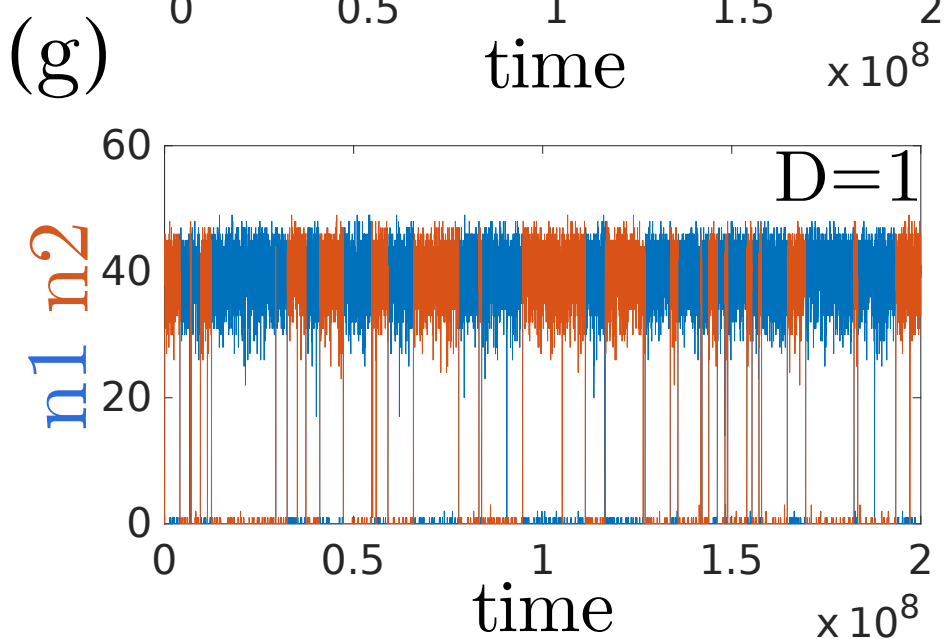

Supplement: S10 Fig — (A,B) Size regulation fails in the parameter regime α + β = 0 in the presence of diffusion. In the case of slower diffusion, the size distribution takes longer time (T, in simulation units) to converge to the almost uniform size distribution predicted by solution to the chemical master equation. The colours indicate the simulation time T, and the curves of same color represent the size distribution of the two structures that converge to the same distribution at longer times. (C,D) In presence of negative feedback, α + β > 0, the size distribution quickly relaxes to the steady state distribution and this relaxation timescale does not depend on the diffusion constant. (E) In presence of positive feedback, α + β < 0, we see bistability and the resulting bimodal size distribution does not depend on the value of the diffusion constant. (F,G) The diffusion affects the statistical properties of the size dynamics. A slower diffusion (F) will lead to a longer residence time in one of the steady states. The parameter values are N = 50 and κ = 1 everywhere except in (E-G) where κ = 0.005. (PDF) [file pcbi.1010253.s018.pdf]

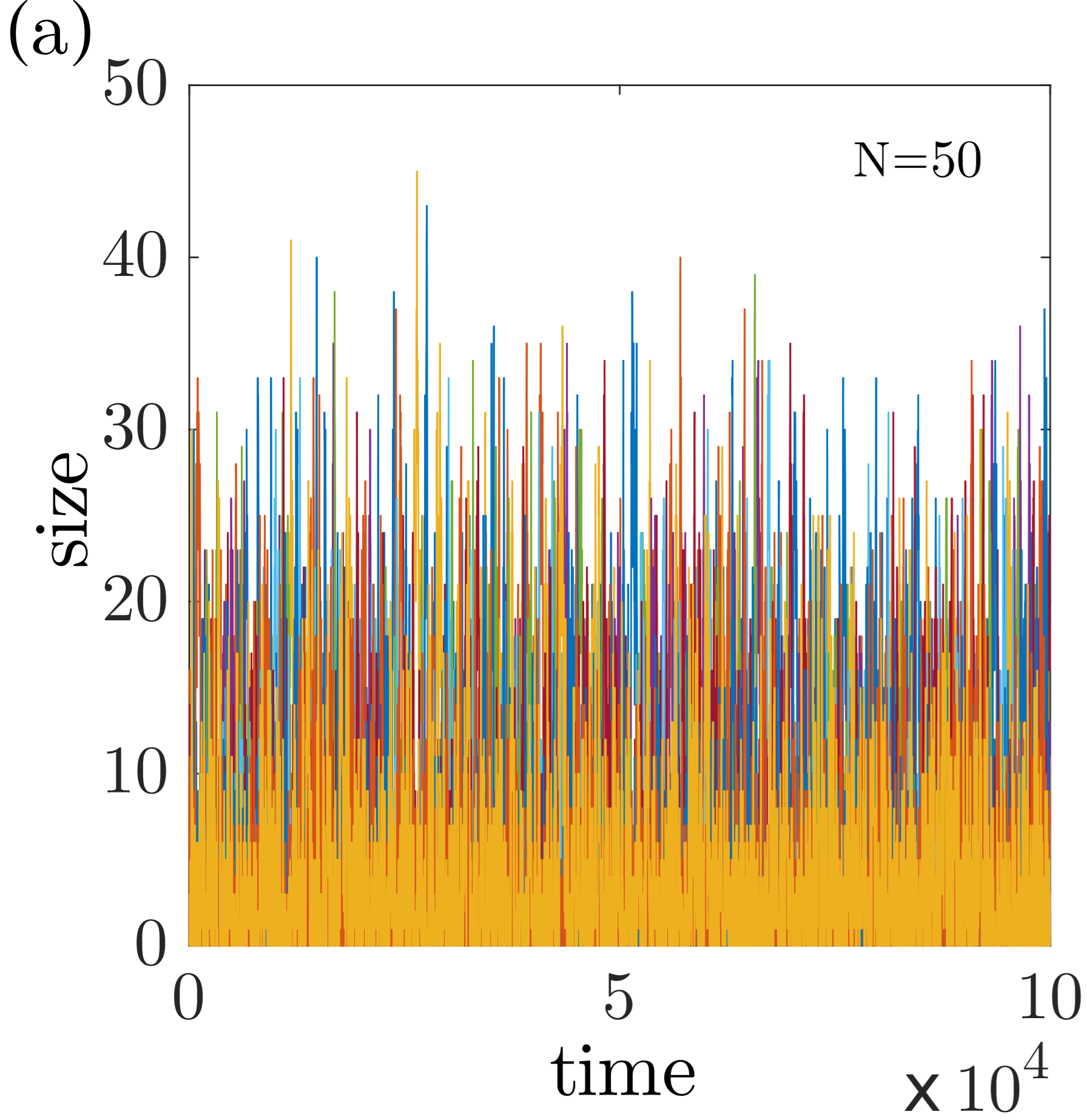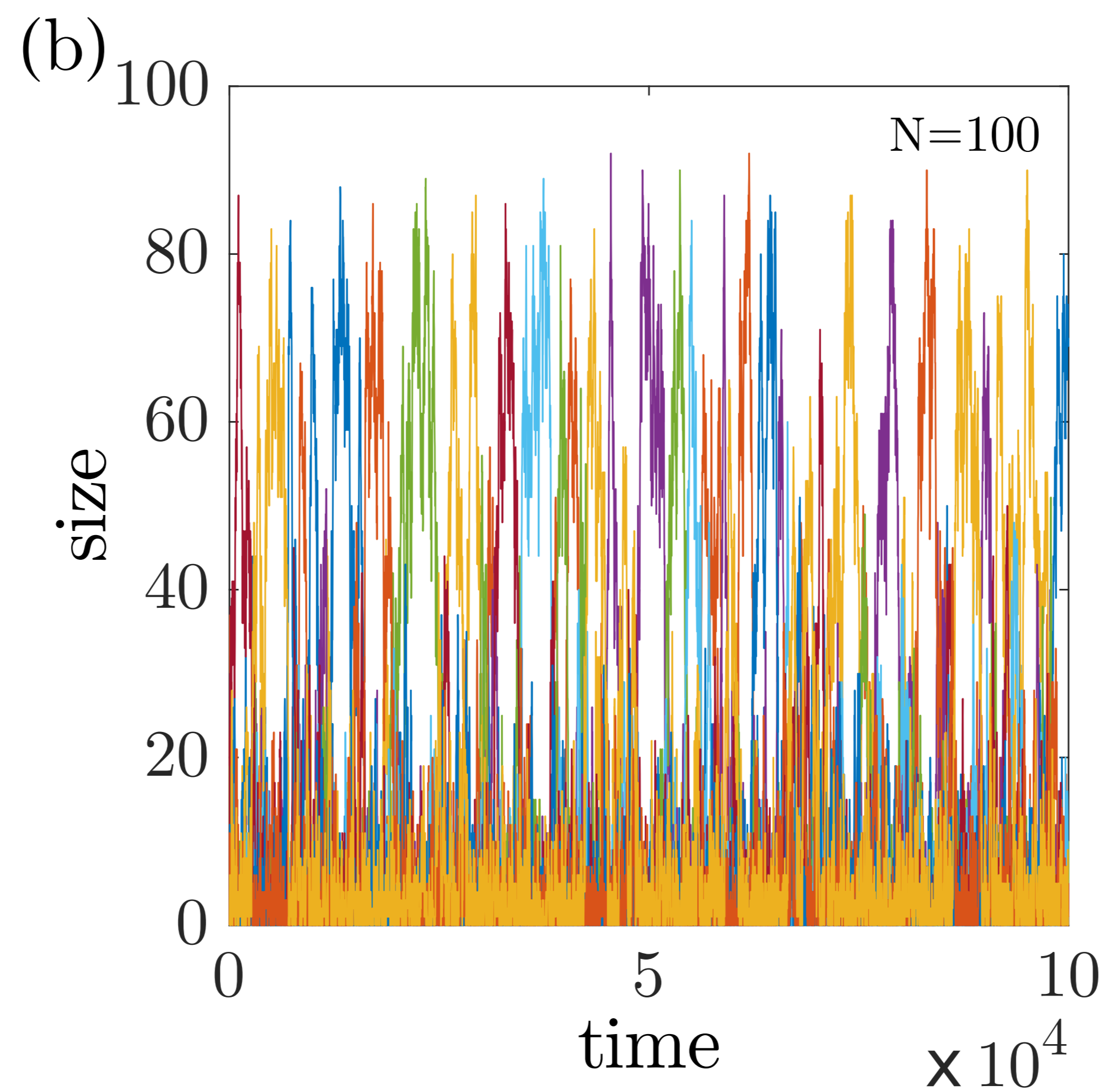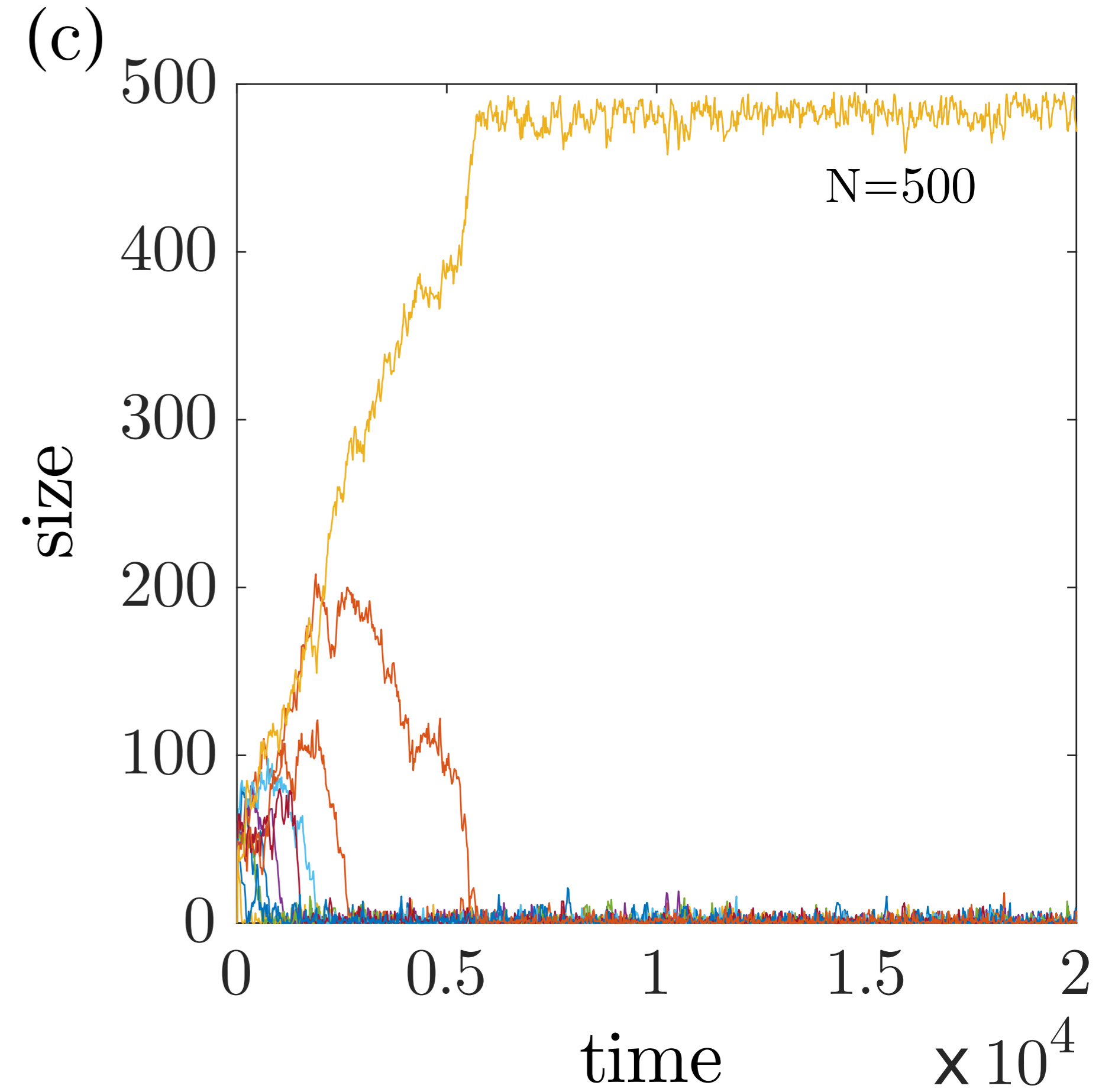

Supplement: S11 Fig — (A-C) We study growth of multiple structures from a common pool of subunits, with size-dependent positive feedback α + β = −0.2. This makes the growth process autocatalytic. We see a transition in the size dynamics as we increase the overall density of subunits (by changing total amount N while keeping volume V fixed). (A) The structures hardly grow at low subunit density. (B) At intermediate subunit density, bistability in size distribution emerges. The bigger structure captures most of the subunits, but suddenly starts declining in size due to stochastic fluctuations, when the other structures grow to be bigger. This creates a “flickering” growth pattern of multiple structures. (C) At a higher subunit density, we observe an initial growth of multiple structures. At a later time only a single structure forms while the others “die-out” in the competition. This mechanism can be used to make sure only a single structure gets built inside the cell which is important in various cases of polarity establishment and spontaneous symmetry breaking. (PDF) [file pcbi.1010253.s019.pdf]

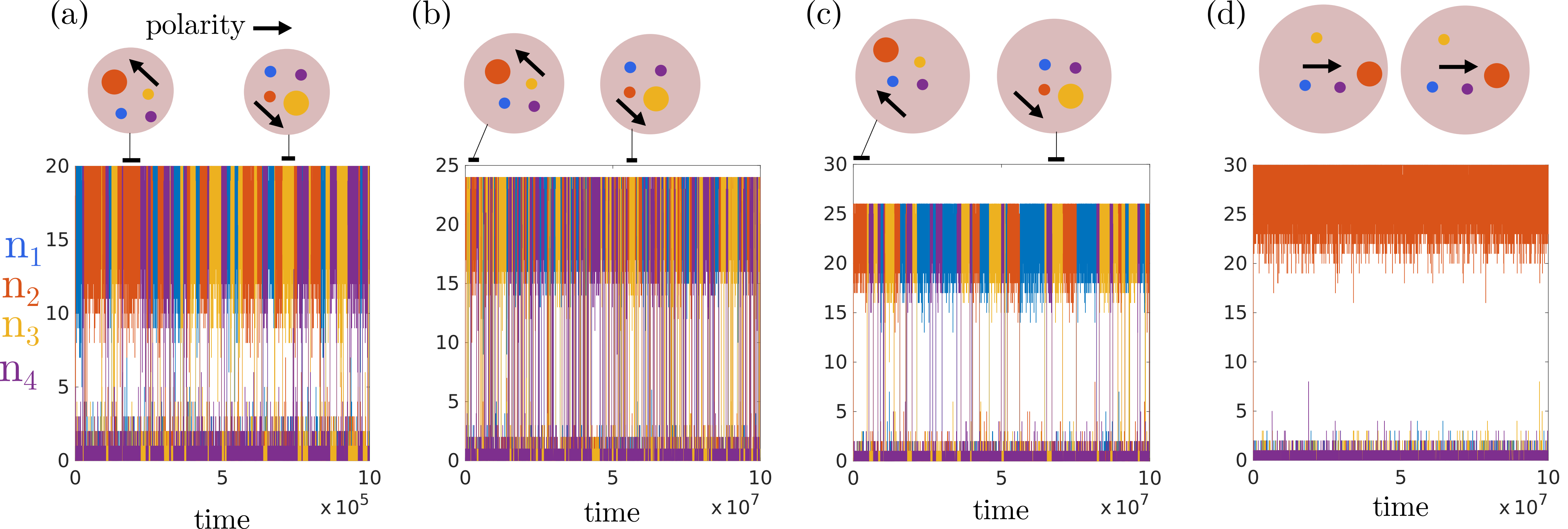

Supplement: S12 Fig — (A-D) We study growth of multiple structures (M = 4) from a common pool of subunits in a system size V, with size-dependent positive feedback α = −1, β = 0. This makes the growth process autocatalytic. We see a transition in the size dynamics as we increase the system size V while keeping the subunit density the same. This shows a size dependent polarity establishment process where below a critical size of the system all structures grow and shrink dynamically. But above a critical size, the residence time becomes exceedingly large making transitions virtually impossible in an experimentally relevant timescale. Thus only a stochastically selected structure remain in large size, establishing a polarity in the cell. The pool size N and cell volume V was changed 20%, 30% and 50% in panels B,C and D, respectively. Parameters (for panel A): N = 20, V = 1, κ = 0.02. (PDF) [file pcbi.1010253.s020.pdf]
